# Modeling multi-contact point physical interaction between the anthropomorphic finger and soft robotic exo-digit for wearable rehabilitation robotics applications

**DOI:** 10.3389/frobt.2023.1209609

**Published:** 2023-11-17

**Authors:** Umme Kawsar Alam, Kassidy Shedd, Joshua Kirkland, Kayla Yaksich, Mahdi Haghshenas-Jaryani

**Affiliations:** ^1^ Bio^2^Robotics Laboratory, Department of Mechanical and Aerospace Engineering, New Mexico State University, Las Cruces, NM, United States; ^2^ Business Administration Department, College of Business, New Mexico State University, Las Cruces, NM, United States

**Keywords:** quasi-static model, soft exo-digit, physical human–robot interaction, free bending motion, constrained bending motion, hyperelastic constitute model, soft actuator lengthening, distributed interaction

## Abstract

**Introduction:** Effective control of rehabilitation robots requires considering the distributed and multi-contact point physical human–robot interaction and users’ biomechanical variation. This paper presents a quasi-static model for the motion of a soft robotic exo-digit while physically interacting with an anthropomorphic finger model for physical therapy.

**Methods:** Quasi-static analytical models were developed for modeling the motion of the soft robot, the anthropomorphic finger, and their coupled physical interaction. An intertwining of kinematics and quasi-static motion was studied to model the distributed (multiple contact points) interaction between the robot and a human finger model. The anthropomorphic finger was modeled as an articulated multi-rigid body structure with multi-contact point interaction. The soft robot was modeled as an articulated hybrid soft-and-rigid model with a constant bending curvature and a constant length for each soft segment. A hyperelastic constitute model based on Yeoh’s 3rdorder material model was used for modeling the soft elastomer. The developed models were experimentally evaluated for 1) free motion of individual soft actuators and 2) constrained motion of the soft robotic exo-digit and anthropomorphic finger model.

**Results and Discussion:** Simulation and experimental results were compared for performance evaluations. The theoretical and experimental results were in agreement for free motion, and the deviation from the constrained motion was in the range of the experimental errors. The outcomes also provided an insight into the importance of considering lengthening for the soft actuators.

## 1 Introduction

Every year, thirteen million people globally suffer a stroke. Stroke, as the leading cause of long-term disability in the human upper extremities (80% of post-stroke individuals), causes adverse impacts on patients’ quality of life (Feigin et al., 2016). Rehabilitation and assistive robots have been designed and studied for performing physical and occupational therapy interventions with intensive/repetitive movements, such as continuous passive motion (CPM), active resistive motion (ARM), and assist-as-needed motion that facilitates restoring functionalities in the impaired hand of post-stroke individuals (Dobkin, 2004). Particularly, wearable robots for upper-body rehabilitation and physical assistance have benefited from soft robotic approaches due to the intrinsic mechanical compliance, adaptability, and versatile deformations provided by these robots, which make them suitable for safe interaction with the human body (Haghshenas-Jaryani et al., 2016b; Walsh, 2018) while addressing limitations involved in conventionally rigid robots, such as complex mechanisms, heavy weight, safety issues, and cost (Polygerinos et al., 2017a; Polygerinos et al., 2015b; Rus and Tolley, 2015; Haghshenas-Jaryani et al., 2016a; Haghshenas-Jaryani et al., 2017b). Despite the development of more than 50 soft exoskeletons for hand rehabilitation over the past 15 years (Chu and Patterson, 2018; Shahid et al., 2018), these robots still have limited capabilities for interacting with the human hand due to their simple control schemes, mainly based on the kinematics of robots (Kadowaki et al., 2011; Haghshenas-Jaryani et al., 2019). Effective control of rehabilitation robots requires taking into account the distributed and multi-contact point physical human–robot interaction (pHRI) and users’ biomechanical variation (Kamper and Rymer, 2000; Kamper and Rymer, 2001; Haghshenas-Jaryani et al., 2020; Tang et al., 2021). The studies conducted by Esmatloo et al. (2019) and Esmatloo and Deshpande (2020) on rigid hand exoskeletons and the physical human–robot interaction involved in rigid robots have shown promise. However, the progress in this area has been impeded, mainly due to the complexity of the pHRI, the highly nonlinear nature of soft robot dynamics (Polygerinos et al., 2017a; Robertson, 2019), and lack of studies to develop these models.

There are four main categories of modeling approaches in soft robotics based on mathematical techniques: continuum mechanics, geometrical, discrete material, and surrogate models (Armanini et al., 2023). Continuum mechanics models (Coevoet et al., 2017; Mustaza et al., 2019) use infinite dimensional configuration spaces to consider the physical aspects of soft-body deformations. These are best suited for problems that involve bending deformations and are described as beams or combinations of beams without significant cross-section inflation. Geometrical models (Trivedi et al., 2008; Tawk et al., 2019) rely on assumptions about the shape of soft bodies under specific loads and are useful for kinematic control with explicit analytic maps between actuation and configuration spaces. Discrete material models (Goldberg et al., 2019; Huang et al., 2020) divide continuous bodies into finite material components and may struggle with modeling constitutive equations and distributed actuation. Surrogate models (Johnson et al., 2021; Zhang et al., 2022) use machine learning and neural network models to obtain system configurations through datasets and a learning process. This modeling approach uses neural network models and machine learning algorithms.

Despite the extensive work in modeling soft continuum and articulated arms (manipulators) (Armanini et al. 2023; Trivedi et al. 2008; Renda et al. 2016; Hyatt et al. 2019; Li et al. 2019; Bruder 2020), theoretical models for soft robotic exoskeletons, particularly those used in hand rehabilitation, are lacking (Polygerinos et al., 2017b). Some of the early works in developing dynamic models and control for pneumatic soft actuators have studied a fiber-reinforced continuous fluidic elastomer actuator (FSA) or soft pneumatic actuator (SPA) (Polygerinos et al., 2015c; Nikolov et al., 2016). A novel asymmetric bellow flexible actuator was analytically and numerically (FEM-based) modeled, designed, and prototyped, and finally, experimentally studied. The differential expansions at different zones of the cross-section of the actuator were taken into account with elastic behavior for the analytical modeling (Udupa et al., 2014). Connolly et al. (2017) developed an analytical model for modeling multi-segment soft actuators, where each segment undergoes a combination of primitive motions, such as axial and radial expansions, and twisting. In two similar works, analytical models were developed to predict the relationship between the input pressure and the bending angle in the free-space motion, as well as the contact force when the actuator tip interacts with an external rigid surface (Nikolov et al. 2016; Wang et al. 2016). In a very recent work, Joshi and Paik (2023) developed a sensor-less force and pose estimation method for soft actuators, including PneuNet, McKibben muscle, fiber-reinforced SPA, and SPA skin, under different boundary and load conditions. Physical interactions between an FSA and a 3D human finger model with passive hinges were experimentally studied using tactile sensors embedded between the soft finger and the human finger model (Polygerinos et al., 2015a). In another work considering a hybrid soft-and-rigid structure for the robotic finger, the physical interactions of a fabric-based pneumatic exoskeleton with a human hand/finger were studied through modeling and experimental validation (Yun et al., 2017). Tang et al. developed an online learning model which takes into account the interaction of two soft-segment robotic digits and a human finger model for an adaptive control strategy (Tang et al., 2021). A simple force analysis and interaction model was developed for a cybernetic finger for hand paralysis (Yoshikawa et al., 2019), a bionic soft robotic glove (Zhao et al., 2021) with a hybrid soft-and-rigid architecture, and a torque characterization for enfolded textile-based soft actuators (Nassour and Hamker, 2019). Extensive work has been carried out on a hybrid soft-and-rigid actuator-based hand rehabilitation robot (UTARI REHAB Glove), where the soft robotic finger was made of an elastomer (Haghshenas-Jaryani et al., 2016a; Haghshenas-Jaryani et al., 2017b; Haghshenas-Jaryani et al., 2020), including pHRI kinematic and modeling and characterization (Haghshenas-Jaryani et al., 2017a; Haghshenas-Jaryani and Wijesundara, 2018). The results of these models were utilized for a kinematic-based adaptive control of a bilateral rehabilitation robot (Haghshenas-Jaryani et al., 2019), a simulation-based quasi-static force and position control (Haghshenas-Jaryani, 2020). A single-input single-output (SISO) quasi-static model-based adaptive position control of the soft exo-digit and the human finger physical interaction was developed and experimentally verified for a step input and trajectory-tracking cases (Haghshenas-Jaryani, 2022). Lapresa et al. (2022) introduced an open-source toolbox for comprehensive hand kinematic analysis, clinical assessment, and postural synergy extraction. However, it relies on specialized equipment (optoelectronic motion capture system and markers), which may not be universally accessible, and contains the potential lack of comprehensive coverage for all clinical scenarios and hand variations. Tamantini et al. (2023) introduced a psychophysiological-aware control strategy for upper limb robot-aided orthopedic rehabilitation, allowing adaptable treatment, guiding patients’ movements, and adjusting control parameters based on patient performance and psychophysiological state. However, it involved a relatively small sample of eight orthopedic patients, which may limit the generalizability of the findings to a larger population.

Numerical and machine learning approaches have been used to model and study hyperelastic materials and soft actuators made of materials characterized as hyperelastics. Finite-element analysis of fiber-reinforced soft elastomers was carried out, where the outcomes show that hyperelastic materials modeled by the Ogden material model perform better than neo-Hookean material model (Buffinton et al. 2020). Shiva et al. (2019) developed a quasi-static model for real-time position and force estimation in a soft finger-like robotic appendage. They showed that the inertial effect could be neglected by assuming static equilibrium and slow transitions. In addition, they studied a range of material models, and the results indicated that the Hookean assumption is valid only for small strains. However, these models may not capture highly nonlinear material behavior as accurately due to the limitation associated with the Ogden material model and the Hookean assumption. On the other hand, a new method for fast physics-based simulation of hyperelastic materials was proposed by Liu et al. (2016) and Li et al. (2019) who used a quasi-Newton method. A collision-aware technique based on geometric optimization was developed by incorporation of hyperelastic materials (Fang et al., 2022). The computational cost of these methods is low, unlike FEM-based simulations. Meng et al. (2021) developed a human–machine coupling model for the hybrid exoskeleton by taking into account the kinematics properties of human fingers and applying the Bernoulli beam equation, but the usage of the Bernoulli beam equation may simplify the modeling.

This paper presents a full analytical model of the physical interaction between a soft robotic exo-digit and an anthropomorphic finger model while applying quasi-static motion. The slow motion required for the hand physical therapies and a low inertial effect of the anthropomorphic finger and the soft robot justify the quasi-static assumption. An intertwining of kinematics and quasi-static motion was studied to model the distributed (multiple contact points) interaction between the robot and a human finger model. The human finger was modeled as an articulated multi-rigid body structure. The soft robot was modeled as an articulated hybrid soft-and-rigid model. A hyperelastic constitute model based on Yeoh’s 3rd-order material model was used for modeling the soft elastomer. The developed models were experimentally evaluated for 1) free motion of individual soft actuators and 2) constrained motion of the soft robotic exo-digit and the human finger model. Simulation and experimental results were compared for performance evaluations. Contributions to this work are listed as follows:

• Fully analytical modeling formulation of multi-contact point physical interaction between the anthropomorphic finger and a soft robotic exo-digit
• Experimental studies of the soft exo-digit actuation module in free motion and constrained motion to evaluate the analytical formulation;
• Experimental contact force measurements of physical human–robot interaction
• Simulation studies of free motion and constrained motion

The structure of this paper is set as follows: a quasi-static analytical model is derived in Section 2.1 with kinematics formulations of continuum bending of the soft actuator. Material constitute equations were derived based on hyperelastic properties. Section 2.2 describes multibody dynamics of the human finger, kinematics, and quasi-statics equations for human finger motion. Section 2.3 describes the coupling of the quasi-static models of the anthropomorphic finger and the soft robotic exo-digit into a single model for the simulation studies. Section 2.4 explains the procedure for fabricating soft exo-digit actuators, the setup for free and constrained motion, and the setup for measuring contact forces. Finally, Section 3 discusses the results obtained from simulations and experimental testing for two cases of free motion and constrained motion, which validated the presented quasi-static model of human–robot interaction.

## 2 Materials and methods

The main contribution is the modeling of multi-contact point physical interaction between the soft robotic exo-digit and an anthropomorphic model of finger. Modeling is described in the following sections according to 1) modeling of the soft exo-digit; 2) modeling a hyperelastic material constitutive relation, which will be embedded into soft robotic quasi-static equations; 3) modeling the anthropomorphic finger with multi-contact points; and finally, 4) modeling the coupled quasi-static formula of multi-contact point physical human–robot interaction.

### 2.1 Soft robotics exo-digit

#### 2.1.1 Kinematics of a single soft segment

**FIGURE 1:**
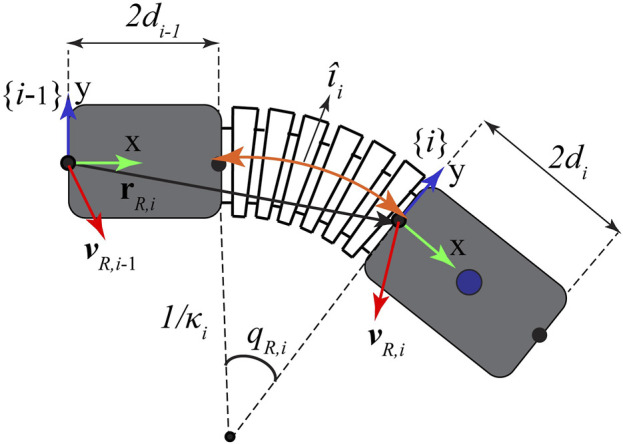
Kinematics of a section of the soft robotic digit, including bodies {*i* −1} and {*i*}.

Kinematics of the soft robotic exo-digit was derived by considering it an articulated soft robotic arm composed of three soft continuous joints (pseudo-joint) and between silicone-based semi-rigid bodies. A single soft actuation segment (pseudo-joint) is shown in Figure 1, where body {*i* − 1} is connected to body {*i*}. The vector describing the position of the origin of the semi-rigid linking bodies *ith*, *i* = 1, 2, 3 is given by

rii−1t=2di−1+xℓ^i,t,yℓ^i,t,0T, (1)

where 2*d*_*i*−1_ is the length of the semi-rigid block and $x\left({\hat{\ell }}_{i},t\right)$ and $y\left({\hat{\ell }}_{i},t\right)$ are given as

$$x\left({\hat{\ell }}_{i},t\right)=\frac{{\hat{\ell }}_{i}}{q_{i}}\sin\left(q_{R,i}\right)$$

$$y\left({\hat{\ell }}_{i},t\right)=\frac{{\hat{\ell }}_{i}}{q_{i}}\left(1-\cos\left(q_{R,i}\right)\right),$$

where ${\hat{\ell }}_{i}$ is the arc length of each soft segment. Assuming a constant length (arc length) ${\hat{\ell }}_{i}$ and constant curvature *κ*_*i*_ along the arc length of each soft pseudo-joint, the pseudo-joint angular motion can be obtained as follows:

$$q_{R,i}=\kappa_{i}\ell_{i}.$$ (2)

Accordingly, the orientation of the soft-bodied segment is described by a rotation matrix Rii−1(ηi,t)∈SO(3) , which represents the orientation of a body frame along the arc length of the backbone curve at *η*_*i*_ (i.e., frame {*i*}) and with respect to the base frame {*i* − 1}, as shown in Figure 1. For the planar bending, this rotation matrix has the following format $\eta_{i}={\hat{\ell }}_{i}$ for the attached body frame:

Rii−1=cosqR,i−sinqR,i0sinqR,icosqR,i0001, (3)

where *q*_*R*,*i*_ is given in Eq. 2. The position of each semi-rigid link, {*i*}, with respect to the reference frame can be expressed in a recursive form with respect to the previous link position (*i* = {1, 2, 3} and r00=0 ).

ri0=ri−10+Ri−10rii−1=ri−10+2di−1cφi−1−ℓ^iqR,isφi−1+ℓ^iqR,isφi2di−1sφi−1+ℓ^iqR,icφi−1+ℓ^iqR,icφi0. (4)

Now, differentiating Eqs 3, 4, the linear and angular velocities of the connecting link (body frame {*i*}) expressed in the reference frame are obtained as follows:

υR,i=υR,i−1+ωR,i−1×Ri−10rii−1+Ri−10di−1ridt=υR,i−1+ℓ^iqR,icφi−cφi−1q˙R,i−1+ℓ^iqR,i2qR,icφi−sφi+sφi−1q˙R,i−di−1sφi−1φ˙i−1ℓ^iqR,isφi−sφi−1q˙R,i−1+ℓ^iqR,i2qR,isφi+cφi−cφi−1q˙R,i+di−1cφi−1φ˙i−10, (5)

where $\varphi_{i}=\sum_{k=1}^{i}q_{R,k}$ , ${\dot{\varphi }}_{i}=\sum_{k=1}^{i}{\dot{q}}_{R,k}$ , and ${\dot{\varphi }}_{0}=\varphi_{0}=0$ .

ωR,i−1×=∂Ri−10∂tRi−10T=φ˙i0−10100000, (6)

and now, using Eq. 5 and Eq. 6, the velocity of the center of mass of body {*i*} can be obtained as

υR,iC=υR,i+ωR,i×Ri0riCi=υR,i+−disφiφ˙idicφiφ˙i0, (7)

where [ ]_×_ is the linear operator for converting a 3D vector to a skew-symmetric matrix (i.e., $R^{3}\to so\left(3\right)$ ), $\omega_{R,i}={\left[0,0,\omega_{Rz,i}\right]}^{T}$ , and riCi=[di,0,0]T . It should be noted that ***ω***_*R*,0_ = **0**, ***υ***_*R*,0_ = **0**, and R00=I3 , where $I_{3}\in R^{3\times 3}$ is the identity matrix. Moreover, the bending curvature, *κ*_*i*_, can be obtained by differentiating the rotation matrix Rii−1 with respect to the arc length variable *η*_*i*_ as follows:

$${\left[u_{R,i}\right]}_{\times }=R^{T}\frac{\partial R}{\partial \eta_{i}}{|}_{\eta_{i}={\hat{\ell }}_{i}},$$ (8)

where $u_{R,i}={\left[0,0,\kappa_{i}\right]}^{T}$ . Additionally, the Jacobian matrices, *J*_*R*,*i*_, which map the pseudo-joint velocities of soft robotic sections ${\dot{q}}_{R}$ to the linear $\upsilon_{R,i}^{C}$ and angular ***ω***_*R*,*i*_ velocities of the semi-rigid bodies can be written for a planar motion based on (5)–(6).

$${\hat{\upsilon }}_{R,i}={\left[v_{Ry,i}^{C}\;v_{Rx,i}^{C}\;\omega_{Rz,i}\right]}^{T}=J_{R,i}{\dot{q}}_{R}.$$ (9)

The full mathematical form of the Jacobian matrix for each soft robotic section is given in Supplementary Material.

#### 2.1.2 Equations of quasi-motion for a single soft segment

**FIGURE 2:**
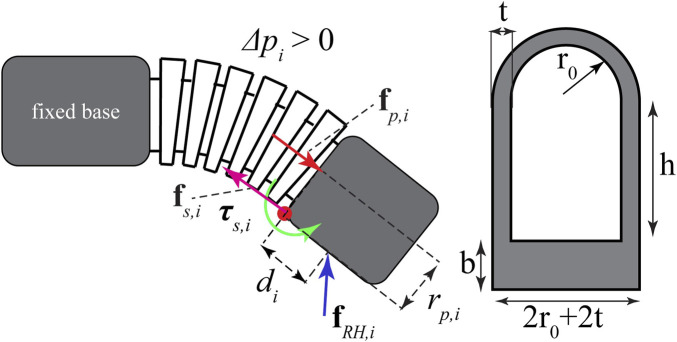
Forces and moments acting on the soft exo-digit during its motion and the cross-section of the half-bellow-shaped soft segment.

Quasi-statics of the soft continuous joints, which describes the balance of acting moments as a function of the actuation pressure *p*, the pseudo-joint angles of the soft robotic digit $q_{R}={\left[q_{R,1},q_{R,2},q_{R,3}\right]}^{T}$ , and the external force/torque is presented here. For the soft robotic exo-digit shown in Figure 2, the net torque, ***τ***_*i*_, generated by these half-bellow-shaped hollow structures is the resultant of three terms: 1) the pressure actuation torque $\tau_{p,i}={\left[0,0,\tau_{p,i}\right]}^{T}$ ; 2) the structural resistance torque $\tau_{s,i}={\left[0,0,\tau_{s,i}\right]}^{T}$ ; and 3) the external torque ***τ***_*e*,*i*_, given as follows:

$$\tau_{i}=\tau_{s,i}+\tau_{p,i}+\tau_{e,i},$$ (10)

where

$$\tau_{p,i}=r_{pi}A_{pi}p_{i},$$ (11)

while for the specific geometry of the soft-segment cross-section, shown in Figure 2, we have

$$\begin{array}{ll} r_{pi}A_{pi} & =\frac{\pi }{2}{\left(r_{0,i}+t_{i}\right)}^{2}\left(\frac{4\left(r_{0,i}+t_{i}\right)}{3\pi }+h_{i}+b_{i}\right)\\ & \;+2\left(r_{0,i}+t_{i}\right)\left(\frac{h_{i}}{2}+b_{i}\right)h_{i}, \end{array}$$ (12)

$$\tau_{s,i}=-\int_{A_{i}}\sigma_{i}\;r_{s,i}\;dA_{s,i}\;i=1,2,3,$$ (13)

and the external torque is defined as

$$\tau_{e,i}=-J_{R,i}^{T}f_{HR,i}\;i=1,2,3.$$ (14)

In Eq. 13, *σ*_*i*_ is the normal Cauchy stress at the cross-section of the soft actuator determined based on a *hyperelastic constitute model* (Haghshenas-Jaryani et al., 2017a; Haghshenas-Jaryani and Wijesundara, 2018; Haghshenas-Jaryani, 2020), which is discussed in Section 2.1.3; *r*_*si*_ is the moment arm distance; and *A*_*si*_ is the cross-sectional area for the soft continuous joint section; therefore, *r*_*s*,*i*_ d*A*_*s*,*i*_ is the first moment of area corresponding to the shaded part of the cross-section of the soft segment, as shown in Figure 2. It should be noted that Eq. 13 does not have an analytical solution due to the complex nonlinear form of the integrand, so it will be determined numerically (Haghshenas-Jaryani et al., 2017a; Haghshenas-Jaryani and Wijesundara, 2018; Haghshenas-Jaryani, 2020). Applying ***τ***_*i*_ = **0** to (10) yields a quasi-static equation for a single soft segment.

#### 2.1.3 Hyperelastic constitute model

The Cauchy stress, *σ*_*i*_, in Eq. 13, can be obtained by (Holzapfel, 2000)

$$\sigma_{i}=\lambda_{i}\frac{\partial W}{\partial \lambda_{i}}-\mu \;i=1,2,3,$$ (15)

where *W* and *μ* are the strain energy function and indeterminate Lagrange multiplier. The principal strain invariants, *I*_1_, *I*_2_, and *I*_3_, are defined as follows:

$$I_{1}=\lambda_{1}^{2}+\lambda_{2}^{2}+\lambda_{3}^{2},$$ (16)

$$I_{2}=\lambda_{1}^{-2}+\lambda_{2}^{-2}+\lambda_{3}^{-2},$$ (17)

$$I_{3}=\lambda_{1}\lambda_{2}\lambda_{3},$$ (18)

where *λ*_*i*_, *i* = {1, 2, 3} are the principal stretches in the axial, circumferential, and radial directions, respectively. Assuming no deformation in the circumferential direction yields *λ*_2_ = 1, based on the studies by Polygerinos et al. (2015c) and Nikolov et al. (2016). Additionally, incompressible properties of polymeric materials result in *λ*_1_ *λ*_2_ *λ*_3_ = 1. If we consider the major stretch in the axial direction as *λ*_1_ = *λ*, then solving Eq. 18 yields

$$\lambda_{3}=\frac{1}{\lambda }.$$ (19)

Due to the thin-wall feature of the soft section structure, the stress in the radial direction can be assumed to be zero, *σ*_3_ = 0; therefore, solving for *μ* from Eq. 15 and Eq. 19 yields

$$\mu =\frac{2}{\lambda^{2}}\frac{\partial W}{\partial I_{1}},$$ (20)

where the first principal invariant *I*_1_ is given by Eq. 16. As stated by Polygerinos et al. (2015c), Nikolov et al. (2016), and Haghshenas-Jaryani et al. (2017a), the axial stress, *σ*_1_, is dominant over the circumferential one, *σ*_2_, especially for the range of stretch 1 ≤ *λ* ≤ 1.5. Therefore, the axial stress is used here to calculate the structural resistance torque in Eq. 13. Substituting Eq. 20 into Eq. 15 and solving for the axial stress components, *σ*_1_, yields

$$\sigma_{1}=2\left(\lambda^{2}-\frac{1}{\lambda^{2}}\right)\frac{\partial W}{\partial I_{1}}.$$ (21)

Yeoh’s 3rd-order model (Holzapfel, 2000) was used to define the strain–energy function, *W*, with the following form:

$$W=C_{1}\left(I_{1}-3\right)+C_{2}{\left(I_{1}-3\right)}^{2}+C_{3}{\left(I_{1}-3\right)}^{3},$$ (22)

where *C*_1_, *C*_2_, and *C*_3_ are the coefficients of Yeoh’s 3rd-order model, which were determined experimentally using a standard material testing (Haghshenas-Jaryani et al. 2015; Haghshenas-Jaryani et al., 2017a). An experimental study of the elastomer showed a better fit to this hyperelastic modeling approach. The axial stress, *σ*_1_, can be derived by substituting (18) and (22) into (21).

$$\sigma_{1}\left(\lambda \right)=2\left(\lambda^{2}-\frac{1}{\lambda^{2}}\right)\left(C_{1}+\;C_{2}\tilde{\lambda }+\;C_{3}{\tilde{\lambda }}^{2}\right),$$ (23)

where

$$\tilde{\lambda }=\lambda^{2}+\frac{1}{\lambda^{2}}-2.$$

To calculate the resistant torque, *τ*_*si*_, in Eq. 13, the cross-section of the soft actuator (Figure 2) is divided into three zones: 1) a base with stress *σ*_*i*,*b*_; 2) the side straight walls with stress *σ*_*i*,*h*_; and finally, 3) the top arch section with *σ*_*i*,*t*_. The stretch for each zone is defined, respectively, as follows:

$$\lambda_{b,i}=1+\frac{y\;q_{R,i}}{{\hat{\ell }}_{i}}\;\;\;0<y<b,$$ (24)

$$\lambda_{h,i}=1+\frac{y\;q_{R,i}}{{\hat{\ell }}_{i}}\;\;\;b<y<b+h,$$ (25)

$$\lambda_{t,i}=1+\frac{\left(b+h+r\sin\left(\phi \right)\right)\;q_{R,i}}{{\hat{\ell }}_{i}}\;r_{0}<r<r_{0}+t.$$ (26)

The resistant torque is calculated by substituting (24)–(26) into (23) for each zone and adding them up in Eq. 13 as follows:

$$\begin{array}{ll} \tau_{si}\left(q_{R,i}\right) & =\int_{0}^{b_{i}}2\sigma_{i,b}\left(r_{0,i}+t_{i}\right)ydy+\int_{b_{i}}^{h_{i}+b_{i}}2\sigma_{i,h}t_{i}\;ydy\\ & \;+\int_{r_{,i}}^{r_{0,i}+t_{i}}\int_{0}^{\pi /2}2\sigma_{i,t}r\left(b_{i}+h_{i}+r\sin\left(\phi \right)\right)d\phi dr. \end{array}$$ (27)

It should be noted that for the last term in Eq. 27, we used a Taylor series expansion with three terms with respect to *ϕ* of approximately 0 to obtain the closed-form solution. The integration yields the following closed-form solution for the joint {*i*}:

$$\tau_{si}\left(q_{R,i}\right)=\frac{\sum_{k=0}^{13}a_{b,k}q_{R,i}^{k}}{\sum_{j=2}^{7}b_{b,j}q_{R,i}^{j}}+\frac{\sum_{k=0}^{18}a_{h,k}q_{R,i}^{k}}{\sum_{j=2}^{12}b_{h,j}q_{R,i}^{j}}+\frac{a_{t,1}q_{R,i}}{\sum_{j=0}^{8}b_{t,j}q_{R,i}^{j}}.$$ (28)

The coefficients of the polynomials of the rational functions in Eq. 28 are given in Supplementary Material.

#### 2.1.4 Multiple soft-bodied quasi-static equations

**FIGURE 3:**
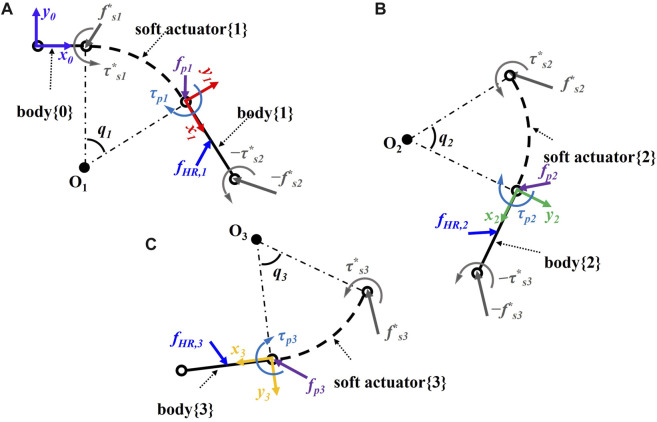
Schematic of the soft robotic digit interacting with a human finger including bodies {*i* − 1} and {*i*} and soft actuator {*i*} for **(A)** MCP, **(B)** PIP, and **(C)** DIP joint.

The balance of forces and moments for the body {i} about its proximal point and the soft actuator {i} about its proximal point (for *i* = 1,2,3 and fs(4)*3=3τs(4)*=0 ), as shown in Figures 3A–C, is given as follows:

fHR,ii+fpii−fsi+1*i+fsii=0, (29)

τsii−τsi+1*i+τpii−rii+1i×fsi+1*i+rcii×fHR,ii=0, (30)

where

fpii=piApi,0,0Ti=1,2,3,

fHR,ii=0,fi,0Ti=1,2,3,

τsj*i=Rjiτsjj+r^iji×Rjifsjj,

fsj*i=Rjifsjj,

r^iji=ℓ^jqjsj,ℓ^jqj1−cj,0Tj=1,2,3i=j−1,

rcii=di,0,0T,

and r^ijj is the position vector connecting the proximal and distal points of the arc of the soft actuator {*j*}; rcii is the position vector describing the center of mass of the body {*i*} with respect to the body frame.

Adding up all three soft segment equations (Eqs 29, 30) yields

$$\tau \left(q_{R}\right)+B_{R}p-\overset{3}{\underset{i=1}{\sum }}J_{R,i}^{T}f_{HR,i}=0,$$ (31)

where $\tau \left(q_{R}\right)={\left[\tau_{s1}\left(q_{R,1}\right),\tau_{s2}\left(q_{R,2}\right),\tau_{s3}\left(q_{R,3}\right)\right]}^{T}$ is the vector of nonlinear functions designated to represent the non-analytical form of the resistive structural torques *τ*_*si*_ in Eq. 13 as a function of pseudo-joint variables **q**_*R*_; **f**_*HR*,*i*_ = −**f**_*RH*,*i*_ is the vector of human–robot interaction forces; *J*_*R*,*i*_ are the Jacobian matrices for each soft robotic section (provided in Supplementary Material); $p={\left[p_{1},p_{2},p_{3}\right]}^{T}$ is the vector of actuation pressures (i.e., control inputs); and *B*_*R*_ is a diagonal matrix of the first moment of the cross-sectional area of each soft segment, where the internal pressure actuation *p*_*i*_ acts on them:

$$B_{R}=\left[\begin{array}{ccc} r_{p1}A_{p1} & 0 & 0\\ 0 & r_{p2}A_{p2} & 0\\ 0 & 0 & r_{p1}A_{p3} \end{array}\right].$$

The detailed form of Eqs 29, 30 is given in Supplementary Material.

### 2.2 Anthropomorphic finger model

**FIGURE 4:**
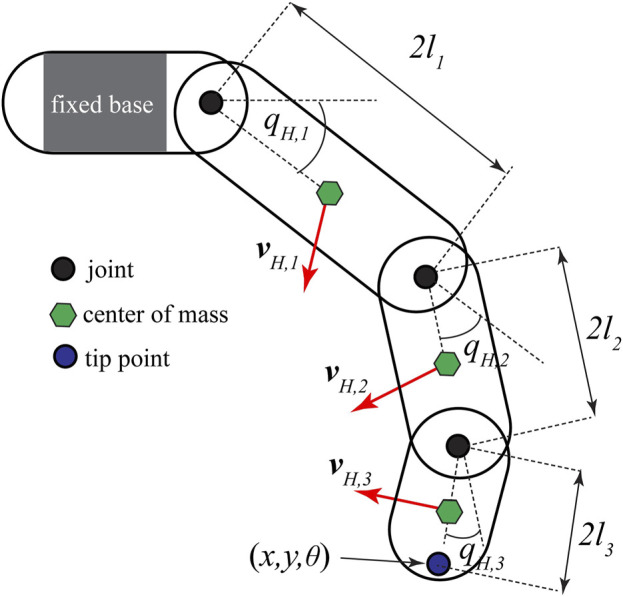
Anthropomorphic model of a finger.

The anthropomorphic finger, a simplified model of a human finger, was modeled as a serial kinematic chain of three moving rigid links and a fixed base link with a single degree-of-freedom hinge joint connecting them together, as shown in Figure 4. The motion of the joints is described by $q_{H}={\left[q_{H,1},q_{H,2},q_{H,3}\right]}^{T}$ . The equations of motion for the human finger model can be derived in the form of general rigid multibody dynamics as

$$\underset{=\text{ 0}}{\underbrace{M\left(q_{H}\right){\ddot{q}}_{H}+c\left(q_{H},{\dot{q}}_{H}\right)}}+K_{H}q_{H}=\overset{3}{\underset{i=1}{\sum }}J_{H,i}^{T}f_{RH,i},$$ (32)

where the passive torque at the human joints was modeled as torsional springs ***τ***_*H*_ = *K*_*H*_ **q**_**H**_ and the external torques due to the contact forces, **f**_*RH*,*i*_, between the soft robot and the human finger are included in Eq. 32. It is assumed that the contact forces due to the multi-contact point physical interaction between the soft robot and the human finger model will be applied at the center of mass of each link, where *M* and **c** are the mass matrix and the vector of nonlinear terms, respectively. *K*_*H*_ and **f**_*RH*,*i*_ are the joint torsional stiffness matrix and the contact force vectors, respectively.

$$K_{H}=\left[\begin{array}{ccc} k_{H,1} & 0 & 0\\ 0 & k_{H,2} & 0\\ 0 & 0 & k_{H,3} \end{array}\right].$$

In Eq. 32, *J*_*H*,*i*_ are the Jacobian matrices that map the joint velocities ${\dot{q}}_{H}$ to the linear ***υ***_*H*,*i*_ and angular ***ω***_*H*,*i*_ velocities of each link, as shown in Figure 4, and can be obtained as

$${\hat{\upsilon }}_{H,i}={\left[v_{Hy,i}\;v_{Hx,i}\;\omega_{Hz,i}\right]}^{T}=J_{H,i}{\dot{q}}_{H},$$ (33)

where ${\hat{\upsilon }}_{H,i}$ is a vector that combines the planar linear and angular velocity components. The full form of the Jacobian matrix for the center of mass of each link is given in Supplementary Material.

The time-dependent terms shown in Eq. 32 were eliminated due to the quasi-static assumption, which was validated by Haghshenas-Jaryani et al. (2017a) and Haghshenas-Jaryani and Wijesundara (2018) using finite-element modeling and experimental testing.

### 2.3 Coupled quasi-static formulation of pHRI

After developing the quasi-static models of the human finger and the soft robotic exo-digit separately, these models were combined to derive a coupled model for pHRI, which will be used for control algorithm development. For simplifying the form of equations, the contact forces were written as fRH,i=Ri0fRH,ii and fHR,i=Ri0fHR,ii , where fRH,ii=−fHR,ii=[0,fi,0]T are the contact forces expressed in the body frame {*i*}. By introducing special human–robot contact force vectors $f_{RH}^{*}=-f_{HR}^{*}={\left[f_{1},f_{2},f_{3}\right]}^{T}$ , the summation terms in Eq. 32 and Eq. 31 can be written in the following compact form, where the quasi-static equations for both the human finger model and the soft robotic exo-digit are defined as follows:

$$K_{H}q_{H}-{\left(J_{H}^{*}\right)}^{T}f_{RH}^{*}=0,$$ (34)

$$\tau \left(q_{R}\right)+B_{R}p-{\left(J_{R}^{*}\right)}^{T}f_{HR}^{*}=0,$$ (35)

where Jacobian matrices are given as

$${\left(J_{H}^{*}\right)}^{T}=\left[\begin{array}{ccc} \ell_{1} & \ell_{2}+2\ell_{1}c_{2} & \ell_{3}+2\ell_{2}c_{3}+2\ell_{1}c_{23}\\ 0 & \ell_{2} & \ell_{3}+2\ell_{2}c_{3}\\ 0 & 0 & \ell_{3}, \end{array}\right]$$

$${\left(J_{R}^{*}\right)}^{T}=\left[\begin{array}{ccc} \frac{{\hat{\ell }}_{1}}{q_{1}^{2}}\left(1-c_{1}\right)+d_{1} & d_{2}+2d_{1}c_{2}+\zeta_{6}\;c_{12}+\zeta_{7}\;s_{12} & d_{3}+2d_{2}c_{3}+2d_{1}c_{23}+\zeta_{8}\;c_{123}+\zeta_{9}\;s_{123}\\ 0 & \frac{{\hat{\ell }}_{2}}{q_{2}^{2}}\left(1-c_{2}\right)+d_{2} & \frac{{\hat{\ell }}_{3}}{q_{3}}s_{3}-\frac{{\hat{\ell }}_{2}}{q_{2}^{2}}\left(c_{23}-c_{3}+s_{3}q_{2}\right)+2d_{2}c_{3}+d_{3}\\ 0 & 0 & \frac{{\hat{\ell }}_{3}}{q_{3}^{2}}\left(1-c_{3}\right)+d_{3} \end{array}\right]$$

and

$$\zeta_{1}=\frac{{\hat{\ell }}_{1}\left(c_{1}+q_{1}s_{1}-1\right)}{q_{1}^{2}}\;\zeta_{2}=\frac{{\hat{\ell }}_{2}\left(s_{1}-q_{1}c_{1}\right)}{q_{1}^{2}}\;\zeta_{3}=\frac{{\hat{\ell }}_{2}\left(c_{12}-c_{1}\right)}{q_{2}},$$

$$\zeta_{4}=\frac{{\hat{\ell }}_{3}\left(s_{123}-s_{12}\right)}{q_{3}}\;\zeta_{5}=\frac{{\hat{\ell }}_{3}\left(c_{123}-c_{12}\right)}{q_{3}},$$

$$\zeta_{6}=\zeta_{1}+\zeta_{3}\;\zeta_{7}=\zeta_{1}-\zeta_{3}\;\zeta_{8}=\zeta_{4}+\zeta_{1}+\zeta_{3}\;\zeta_{9}=-\zeta_{5}+\zeta_{2}-\zeta_{3}.$$

Both upper triangular special Jacobian matrices ${\left(J_{H}^{*}\right)}^{T}$ and ${\left(J_{R}^{*}\right)}^{T}$ are invertible by assuming **q** ≠ **0**. Eliminating the force vectors between Eq. 34 and Eq. 35 with **q**_*R*_ = **q**_*H*_ = **q** yields

$$\tau \left(q\right)+B_{R}p-{\left(J_{R}^{*}\right)}^{T}{\left(J_{H}^{*}\right)}^{-T}K_{H}q=0,$$ (36)

which represents the coupled quasi-statics of the human–robot interaction. The coupled model (36) can be used for the design of model-based control laws for the physical interaction between the soft robotic exoskeleton and human finger to follow the desired trajectories (Alam et al., 2023).

### 2.4 Experimental setup and procedures

The experimental part involves pneumatically pressurizing the soft exo-digit modules and tracking their elongation with or without a human finger model. The exo-digit modules are fabricated in the laboratory. The following subsections explain the fabrication process as well as the experimental parts.

#### 2.4.1 Fabrication of the silicone digit

**FIGURE 5:**
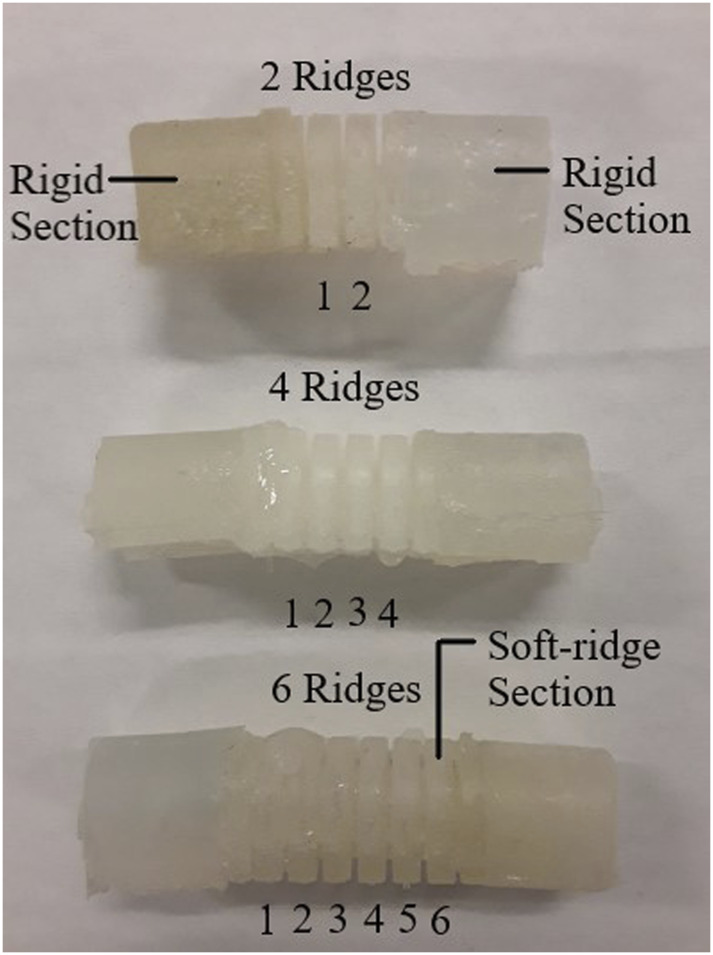
Silicon exo-digit modules.

A silicone exo-digit module consists of a soft-ridge section (a hollow half-bellow corrugated structure) between two semi-rigid blocks. The parts are made of an RTV silicone rubber (RTV-4234-T4, Xiameter, Dow Corning). The module name is based on the number of ridges in the soft section. The rigid and soft sections of the exo-digit module are shown in Figure 5, along with the three types of modules employed in this experiment, namely, the two-ridge, four-ridge, and six-ridge, corresponding to the DIP, PIP, and MCP joints of the human finger, respectively. The fabrication of the soft section involves three steps: first, preparing a wax mold for the interior of the soft section; second, inserting this wax mold inside the larger mold which will be filled with silicone; and third, melting out the wax from the interior of the soft section to leave a cavity in the soft section piece. Direct silicone injection into the rigid section mold is used to create the rigid portion. There are two ways to merge the soft and rigid sections: the weight approach and the mold method. The soft and rigid sections are constructed separately in the weight method and then merged by sandwiching some silicone in the middle and using weight to push the pieces together. On the other hand, the mold approach needs two sizable binder clips and a finger attachment mold. The finger attachment mold must contain the manufactured soft and rigid segments with a thin layer of silicone positioned between them. They are held together in the mold using binder clips. Overnight or in a laboratory oven at 60°C for 30 min, the soft and rigid parts solidify.

#### 2.4.2 Free motion

**FIGURE 6:**
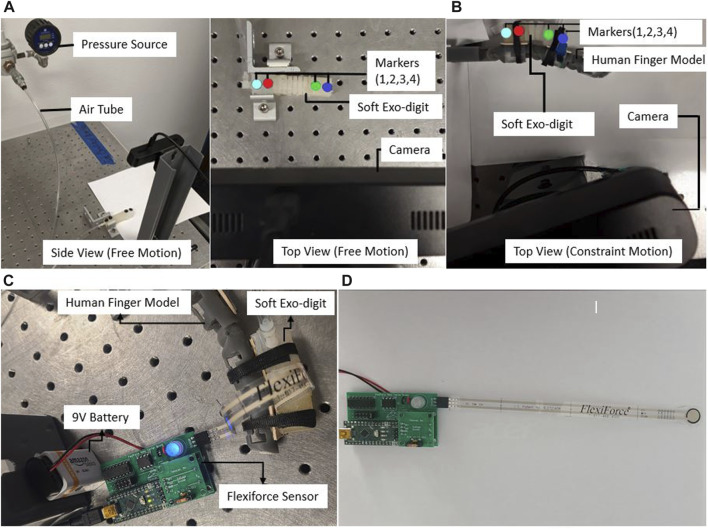
Experimental setup for **(A)** free motion, **(B)** constrained motion, and **(C)** force measurement: the force sensor is linked to a 9-V battery and a USB cable for the purpose of transmitting force readings to a PC **(D)** Tekscan FlexiForce A201-25 sensor.

Free bending motion of the exo-digit module is examined, while the actuation pressure is applied in increments of 10 kPa. For the two-ridge, four-ridge, and six-ridge modules, the range of pressure is 0–50 kPa, 0–30 kPa, and 0–50 kPa, respectively. The motion of the attached markers is recorded using the camera. Figure 6A displays the experimental setup for the free-motion test. With this configuration, every time the actuation pressure is increased, the camera takes a picture of the module with the connected markers. Two samples each of two-ridge, four-ridge, and six-ridge modules are tested. A MATLAB program is built using the Image Processing Toolbox to detect the markers and determine the location of each marker from the acquired images. With the increment of the actuation pressure, we can determine the amount of bending of the exo-digit modules based on the location of the marker.

#### 2.4.3 Constrained motion

To investigate how the soft robotic exo-digit interacts with the human model, a number of experiments are conducted on two-ridge, four-ridge, and six-ridge modules by attaching them to a human finger model. The human finger model, modular 3D-printed limbs connecting with revolute joints, is equipped with a torsional spring at each joint to represent the stiffness of the human joints. The applied pressure range in this case is 0–50 kPa, 0–30 kPa, and 0–50 kPa for the two-ridge, four-ridge, and six-ridge modules, respectively. The experimental setup for the constrained motion test is shown in Figure 6B. The exo-digit module is coupled to the human finger model (corresponding to the designated joint) in the constrained motion, which is the only distinction between the setup of the free- and constrained-motion tests. The positions of the markers are determined using the MATLAB calculation, just like the free-motion test.

**FIGURE 7:**
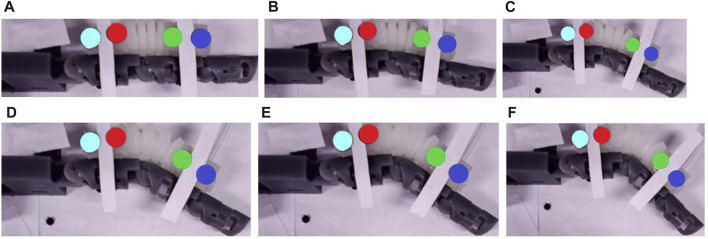
The images captured by the camera at **(A)** 0 kPa, **(B)** 10 kPa, **(C)** 20 kPa, **(D)** 30 kPa, **(E)** 40 kPa, and **(F)** 50 kPa applied pressure.

Figure 7 displays the images captured by the camera with the increment in the actuation pressure. To determine how the bending motion varies, each experiment is run twice for each type of module.

#### 2.4.4 Force experiment

A force experiment is carried out to determine the forces developed during the interaction of the soft exo-digit and the human finger model. The force measurement is performed using the Tekscan FlexiForce Prototyping Kit with the voltage divider analog circuit module and the Tekscan FlexiForce A201-25 sensor, which has a measuring range from 0 to 25 lb. The data collection is performed using the Tekscan open-source FlexiForceProtoData application. A pneumatic actuation pressure that ranges from 0 to 60 kPa with an increase of 10 kPa is supplied through a tube. During this time, the reading from the pressure sensor is gathered. A correlation between the actuation pressure and the produced force is found from the reading. Figure 6C displays the setup for the force experiment. The pressure sensor used in the experiment is shown in Figure 6D.

## 3 Results and discussion

### 3.1 Experimental results of the deformation test

**FIGURE 8:**
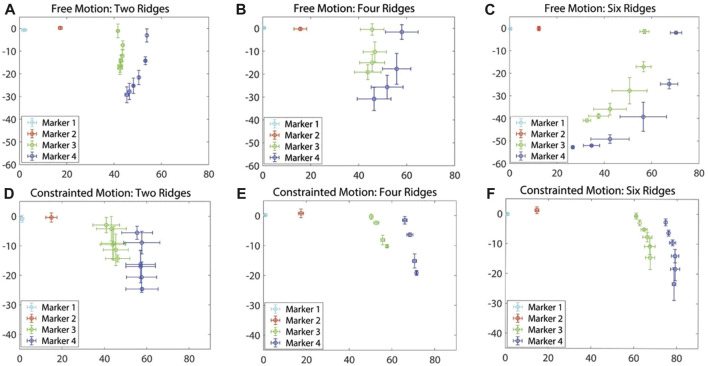
Mean and standard deviation for the free motion **(A–C)** and constrained motion **(D–F)**.

In the experiment, free-motion and constrained-motion scenarios are used to examine the deformation of the soft exo-digit due to the internal pressure change under these two conditions. The constrained motion occurs when the exo-digit is pressed against the human finger model, whereas the free motion does not use the human finger model. The mean and the standard deviation of the location of the markers during the movement are determined based on the results of two samples for each exo-digit module in both scenarios. The mean values and error bars (equivalent to one standard deviation in both *x-* and *y-*direction) of the free and constrained motions of each digit are shown in Figure 8. Since markers 1 and 2 are connected to the fixed link, there is small-to-no variation in their positions as a result of changes in the applied actuation pressure. Markers 3 and 4 are on the movable link, so we can observe changes in their positions as the actuation pressure varies. The mean and standard deviation of the position of a specific marker can be determined for a given pressure because two samples are collected for each module. The standard deviation of markers 3 and 4 is largely along the *y*-direction, as shown in Figures 8A, F, indicating that the exo-digit did not elongate equally in the *y*-direction. The variation between the results of two same-sized samples (two different same-sized exo-digits) could be related to the difference in the hand-made fabrication of the samples (slight wall thickness, or assembly of parts), error due to the image processing data extraction, and the slight difference between the assembly of the parts in the test setup. The error bars, equivalent to one standard deviation, of markers 3 and 4 are shown in both *x-* and *y-*direction in Figures 8B–E; however, good agreement can be observed between the marker positions in two samples with a small standard deviation. Table 1 reports the summary of the result given in Figure 8. Some of the rows in the table are empty because they do not have any significant standard deviation in two sets of data (Figure 8E). It can be concluded from the table that, for the free motion, the maximum or minimum standard deviation of elongation for both markers always corresponds to the same actuation pressure. We can also say that the six-ridge module elongates the most in response to applied pressure because it has the longest length. According to the constrained-motion results, the six-ridge module bends the most, while the second most bending happens to the four-ridge module. Unlike the free motion, the maximum or minimum standard deviation of elongation of the constrained motion in the *x-* and *y-*direction does not correspond to the same actuation pressure, as shown in Table 1. Finally, by comparing the results of free and constrained motion, we can conclude that the module does not bend as much for constrained motion as it does for free motion.

**TABLE 1:** Summarized result of Figure 8.

Figure	SD	Actuation pressure (Kpa)	Marker 3 (x) (mm)	Marker 3 (y) (mm)	Marker 4 (x) (mm)	Marker 4 (x) (mm)
Figure 8A	Maximum	50	1.06	2.75	0.83	3.5
	Minimum	10	0.48	1.91	0.3	1.72
Figure 8B	Maximum	30	5.54	3.3	7.03	5.05
	Minimum	10	4.88	4.4	5.88	6.7
Figure 8C	Maximum	20	7.4	5.8	9.8	6.45
	Minimum	50	1.6	0.31	0.65	0.59
Figure 8D	Maximum	10	16	9	18	9
	Minimum	20	12	-	16	-
		50	-	4	-	3
Figure 8E	Maximum	20	-	1.6	-	2.36
	Minimum	-	-	-	-	-
Figure 8F	Maximum	50	-	4.04	-	5.47
		40	4	-	4	-
	Minimum	0	-	4	-	4

### 3.2 Experimental result of the force experiment

**FIGURE 9:**
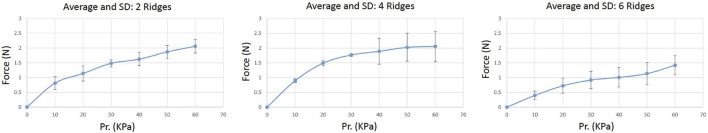
Mean and standard deviation for the force experiment result.

The force experiment was carried out three times for each joint, and the mean and standard deviation for each joint are given in Figure 9. The results show that the force between the finger model and exo-digit module increases with an increase in actuation pressure. Furthermore, Figures 9A–C demonstrate that the force measurements exhibit a greater standard deviation in the pressure range of 40–60 kPa. Additionally, the force growth rate decreases significantly after 40 kPa.

### 3.3 Theoretical model results of simulated deformation

**FIGURE 10:**
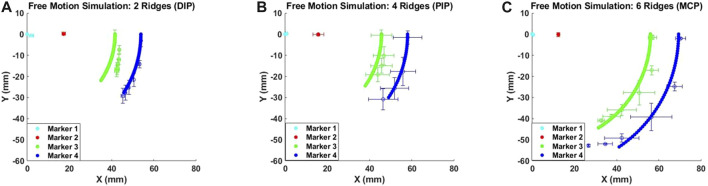
Simulated deformation results for the free motion of **(A)** DIP, **(B)** PIP, and **(C)** MCP joint.

**FIGURE 11:**
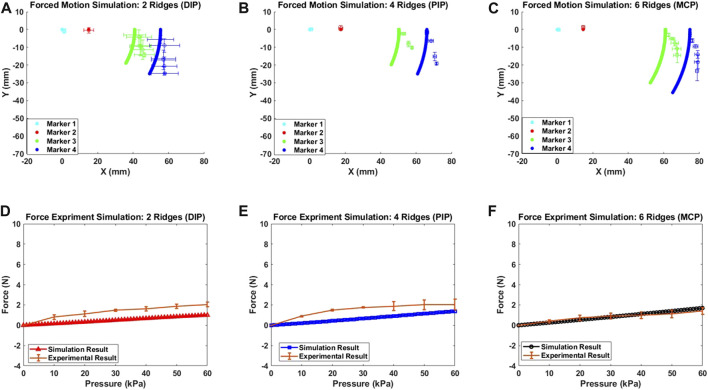
Simulated deformation results for the constrained motion **(A)**, **(B)**, and **(C)**; simulated force measurement results for each joint **(D)**, **(E)**, and **(F)**.

A MATLAB-based algorithm was developed to simulate the free motion of the soft exo-digit. Initially, the algorithm symbolically computed the pressure torque and structural resistance torque for the soft exo-digit. The free-motion condition assumed no external torque. A seventh-degree polynomial was fitted to the joint angle data obtained from the experiment, and the total torque was calculated symbolically. The real roots of the polynomial were considered the joint angles. Subsequently, the position of the four markers with the variation in the applied pressure was determined using these joint angles, the semi-rigid body and soft joint length, and the kinematics of the soft exo-digit, as given in Section 3. The simulation results, given in Figures 10A–C, demonstrated the free motion of the exo-digit and were compared to experimental data. The experimental data exhibited a good agreement with the simulation outcomes. To conduct a constrained motion simulation, the total torque is calculated by factoring in the external torque resulting from the interaction between the human finger model and the robotic exo-digit. The reaction force is computed using the approach presented by Haghshenas-Jaryani and Wijesundara (2018), with the assumption that only one joint functions at a time, and no other link impacts the force. Figures 11D–F display the outcome of the simulated force analysis for the DIP, PIP, and MCP, respectively, which was compared with the experimental results. The experimental results agreed with the simulation result quite well.

**FIGURE 12:**
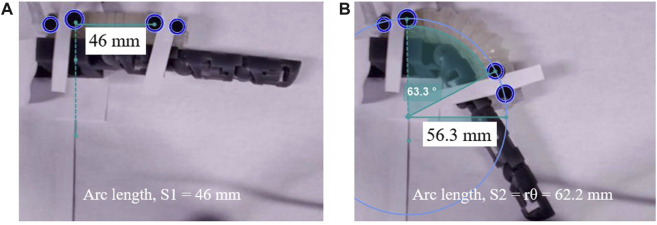
Soft exo-digit’s arc length variation with pressure increase during the constrained motion: **(A)** 46 mm length at no pressure, **(B)** 62.2 mm length at 50kPa applied pressure.

Once the total torque is calculated, the remaining code is identical to that of the free-motion simulation code. Figures 11A–C illustrate the outcome of the constrained-motion simulation for each joint. In practice, the length of the soft exo-digit increases when it bends under pressure, as shown in Figure 12. However, for the sake of simplicity, we assumed that the arc length remains constant. This phenomenon is particularly significant in constrained-motion simulations, as the elongation of the arc length in free motion facilitates bending. Conversely, constrained motion hinders this easy bending and leads to a notable elongation effect of the arc length.

The root mean square error (RMSE) between the simulation and actual results is given in Table 2. A higher RMSE value is observed for constrained motion than for free motion. This disparity can be attributed to the elongation of the soft exo-digit during motion, as well as the inclusion of the linear spring constant in the model. Table 3 reports the average standard deviation for both free- and constrained-motion experiments across all three joints, which helps explain the larger RMSE values given in Table 2.

**TABLE 2:** Root mean square error comparison between simulated and experimental results.

Motion →	Free motion: RMSE (mm)				Constrained motion: RMSE (mm)			
Joints *↓*	Marker 3 (x)	Marker 3 (y)	Marker 4 (x)	Marker 4 (y)	Marker 3 (x)	Marker 3 (y)	Marker 4 (x)	Marker 4 (y)
Two-ridge	4.33	2.65	11.70	6.87	5.96	3.03	14.10	3.97
Four-ridge	3.47	2.96	11.87	4.96	7.03	5.78	15.37	3.22
Six-ridge	2.55	5.65	12.11	10.93	8.79	9.92	13.35	7.56

**TABLE 3:** Average of the standard deviation of the free- and constrained-motion experiments.

Motion →	Free motion: (mm)				Constrained motion: (mm)			
Joints *↓*	Marker 3 (x)	Marker 3 (y)	Marker 4 (x)	Marker 4 (y)	Marker 3 (x)	Marker 3 (y)	Marker 4 (x)	Marker 4 (y)
Two-ridge	0.73	2.61	0.39	3.03	6.11	4.00	7.43	3.64
Four-ridge	5.14	3.43	6.60	5.03	0.83	0.92	0.91	1.25
Six-ridge	4.20	2.21	4.62	1.96	1.47	1.78	1.08	2.45

## 4 Conclusion

This work presents the development of a quasi-static analytical model for modeling distributed physical interaction between a human and a soft robotic exoskeleton for assisted hand motion. Quasi-static analytical models were developed for modeling the motion of a soft robot, the human finger, and their coupled physical interaction. An intertwining of kinematics and quasi-static motion was studied to model the distributed (multiple contact points) interaction between the robot and a human finger model. The human finger was modeled as an articulated multi-rigid body structure. The soft robot was modeled as an articulated hybrid soft-and-rigid model with a constant bending curvature and a constant length for each soft segment. A hyperelastic constitute model based on Yeoh’s 3rd-order material model was used for modeling the soft elastomer. The developed models were experimentally evaluated for 1) free motion of individual and fully integrated soft actuators and 2) constrained motion of the soft robotic exo-digit and the humanfinger model. Simulation and experimental results were compared for performance evaluations. The theoretical and experimental results were in agreement for free motion, and the deviation from the constrained motion is in the range of the experimental errors. The outcomes also provided an insight into the importance of considering lengthening for the soft actuators.

Future work should develop an analytical model in which the soft segment has a more realistic assumption of varying length. In addition, interaction of the soft exo-robot-augmented human hand with the environment (e.g., interaction with objects during grasping) will be modeled and experimentally validated.

## Data Availability

The original contributions presented in the study are included in the article/Supplementary Materials, further inquiries can be directed to the corresponding author.
