# Supplementary material for: Modeling multi-contact point physical interaction between the anthropomorphic finger and soft robotic exo-digit for wearable rehabilitation robotics applications
Source: Front Robot AI. 2023 Nov 17;10:1209609. doi: 10.3389/frobt.2023.1209609 (PMC10693461; doi:10.3389/frobt.2023.1209609)
Supplement: Supplementary file 1 [file DataSheet1.PDF]

Supplementary Material

Here, C1, C2, C3 are the coefficients of the Yeoh 3rd order model. b,L, h,r,r_0,t are the geometric parameters of the soft segment shown in Figure 2. d_1, d_2, and d_3, are the half-length of semi-rigid blocks in the soft-exo digit (see Figure. 1). ℓ_1, ℓ_2, and ℓ_3 are the half length of the rigid links in the anthropomorphic model (see Figure 4).

In (9),

$$\begin{aligned}
\hat{\boldsymbol{v}}_{R,i} &= [v_{Ry,i}\, v_{Rx,i}\, \omega_{Rz,i}]^T = J_{R,i}\dot{\mathbf{q}}_R \\
J_{R,1} &= \begin{bmatrix} h_{11} & 0 & 0 \\ h_{21} & 0 & 0 \\ 1 & 0 & 0 \end{bmatrix} \\
J_{R,2} &= \begin{bmatrix} a_{11} & a_{12} & 0 \\ a_{21} & a_{22} & 0 \\ 1 & 1 & 0 \end{bmatrix} \\
J_{R,3} &= \begin{bmatrix} b_{11} & b_{12} & b_{13} \\ b_{21} & b_{22} & b_{23} \\ 1 & 1 & 1 \end{bmatrix}
\end{aligned}$$

where

$$h_{11} = \frac{\hat{\ell}_1}{q_1^2}(q_1 c_1 - s_1) - d_1 s_1 \quad h_{21} = \frac{\hat{\ell}_1}{q_1^2}(q_1 s_1 + c_1 - 1) + d_1 c_1$$

$$a_{11} = h_{11} + \frac{\hat{\ell}_2}{q_2}(c_{12} - c_1) - d_1 s_1 - d_2 s_{12} \quad a_{21} = h_{21} + \frac{\hat{\ell}_2}{q_2}(s_{12} - s_1) + d_1 c_1 + d_2 c_{12}$$

$$a_{12} = \frac{\hat{\ell}_2}{q_2^2}(q_2 c_{12} - s_{12} + s_1) - d_2 s_{12} \quad a_{22} = \frac{\hat{\ell}_2}{q_2^2}(q_2 s_{12} + c_{12} - c_1) + d_2 c_{12}$$

$$b_{11} = a_{11} + \frac{\hat{\ell}_3}{q_3}(c_{123} - c_{12}) - d_2 s_{12} - d_3 s_{123} \quad b_{12} = a_{12} + \frac{\hat{\ell}_3}{q_3}(c_{123} - c_{12}) - d_2 s_{12} - d_3 s_{123}$$

$$b_{21} = a_{21} + \frac{\hat{\ell}_3}{q_3}(s_{123} - s_{12}) + d_2 c_{12} + d_3 c_{123} \quad b_{23} = \frac{\hat{\ell}_3}{q_3^2}(q_3 s_{123} + c_{123} - c_{12}) + d_3 c_{123}$$

$$b_{13} = \frac{\hat{\ell}_3}{q_3^2}(q_3 c_{123} - s_{123} + s_{12}) - d_3 s_{123} \quad b_{22} = a_{22} + \frac{\hat{\ell}_3}{q_3}(s_{123} - s_{12}) + d_2 c_{12} + d_3 c_{123}$$

For Fig.3(a):

$$ {}^{1}\mathbf{f}_{HR,1} + {}^{1}\mathbf{f}_{p1} - {}^{1}\mathbf{f}^{*}_{s2} + {}^{1}\mathbf{f}_{s1} = \mathbf{0} $$
$$ {}^{1}\boldsymbol{\tau}_{s1} - {}^{1}\boldsymbol{\tau}^{*}_{s2} + {}^{1}\boldsymbol{\tau}_{p1} - {}^{1}\mathbf{r}_{12} \times {}^{1}\mathbf{f}^{*}_{s2} + {}^{1}\mathbf{r}_{c1} \times {}^{1}\mathbf{f}_{HR,1} = \mathbf{0} $$

for Fig.3(b):

$$ {}^{2}\mathbf{f}_{HR,2} + {}^{2}\mathbf{f}_{p2} - {}^{2}\mathbf{f}^{*}_{s3} + {}^{2}\mathbf{f}_{s2} = \mathbf{0} $$
$$ {}^{2}\boldsymbol{\tau}_{s2} - {}^{2}\boldsymbol{\tau}^{*}_{s3} + {}^{2}\boldsymbol{\tau}_{p2} - {}^{2}\mathbf{r}_{23} \times {}^{2}\mathbf{f}^{*}_{s3} + {}^{2}\mathbf{r}_{c2} \times {}^{2}\mathbf{f}_{HR,2} = \mathbf{0} $$

for Fig.3(c):

$$ {}^{3}\mathbf{f}_{HR,3} + {}^{3}\mathbf{f}_{p3} + {}^{3}\mathbf{f}_{s3} = \mathbf{0} $$
$$ {}^{3}\boldsymbol{\tau}_{s3} + {}^{3}\boldsymbol{\tau}_{p3} + {}^{3}\mathbf{r}_{c3} \times {}^{3}\mathbf{f}_{HR,3} = \mathbf{0} $$

In (33)

$$
\begin{aligned}
\hat{\boldsymbol{v}}_{H,i} &= [v_{Hy,i}\, v_{Hx,i}\, \omega_{Hz,i}]^T = J_{H,i}\dot{\mathbf{q}}_H \\
J_{H,1} &= \begin{bmatrix} -\ell_1 s_1 & 0 & 0 \\ \ell_1 c_1 & 0 & 0 \\ 1 & 0 & 0 \end{bmatrix} \\
J_{H,2} &= \begin{bmatrix} -2\ell_1 s_1 - \ell_2 s_{12} & -\ell_2 s_{12} & 0 \\ 2\ell_1 c_1 + \ell_2 c_{12} & \ell_2 c_{12} & 0 \\ 1 & 1 & 0 \end{bmatrix} \\
J_{H,3} &= \begin{bmatrix} j_{11} & j_{12} & j_{13} \\ j_{21} & j_{22} & j_{23} \\ 1 & 1 & 1 \end{bmatrix}
\end{aligned}
$$

$$ j_{11} = -2\ell_1 s_1 - 2\ell_2 s_{12} - \ell_3 s_{123} \quad j_{13} = -\ell_3 s_{123} $$
$$ j_{12} = -2\ell_2 s_{12} - \ell_3 s_{123} \quad j_{22} = 2\ell_2 c_{12} + \ell_3 c_{123} $$
$$ j_{21} = 2\ell_1 c_1 + 2\ell_2 c_{12} + \ell_3 c_{123} \quad j_{23} = \ell_3 c_{123} $$

where, $\hat{\boldsymbol{v}}_{H,i}$ is a vector combines the planar linear and angular velocity components, ℓ_i is the length of ith link, $s_i = \sin(q_i)$, $c_i = \cos(q_i)$, $s_{ij} = \sin(q_i + q_j)$, $c_{ij} = \cos(q_i + q_j)$, $s_{ijk} = \sin(q_i + q_j + q_k)$, and $c_{ijk} = \cos(q_i + q_j + q_k)$.

In (28),

$$
\begin{aligned}
a_{b,13} &= 315\,C_3\,b^{13}\,r_0 + 315\,C_3\,b^{13}\,t\\
a_{b,12} &= 3735\,C_3\,L\,b^{12}\,r_0 + 3735\,C_3\,L\,b^{12}\,t\\
a_{b,11} &= 280\,C_2\,L^2\,b^{11}\,r_0 + 18570\,C_3\,L^2\,b^{11}\,r_0 + 280\,C_2\,L^2\,b^{11}\,t + 18570\,C_3\,L^2\,b^{11}\,t\\
a_{b,10} &= 2744\,C_2\,L^3\,b^{10}\,r_0 + 49866\,C_3\,L^3\,b^{10}\,r_0 + 2744\,C_2\,L^3\,b^{10}\,t + 49866\,C_3\,L^3\,b^{10}\,t\\
a_{b,9} &= 210\,C_1\,L^4\,b^9\,r_0 + 11200\,C_2\,L^4\,b^9\,r_0 + 76935\,C_3\,L^4\,b^9\,r_0 + 210\,C_1\,L^4\,b^9\,t + 11200\,C_2\,L^4\,b^9\,t\\
&\quad + 76935\,C_3\,L^4\,b^9\,t\\
a_{b,8} &= 1610\,C_1\,L^5\,b^8\,r_0 + 24640\,C_2\,L^5\,b^8\,r_0 + 64875\,C_3\,L^5\,b^8\,r_0 + 1610\,C_1\,L^5\,b^8\,t + 24640\,C_2\,L^5\,b^8\,t\\
&\quad + 64875\,C_3\,L^5\,b^8\,t\\
a_{b,7} &= 5320\,C_1\,L^6\,b^7\,r_0 + 30800\,C_2\,L^6\,b^7\,r_0 + 22740\,C_3\,L^6\,b^7\,r_0 + 5320\,C_1\,L^6\,b^7\,t + 30800\,C_2\,L^6\,b^7\,t\\
&\quad + 22740\,C_3\,L^6\,b^7\,t\\
a_{b,6} &= 9800\,C_1\,L^7\,b^6\,r_0 + 19600\,C_2\,L^7\,b^6\,r_0 + 2100\,C_3\,L^7\,b^6\,r_0 + 9800\,C_1\,L^7\,b^6\,t + 19600\,C_2\,L^7\,b^6\,t\\
&\quad + 2100\,C_3\,L^7\,b^6\,t\\
a_{b,5} &= 11690\,C_1\,L^8\,b^5\,r_0 - 2296\,C_2\,L^8\,b^5\,r_0 + 28770\,C_3\,L^8\,b^5\,r_0 + 11690\,C_1\,L^8\,b^5\,t - 2296\,C_2\,L^8\,b^5\,t\\
&\quad + 28770\,C_3\,L^8\,b^5\,t + 840\,C_1\,L^8\,b^5\,r_0\log(L) - 3360\,C_2\,L^8\,b^5\,r_0\log(L) + 12600\,C_3\,L^8\,b^5\,r_0\log(L)\\
&\quad + 840\,C_1\,L^8\,b^5\,t\log(L) - 3360\,C_2\,L^8\,b^5\,t\log(L) + 12600\,C_3\,L^8\,b^5\,t\log(L) - 840\,C_1\,L^8\,b^5\,r_0\,\sigma_1\\
&\quad + 3360\,C_2\,L^8\,b^5\,r_0\,\sigma_1 - 12600\,C_3\,L^8\,b^5\,r_0\,\sigma_1 - 840\,C_1\,L^8\,b^5\,t\,\sigma_1 + 3360\,C_2\,L^8\,b^5\,t\,\sigma_1\\
&\quad - 12600\,C_3\,L^8\,b^5\,t\,\sigma_1\\
a_{b,4} &= 10570\,C_1\,L^9\,b^4\,r_0 - 21560\,C_2\,L^9\,b^4\,r_0 + 80850\,C_3\,L^9\,b^4\,r_0 + 10570\,C_1\,L^9\,b^4\,t - 21560\,C_2\,L^9\,b^4\,t\\
&\quad + 80850\,C_3\,L^9\,b^4\,t + 4200\,C_1\,L^9\,b^4\,r_0\log(L) - 16800\,C_2\,L^9\,b^4\,r_0\log(L) + 63000\,C_3\,L^9\,b^4\,r_0\log(L)\\
&\quad + 4200\,C_1\,L^9\,b^4\,t\log(L) - 16800\,C_2\,L^9\,b^4\,t\log(L) + 63000\,C_3\,L^9\,b^4\,t\log(L) - 4200\,C_1\,L^9\,b^4\,r_0\,\sigma_1\\
&\quad + 16800\,C_2\,L^9\,b^4\,r_0\,\sigma_1 - 63000\,C_3\,L^9\,b^4\,r_0\,\sigma_1 - 4200\,C_1\,L^9\,b^4\,t\,\sigma_1 + 16800\,C_2\,L^9\,b^4\,t\,\sigma_1\\
&\quad - 63000\,C_3\,L^9\,b^4\,t\,\sigma_1
\end{aligned}
$$

$$a_{b,3} = 7700\, C_1\, L^{10}\, b^3\, r_0 - 26320\, C_2\, L^{10}\, b^3\, r_0 + 98700\, C_3\, L^{10}\, b^3\, r_0 + 7700\, C_1\, L^{10}\, b^3\, t - 26320\, C_2\, L^{10}\, b^3\, t + 98700\, C_3\, L^{10}\, b^3\, t + 8400\, C_1\, L^{10}\, b^3\, r_0 \log(L) - 33600\, C_2\, L^{10}\, b^3\, r_0 \log(L) + 126000\, C_3\, L^{10}\, b^3\, r_0 \log(L) + 8400\, C_1\, L^{10}\, b^3\, t \log(L) - 33600\, C_2\, L^{10}\, b^3\, t \log(L) + 126000\, C_3\, L^{10}\, b^3\, t \log(L) - 8400\, C_1\, L^{10}\, b^3\, r_0\, \sigma_1 + 33600\, C_2\, L^{10}\, b^3\, r_0\, \sigma_1 - 126000\, C_3\, L^{10}\, b^3\, r_0\, \sigma_1 - 8400\, C_1\, L^{10}\, b^3\, t\, \sigma_1 + 33600\, C_2\, L^{10}\, b^3\, t\, \sigma_1 - 126000\, C_3\, L^{10}\, b^3\, t\, \sigma_1$$

$$a_{b,2} = 3780\, C_1\, L^{11}\, b^2\, r_0 - 15120\, C_2\, L^{11}\, b^2\, r_0 + 56700\, C_3\, L^{11}\, b^2\, r_0 + 3780\, C_1\, L^{11}\, b^2\, t - 15120\, C_2\, L^{11}\, b^2\, t + 56700\, C_3\, L^{11}\, b^2\, t + 8400\, C_1\, L^{11}\, b^2\, r_0 \log(L) - 33600\, C_2\, L^{11}\, b^2\, r_0 \log(L) + 126000\, C_3\, L^{11}\, b^2\, r_0 \log(L) + 8400\, C_1\, L^{11}\, b^2\, t \log(L) - 33600\, C_2\, L^{11}\, b^2\, t \log(L) + 126000\, C_3\, L^{11}\, b^2\, t \log(L) - 8400\, C_1\, L^{11}\, b^2\, r_0\, \sigma_1 + 33600\, C_2\, L^{11}\, b^2\, r_0\, \sigma_1 - 126000\, C_3\, L^{11}\, b^2\, r_0\, \sigma_1 - 8400\, C_1\, L^{11}\, b^2\, t\, \sigma_1 + 33600\, C_2\, L^{11}\, b^2\, t\, \sigma_1 - 126000\, C_3\, L^{11}\, b^2\, t\, \sigma_1$$

$$a_{b,1} = 840\, C_1\, L^{12}\, b\, r_0 - 3360\, C_2\, L^{12}\, b\, r_0 + 12600\, C_3\, L^{12}\, b\, r_0 + 840\, C_1\, L^{12}\, b\, t - 3360\, C_2\, L^{12}\, b\, t + 12600\, C_3\, L^{12}\, b\, t - 4200\, C_1\, L^{12}\, b\, r_0\, \sigma_1 + 16800\, C_2\, L^{12}\, b\, r_0\, \sigma_1 - 63000\, C_3\, L^{12}\, b\, r_0\, \sigma_1 - 4200\, C_1\, L^{12}\, b\, t\, \sigma_1 + 16800\, C_2\, L^{12}\, b\, t\, \sigma_1 - 63000\, C_3\, L^{12}\, b\, t\, \sigma_1 + 4200\, C_1\, L^{12}\, b\, r_0 \log(L) - 16800\, C_2\, L^{12}\, b\, r_0 \log(L) + 63000\, C_3\, L^{12}\, b\, r_0 \log(L) + 4200\, C_1\, L^{12}\, b\, t \log(L) - 16800\, C_2\, L^{12}\, b\, t \log(L) + 63000\, C_3\, L^{12}\, b\, t \log(L)$$

$$a_{b,0} = 3360\, C_2\, L^{13}\, r_0\, \sigma_1 - 840\, C_1\, L^{13}\, r_0\, \sigma_1 - 12600\, C_3\, L^{13}\, r_0\, \sigma_1 - 840\, C_1\, L^{13}\, t\, \sigma_1 + 3360\, C_2\, L^{13}\, t\, \sigma_1 - 12600\, C_3\, L^{13}\, t\, \sigma_1 + 840\, C_1\, L^{13}\, r_0 \log(L) - 3360\, C_2\, L^{13}\, r_0 \log(L) + 12600\, C_3\, L^{13}\, r_0 \log(L) + 840\, C_1\, L^{13}\, t \log(L) - 3360\, C_2\, L^{13}\, t \log(L) + 12600\, C_3\, L^{13}\, t \log(L)$$

$$b_{b,7} = 210\, L^6\, b^5 \quad b_{b,6} = 1050\, L^7\, b^4 \quad b_{b,5} = 2100\, L^8\, b^3 \quad b_{b,4} = 2100\, L^9\, b^2 \quad b_{b,3} = 1050\, L^{10}\, b$$

$$b_{b,2} = 210\, L^{11}$$

where
$\sigma_1 = \log(L + b\, q)$

$$a_{h,18} = 2520\, C_3\, t\, b^{17}\, h + 21420\, C_3\, t\, b^{16}\, h^2 + 86940\, C_3\, t\, b^{15}\, h^3 + 223650\, C_3\, t\, b^{14}\, h^4 + 405090\, C_3\, t\, b^{13}\, h^5 + 540540\, C_3\, t\, b^{12}\, h^6 + 540540\, C_3\, t\, b^{11}\, h^7 + 405405\, C_3\, t\, b^{10}\, h^8 + 225225\, C_3\, t\, b^9\, h^9 + 90090\, C_3\, t\, b^8\, h^{10} + 24570\, C_3\, t\, b^7\, h^{11} + 4095\, C_3\, t\, b^6\, h^{12} + 315\, C_3\, t\, b^5\, h^{13}$$

$$a_{h,17} = 40320\, C_3\, L\, t\, b^{16}\, h + 322560\, C_3\, L\, t\, b^{15}\, h^2 + 1228500\, C_3\, L\, t\, b^{14}\, h^3 + 2954700\, C_3\, L\, t\, b^{13}\, h^4 + 4981410\, C_3\, L\, t\, b^{12}\, h^5 + 6153840\, C_3\, L\, t\, b^{11}\, h^6 + 5660820\, C_3\, L\, t\, b^{10}\, h^7 + 3875850\, C_3\, L\, t\, b^9\, h^8 + 1947825\, C_3\, L\, t\, b^8\, h^9 + 696960\, C_3\, L\, t\, b^7\, h^{10} + 167670\, C_3\, L\, t\, b^6\, h^{11} + 24210\, C_3\, L\, t\, b^5\, h^{12} + 1575\, C_3\, L\, t\, b^4\, h^{13}$$

$$a_{h,16} = 280\, C_2\, L^2\, b^5\, h^{11}\, t + 3080\, C_2\, L^2\, b^6\, h^{10}\, t + 15400\, C_2\, L^2\, b^7\, h^9\, t + 46200\, C_2\, L^2\, b^8\, h^8\, t + 92400\, C_2\, L^2\, b^9\, h^7\, t + 129360\, C_2\, L^2\, b^{10}\, h^6\, t + 129080\, C_2\, L^2\, b^{11}\, h^5\, t + 91000\, C_2\, L^2\, b^{12}\, h^4\, t + 43400\, C_2\, L^2\, b^{13}\, h^3\, t + 12600\, C_2\, L^2\, b^{14}\, h^2\, t + 3150\, C_3\, L^2\, b^3\, h^{13}\, t + 59625\, C_3\, L^2\, b^4\, h^{12}\, t + 488370\, C_3\, L^2\, b^5\, h^{11}\, t + 2337720\, C_3\, L^2\, b^6\, h^{10}\, t + 7382100\, C_3\, L^2\, b^7\, h^9\, t + 16362225\, C_3\, L^2\, b^8\, h^8\, t$$

$$+ 26324100\, C_3\, L^2\, b^9\, h^7\, t + 31240440\, C_3\, L^2\, b^{10}\, h^6\, t + 27405420\, C_3\, L^2\, b^{11}\, h^5\, t$$
$$+ 17512950\, C_3\, L^2\, b^{12}\, h^4\, t + 7809900\, C_3\, L^2\, b^{13}\, h^3\, t + 2192400\, C_3\, L^2\, b^{14}\, h^2\, t + 1680\, C_2\, L^2\, b^{15}\, h\, t$$
$$+ 292320\, C_3\, L^2\, b^{15}\, h\, t$$
$$a_{h,15} = 1400\, C_2\, L^3\, b^4\, h^{11}\, t + 18144\, C_2\, L^3\, b^5\, h^{10}\, t + 104440\, C_2\, L^3\, b^6\, h^9\, t + 354480\, C_2\, L^3\, b^7\, h^8\, t$$
$$+ 791280\, C_2\, L^3\, b^8\, h^7\, t + 1223040\, C_2\, L^3\, b^9\, h^6\, t + 1335544\, C_2\, L^3\, b^{10}\, h^5\, t + 1023120\, C_2\, L^3\, b^{11}\, h^4\, t$$
$$+ 527240\, C_2\, L^3\, b^{12}\, h^3\, t + 164640\, C_2\, L^3\, b^{13}\, h^2\, t + 3150\, C_3\, L^3\, b^2\, h^{13}\, t + 78300\, C_3\, L^3\, b^3\, h^{12}\, t$$
$$+ 786750\, C_3\, L^3\, b^4\, h^{11}\, t + 4437216\, C_3\, L^3\, b^5\, h^{10}\, t + 16074660\, C_3\, L^3\, b^6\, h^9\, t$$
$$+ 40106520\, C_3\, L^3\, b^7\, h^8\, t + 71611020\, C_3\, L^3\, b^8\, h^7\, t + 93285360\, C_3\, L^3\, b^9\, h^6\, t + 89048316\, C_3\, L^3\, b^{10}\, h^5\, t$$
$$+ 61510680\, C_3\, L^3\, b^{11}\, h^4\, t + 29514660\, C_3\, L^3\, b^{12}\, h^3\, t + 8890560\, C_3\, L^3\, b^{13}\, h^2\, t$$
$$+ 23520\, C_2\, L^3\, b^{14}\, h\, t + 1270080\, C_3\, L^3\, b^{14}\, h\, t$$
$$a_{h,14} = 210\, C_1\, L^4\, b^5\, h^9\, t + 1890\, C_1\, L^4\, b^6\, h^8\, t + 7560\, C_1\, L^4\, b^7\, h^7\, t + 17640\, C_1\, L^4\, b^8\, h^6\, t$$
$$+ 26250\, C_1\, L^4\, b^9\, h^5\, t + 25410\, C_1\, L^4\, b^{10}\, h^4\, t + 15540\, C_1\, L^4\, b^{11}\, h^3\, t + 5460\, C_1\, L^4\, b^{12}\, h^2\, t$$
$$+ 2800\, C_2\, L^4\, b^3\, h^{11}\, t + 44520\, C_2\, L^4\, b^4\, h^{10}\, t + 302400\, C_2\, L^4\, b^5\, h^9\, t + 1180200\, C_2\, L^4\, b^6\, h^8\, t$$
$$+ 2973600\, C_2\, L^4\, b^7\, h^7\, t + 5115600\, C_2\, L^4\, b^8\, h^6\, t + 6151040\, C_2\, L^4\, b^9\, h^5\, t + 5146680\, C_2\, L^4\, b^{10}\, h^4\, t$$
$$+ 2879520\, C_2\, L^4\, b^{11}\, h^3\, t + 971880\, C_2\, L^4\, b^{12}\, h^2\, t + 57825\, C_3\, L^4\, b^2\, h^{12}\, t + 756750\, C_3\, L^4\, b^3\, h^{11}\, t$$
$$+ 5207580\, C_3\, L^4\, b^4\, h^{10}\, t + 22126860\, C_3\, L^4\, b^5\, h^9\, t + 63068040\, C_3\, L^4\, b^6\, h^8\, t + 126254160\, C_3\, L^4\, b^7\, h^7\, t$$
$$+ 181829340\, C_3\, L^4\, b^8\, h^6\, t + 189849660\, C_3\, L^4\, b^9\, h^5\, t + 142314480\, C_3\, L^4\, b^{10}\, h^4\, t$$
$$+ 73737720\, C_3\, L^4\, b^{11}\, h^3\, t + 23931180\, C_3\, L^4\, b^{12}\, h^2\, t + 840\, C_1\, L^4\, b^{13}\, h\, t + 149520\, C_2\, L^4\, b^{13}\, h\, t$$
$$+ 1575\, C_3\, L^4\, b\, h^{13}\, t + 3681720\, C_3\, L^4\, b^{13}\, h\, t$$
$$a_{h,13} = 315\, C_3\, L^5\, h^{13}\, t + 1050\, C_1\, L^5\, b^4\, h^9\, t + 11060\, C_1\, L^5\, b^5\, h^8\, t + 50680\, C_1\, L^5\, b^6\, h^7\, t$$
$$+ 133280\, C_1\, L^5\, b^7\, h^6\, t + 220850\, C_1\, L^5\, b^8\, h^5\, t + 235900\, C_1\, L^5\, b^9\, h^4\, t + 158060\, C_1\, L^5\, b^{10}\, h^3\, t$$
$$+ 60480\, C_1\, L^5\, b^{11}\, h^2\, t + 2800\, C_2\, L^5\, b^2\, h^{11}\, t + 58240\, C_2\, L^5\, b^3\, h^{10}\, t + 484400\, C_2\, L^5\, b^4\, h^9\, t$$
$$+ 2225440\, C_2\, L^5\, b^5\, h^8\, t + 6429920\, C_2\, L^5\, b^6\, h^7\, t + 12449920\, C_2\, L^5\, b^7\, h^6\, t + 16619680\, C_2\, L^5\, b^8\, h^5\, t$$
$$+ 15288000\, C_2\, L^5\, b^9\, h^4\, t + 9340800\, C_2\, L^5\, b^{10}\, h^3\, t + 3427200\, C_2\, L^5\, b^{11}\, h^2\, t + 434370\, C_3\, L^5\, b^2\, h^{11}\, t$$
$$+ 3864000\, C_3\, L^5\, b^3\, h^{10}\, t + 19918500\, C_3\, L^5\, b^4\, h^9\, t + 66256680\, C_3\, L^5\, b^5\, h^8\, t$$
$$+ 150818640\, C_3\, L^5\, b^6\, h^7\, t + 242437440\, C_3\, L^5\, b^7\, h^6\, t + 278688900\, C_3\, L^5\, b^8\, h^5\, t$$
$$+ 227770200\, C_3\, L^5\, b^9\, h^4\, t + 127937880\, C_3\, L^5\, b^{10}\, h^3\, t + 44936640\, C_3\, L^5\, b^{11}\, h^2\, t$$
$$+ 10080\, C_1\, L^5\, b^{12}\, h\, t + 571200\, C_2\, L^5\, b^{12}\, h\, t + 22770\, C_3\, L^5\, b\, h^{12}\, t + 7489440\, C_3\, L^5\, b^{12}\, h\, t$$
$$a_{h,12} = 3735\, C_3\, L^6\, h^{12}\, t + 2100\, C_1\, L^6\, b^3\, h^9\, t + 26950\, C_1\, L^6\, b^4\, h^8\, t + 145320\, C_1\, L^6\, b^5\, h^7\, t$$
$$+ 439040\, C_1\, L^6\, b^6\, h^6\, t + 821800\, C_1\, L^6\, b^7\, h^5\, t + 979650\, C_1\, L^6\, b^8\, h^4\, t + 725900\, C_1\, L^6\, b^9\, h^3\, t$$
$$+ 304920\, C_1\, L^6\, b^{10}\, h^2\, t + 42840\, C_2\, L^6\, b^2\, h^{10}\, t + 463400\, C_2\, L^6\, b^3\, h^9\, t + 2597000\, C_2\, L^6\, b^4\, h^8\, t$$
$$+ 8803200\, C_2\, L^6\, b^5\, h^7\, t + 19482400\, C_2\, L^6\, b^6\, h^6\, t + 29188880\, C_2\, L^6\, b^7\, h^5\, t$$
$$+ 29761200\, C_2\, L^6\, b^8\, h^4\, t + 19996200\, C_2\, L^6\, b^9\, h^3\, t + 8029560\, C_2\, L^6\, b^{10}\, h^2\, t$$
$$+ 1766520\, C_3\, L^6\, b^2\, h^{10}\, t + 11684400\, C_3\, L^6\, b^3\, h^9\, t + 46857300\, C_3\, L^6\, b^4\, h^8\, t$$

$$
\begin{aligned}
& + 123752160\, C_3\, L^6\, b^5\, h^7\, t + 224933520\, C_3\, L^6\, b^6\, h^6\, t + 287075040\, C_3\, L^6\, b^7\, h^5\, t \\
& + 257210100\, C_3\, L^6\, b^8\, h^4\, t + 157273200\, C_3\, L^6\, b^9\, h^3\, t + 60069240\, C_3\, L^6\, b^{10}\, h^2\, t \\
& + 55440\, C_1\, L^6\, b^{11}\, h\, t + 1400\, C_2\, L^6\, b\, h^{11}\, t + 1459920\, C_2\, L^6\, b^{11}\, h\, t + 137670\, C_3\, L^6\, b\, h^{11}\, t \\
& + 10921680\, C_3\, L^6\, b^{11}\, h\, t \\
a_{h,11} &= 280\, C_2\, L^7\, h^{11}\, t + 18570\, C_3\, L^7\, h^{11}\, t + 2100\, C_1\, L^7\, b^2\, h^9\, t + 35000\, C_1\, L^7\, b^3\, h^8\, t \\
& + 231000\, C_1\, L^7\, b^4\, h^7\, t + 823200\, C_1\, L^7\, b^5\, h^6\, t + 1773800\, C_1\, L^7\, b^6\, h^5\, t + 2394000\, C_1\, L^7\, b^7\, h^4\, t \\
& + 1984500\, C_1\, L^7\, b^8\, h^3\, t + 924000\, C_1\, L^7\, b^9\, h^2\, t + 264600\, C_2\, L^7\, b^2\, h^9\, t + 1918000\, C_2\, L^7\, b^3\, h^8\, t \\
& + 7896000\, C_2\, L^7\, b^4\, h^7\, t + 20415360\, C_2\, L^7\, b^5\, h^6\, t + 34829200\, C_2\, L^7\, b^6\, h^5\, t + 39765600\, C_2\, L^7\, b^7\, h^4\, t \\
& + 29622600\, C_2\, L^7\, b^8\, h^3\, t + 13120800\, C_2\, L^7\, b^9\, h^2\, t + 4284000\, C_3\, L^7\, b^2\, h^9\, t + 21856800\, C_3\, L^7\, b^3\, h^8\, t \\
& + 69048000\, C_3\, L^7\, b^4\, h^7\, t + 144527040\, C_3\, L^7\, b^5\, h^6\, t + 207076800\, C_3\, L^7\, b^6\, h^5\, t + 204724800\, C_3\, L^7\, b^7\, h^4\, t \\
& + 136836000\, C_3\, L^7\, b^8\, h^3\, t + 57103200\, C_3\, L^7\, b^9\, h^2\, t + 184800\, C_1\, L^7\, b^{10}\, h\, t + 16800\, C_2\, L^7\, b\, h^{10}\, t \\
& + 2624160\, C_2\, L^7\, b^{10}\, h\, t + 453600\, C_3\, L^7\, b\, h^{10}\, t + 11420640\, C_3\, L^7\, b^{10}\, h\, t \\
a_{h,10} &= 2744\, C_2\, L^8\, h^{10}\, t + 49866\, C_3\, L^8\, h^{10}\, t - 840\, C_1\, L^8\, b^{10}\, t\, \sigma_1 + 3360\, C_2\, L^8\, b^{10}\, t\, \sigma_1 - 12600\, C_3\, L^8\, b^{10}\, t\, \sigma_1 \\
& + 25550\, C_1\, L^8\, b^2\, h^8\, t + 219800\, C_1\, L^8\, b^3\, h^7\, t + 960400\, C_1\, L^8\, b^4\, h^6\, t + 2445100\, C_1\, L^8\, b^5\, h^5\, t \\
& + 3807300\, C_1\, L^8\, b^6\, h^4\, t + 3582600\, C_1\, L^8\, b^7\, h^3\, t + 1871100\, C_1\, L^8\, b^8\, h^2\, t + 873880\, C_2\, L^8\, b^2\, h^8\, t \\
& + 4624480\, C_2\, L^8\, b^3\, h^7\, t + 14433440\, C_2\, L^8\, b^4\, h^6\, t + 28601888\, C_2\, L^8\, b^5\, h^5\, t + 37032240\, C_2\, L^8\, b^6\, h^4\, t \\
& + 30871680\, C_2\, L^8\, b^7\, h^3\, t + 15218280\, C_2\, L^8\, b^8\, h^2\, t + 6354795\, C_3\, L^8\, b^2\, h^8\, t + 25249620\, C_3\, L^8\, b^3\, h^7\, t \\
& + 62551860\, C_3\, L^8\, b^4\, h^6\, t + 102203682\, C_3\, L^8\, b^5\, h^5\, t + 112459410\, C_3\, L^8\, b^6\, h^4\, t + 82526220\, C_3\, L^8\, b^7\, h^3\, t \\
& + 37796220\, C_3\, L^8\, b^8\, h^2\, t + 840\, C_1\, L^8\, b^{10}\, t\, \sigma_2 - 3360\, C_2\, L^8\, b^{10}\, t\, \sigma_2 + 12600\, C_3\, L^8\, b^{10}\, t\, \sigma_2 \\
& + 1050\, C_1\, L^8\, b\, h^9\, t + 415800\, C_1\, L^8\, b^9\, h\, t + 83440\, C_2\, L^8\, b\, h^9\, t + 3381840\, C_2\, L^8\, b^9\, h\, t \\
& + 883335\, C_3\, L^8\, b\, h^9\, t + 8399160\, C_3\, L^8\, b^9\, h\, t + 4200\, C_1\, L^8\, b^9\, h\, t\, \sigma_2 - 16800\, C_2\, L^8\, b^9\, h\, t\, \sigma_2 \\
& + 63000\, C_3\, L^8\, b^9\, h\, t\, \sigma_2 - 4200\, C_1\, L^8\, b^9\, h\, t\, \sigma_1 + 16800\, C_2\, L^8\, b^9\, h\, t\, \sigma_1 - 63000\, C_3\, L^8\, b^9\, h\, t\, \sigma_1 \\
& + 840\, C_1\, L^8\, b^5\, h^5\, t\, \sigma_2 + 4200\, C_1\, L^8\, b^6\, h^4\, t\, \sigma_2 + 8400\, C_1\, L^8\, b^7\, h^3\, t\, \sigma_2 + 8400\, C_1\, L^8\, b^8\, h^2\, t\, \sigma_2 \\
& - 3360\, C_2\, L^8\, b^5\, h^5\, t\, \sigma_2 - 16800\, C_2\, L^8\, b^6\, h^4\, t\, \sigma_2 - 33600\, C_2\, L^8\, b^7\, h^3\, t\, \sigma_2 - 33600\, C_2\, L^8\, b^8\, h^2\, t\, \sigma_2 \\
& + 12600\, C_3\, L^8\, b^5\, h^5\, t\, \sigma_2 + 63000\, C_3\, L^8\, b^6\, h^4\, t\, \sigma_2 + 126000\, C_3\, L^8\, b^7\, h^3\, t\, \sigma_2 + 126000\, C_3\, L^8\, b^8\, h^2\, t\, \sigma_2 \\
& - 840\, C_1\, L^8\, b^5\, h^5\, t\, \sigma_1 - 4200\, C_1\, L^8\, b^6\, h^4\, t\, \sigma_1 - 8400\, C_1\, L^8\, b^7\, h^3\, t\, \sigma_1 - 8400\, C_1\, L^8\, b^8\, h^2\, t\, \sigma_1 \\
& + 3360\, C_2\, L^8\, b^5\, h^5\, t\, \sigma_1 + 16800\, C_2\, L^8\, b^6\, h^4\, t\, \sigma_1 + 33600\, C_2\, L^8\, b^7\, h^3\, t\, \sigma_1 + 33600\, C_2\, L^8\, b^8\, h^2\, t\, \sigma_1 \\
& - 12600\, C_3\, L^8\, b^5\, h^5\, t\, \sigma_1 - 63000\, C_3\, L^8\, b^6\, h^4\, t\, \sigma_1 - 126000\, C_3\, L^8\, b^7\, h^3\, t\, \sigma_1 - 126000\, C_3\, L^8\, b^8\, h^2\, t\, \sigma_1 \\
a_{h,9} &= 210\, C_1\, L^9\, h^9\, t + 11200\, C_2\, L^9\, h^9\, t + 76935\, C_3\, L^9\, h^9\, t - 8400\, C_1\, L^9\, b^9\, t\, \sigma_1 + 33600\, C_2\, L^9\, b^9\, t\, \sigma_1 \\
& - 126000\, C_3\, L^9\, b^9\, t\, \sigma_1 + 125160\, C_1\, L^9\, b^2\, h^7\, t + 713440\, C_1\, L^9\, b^3\, h^6\, t + 2230340\, C_1\, L^9\, b^4\, h^5\, t \\
& + 4113480\, C_1\, L^9\, b^5\, h^4\, t + 4479720\, C_1\, L^9\, b^6\, h^3\, t + 2664480\, C_1\, L^9\, b^7\, h^2\, t + 1696800\, C_2\, L^9\, b^2\, h^7\, t \\
& + 6742400\, C_2\, L^9\, b^3\, h^6\, t + 15964480\, C_2\, L^9\, b^4\, h^5\, t + 23795520\, C_2\, L^9\, b^5\, h^4\, t + 22404480\, C_2\, L^9\, b^6\, h^3\, t \\
& + 12405120\, C_2\, L^9\, b^7\, h^2\, t + 5592060\, C_3\, L^9\, b^2\, h^7\, t + 17157840\, C_3\, L^9\, b^3\, h^6\, t + 32823210\, C_3\, L^9\, b^4\, h^5\, t \\
& + 40926060\, C_3\, L^9\, b^5\, h^4\, t + 33363540\, C_3\, L^9\, b^6\, h^3\, t + 16904160\, C_3\, L^9\, b^7\, h^2\, t + 8400\, C_1\, L^9\, b^9\, t\, \sigma_2
\end{aligned}
$$

$$
\begin{aligned}
&- 33600\, C_2\, L^9\, b^9\, t\, \sigma_2 + 126000\, C_3\, L^9\, b^9\, t\, \sigma_2 + 9940\, C_1\, L^9\, b\, h^8\, t + 666120\, C_1\, L^9\, b^8\, h\, t \\
&+ 224000\, C_2\, L^9\, b\, h^8\, t + 3101280\, C_2\, L^9\, b^8\, h\, t + 1016790\, C_3\, L^9\, b\, h^8\, t + 4226040\, C_3\, L^9\, b^8\, h\, t \\
&+ 37800\, C_1\, L^9\, b^8\, h\, t\, \sigma_2 - 151200\, C_2\, L^9\, b^8\, h\, t\, \sigma_2 + 567000\, C_3\, L^9\, b^8\, h\, t\, \sigma_2 \\
&- 37800\, C_1\, L^9\, b^8\, h\, t\, \sigma_1 + 151200\, C_2\, L^9\, b^8\, h\, t\, \sigma_1 - 567000\, C_3\, L^9\, b^8\, h\, t\, \sigma_1 \\
&+ 4200\, C_1\, L^9\, b^4\, h^5\, t\, \sigma_2 + 25200\, C_1\, L^9\, b^5\, h^4\, t\, \sigma_2 + 58800\, C_1\, L^9\, b^6\, h^3\, t\, \sigma_2 \\
&+ 67200\, C_1\, L^9\, b^7\, h^2\, t\, \sigma_2 - 16800\, C_2\, L^9\, b^4\, h^5\, t\, \sigma_2 - 100800\, C_2\, L^9\, b^5\, h^4\, t\, \sigma_2 \\
&- 235200\, C_2\, L^9\, b^6\, h^3\, t\, \sigma_2 - 268800\, C_2\, L^9\, b^7\, h^2\, t\, \sigma_2 + 63000\, C_3\, L^9\, b^4\, h^5\, t\, \sigma_2 \\
&+ 378000\, C_3\, L^9\, b^5\, h^4\, t\, \sigma_2 + 882000\, C_3\, L^9\, b^6\, h^3\, t\, \sigma_2 + 1008000\, C_3\, L^9\, b^7\, h^2\, t\, \sigma_2 \\
&- 4200\, C_1\, L^9\, b^4\, h^5\, t\, \sigma_1 - 25200\, C_1\, L^9\, b^5\, h^4\, t\, \sigma_1 - 58800\, C_1\, L^9\, b^6\, h^3\, t\, \sigma_1 \\
&- 67200\, C_1\, L^9\, b^7\, h^2\, t\, \sigma_1 + 16800\, C_2\, L^9\, b^4\, h^5\, t\, \sigma_1 + 100800\, C_2\, L^9\, b^5\, h^4\, t\, \sigma_1 \\
&+ 235200\, C_2\, L^9\, b^6\, h^3\, t\, \sigma_1 + 268800\, C_2\, L^9\, b^7\, h^2\, t\, \sigma_1 - 63000\, C_3\, L^9\, b^4\, h^5\, t\, \sigma_1 \\
&- 378000\, C_3\, L^9\, b^5\, h^4\, t\, \sigma_1 - 882000\, C_3\, L^9\, b^6\, h^3\, t\, \sigma_1 - 1008000\, C_3\, L^9\, b^7\, h^2\, t\, \sigma_1 \\
a_{h,8} &= 1610\, C_1\, L^{10}\, h^8\, t + 24640\, C_2\, L^{10}\, h^8\, t + 64875\, C_3\, L^{10}\, h^8\, t - 37800\, C_1\, L^{10}\, b^8\, t\, \sigma_1 + 151200\, C_2\, L^{10}\, b^8\, t\, \sigma_1 \\
&- 567000\, C_3\, L^{10}\, b^8\, t\, \sigma_1 + 329280\, C_1\, L^{10}\, b^2\, h^6\, t + 1345960\, C_1\, L^{10}\, b^3\, h^5\, t + 3059700\, C_1\, L^{10}\, b^4\, h^4\, t \\
&+ 3963960\, C_1\, L^{10}\, b^5\, h^3\, t + 2740080\, C_1\, L^{10}\, b^6\, h^2\, t + 1963920\, C_2\, L^{10}\, b^2\, h^6\, t + 5793200\, C_2\, L^{10}\, b^3\, h^5\, t \\
&+ 10071600\, C_2\, L^{10}\, b^4\, h^4\, t + 10720080\, C_2\, L^{10}\, b^5\, h^3\, t + 6691440\, C_2\, L^{10}\, b^6\, h^2\, t + 2633400\, C_3\, L^{10}\, b^2\, h^6\, t \\
&+ 6335700\, C_3\, L^{10}\, b^3\, h^5\, t + 9780750\, C_3\, L^{10}\, b^4\, h^4\, t + 9733500\, C_3\, L^{10}\, b^5\, h^3\, t \\
&+ 5821200\, C_3\, L^{10}\, b^6\, h^2\, t + 37800\, C_1\, L^{10}\, b^8\, t\, \sigma_2 - 151200\, C_2\, L^{10}\, b^8\, t\, \sigma_2 + 567000\, C_3\, L^{10}\, b^8\, t\, \sigma_2 \\
&+ 39480\, C_1\, L^{10}\, b\, h^7\, t + 782880\, C_1\, L^{10}\, b^7\, h\, t + 351120\, C_2\, L^{10}\, b\, h^7\, t + 1911840\, C_2\, L^{10}\, b^7\, h\, t \\
&+ 632700\, C_3\, L^{10}\, b\, h^7\, t + 1663200\, C_3\, L^{10}\, b^7\, h\, t + 151200\, C_1\, L^{10}\, b^7\, h\, t\, \sigma_2 - 604800\, C_2\, L^{10}\, b^7\, h\, t\, \sigma_2 \\
&+ 2268000\, C_3\, L^{10}\, b^7\, h\, t\, \sigma_2 - 151200\, C_1\, L^{10}\, b^7\, h\, t\, \sigma_1 + 604800\, C_2\, L^{10}\, b^7\, h\, t\, \sigma_1 - 2268000\, C_3\, L^{10}\, b^7\, h\, t\, \sigma_1 \\
&+ 8400\, C_1\, L^{10}\, b^3\, h^5\, t\, \sigma_2 + 63000\, C_1\, L^{10}\, b^4\, h^4\, t\, \sigma_2 + 176400\, C_1\, L^{10}\, b^5\, h^3\, t\, \sigma_2 + 235200\, C_1\, L^{10}\, b^6\, h^2\, t\, \sigma_2 \\
&- 33600\, C_2\, L^{10}\, b^3\, h^5\, t\, \sigma_2 - 252000\, C_2\, L^{10}\, b^4\, h^4\, t\, \sigma_2 - 705600\, C_2\, L^{10}\, b^5\, h^3\, t\, \sigma_2 - 940800\, C_2\, L^{10}\, b^6\, h^2\, t\, \sigma_2 \\
&+ 126000\, C_3\, L^{10}\, b^3\, h^5\, t\, \sigma_2 + 945000\, C_3\, L^{10}\, b^4\, h^4\, t\, \sigma_2 + 2646000\, C_3\, L^{10}\, b^5\, h^3\, t\, \sigma_2 \\
&+ 3528000\, C_3\, L^{10}\, b^6\, h^2\, t\, \sigma_2 - 8400\, C_1\, L^{10}\, b^3\, h^5\, t\, \sigma_1 - 63000\, C_1\, L^{10}\, b^4\, h^4\, t\, \sigma_1 \\
&- 176400\, C_1\, L^{10}\, b^5\, h^3\, t\, \sigma_1 - 235200\, C_1\, L^{10}\, b^6\, h^2\, t\, \sigma_1 + 33600\, C_2\, L^{10}\, b^3\, h^5\, t\, \sigma_1 + 252000\, C_2\, L^{10}\, b^4\, h^4\, t\, \sigma_1 \\
&+ 705600\, C_2\, L^{10}\, b^5\, h^3\, t\, \sigma_1 + 940800\, C_2\, L^{10}\, b^6\, h^2\, t\, \sigma_1 - 126000\, C_3\, L^{10}\, b^3\, h^5\, t\, \sigma_1 \\
&- 945000\, C_3\, L^{10}\, b^4\, h^4\, t\, \sigma_1 - 2646000\, C_3\, L^{10}\, b^5\, h^3\, t\, \sigma_1 - 3528000\, C_3\, L^{10}\, b^6\, h^2\, t\, \sigma_1 \\
a_{h,7} &= 5320\, C_1\, L^{11}\, h^7\, t + 30800\, C_2\, L^{11}\, h^7\, t + 22740\, C_3\, L^{11}\, h^7\, t - 100800\, C_1\, L^{11}\, b^7\, t\, \sigma_1 \\
&+ 403200\, C_2\, L^{11}\, b^7\, t\, \sigma_1 - 1512000\, C_3\, L^{11}\, b^7\, t\, \sigma_1 + 518840\, C_1\, L^{11}\, b^2\, h^5\, t \\
&+ 1554000\, C_1\, L^{11}\, b^3\, h^4\, t + 2499000\, C_1\, L^{11}\, b^4\, h^3\, t + 2066400\, C_1\, L^{11}\, b^5\, h^2\, t \\
&+ 1226960\, C_2\, L^{11}\, b^2\, h^5\, t + 2424800\, C_2\, L^{11}\, b^3\, h^4\, t + 2707600\, C_2\, L^{11}\, b^4\, h^3\, t \\
&+ 1794240\, C_2\, L^{11}\, b^5\, h^2\, t + 771540\, C_3\, L^{11}\, b^2\, h^5\, t + 2423400\, C_3\, L^{11}\, b^3\, h^4\, t \\
&+ 4260900\, C_3\, L^{11}\, b^4\, h^3\, t + 3659040\, C_3\, L^{11}\, b^5\, h^2\, t + 100800\, C_1\, L^{11}\, b^7\, t\, \sigma_2
\end{aligned}
$$

$$
\begin{aligned}
&- 403200\, C_2\, L^{11}\, b^7\, t\, \sigma_2 + 1512000\, C_3\, L^{11}\, b^7\, t\, \sigma_2 + 86240\, C_1\, L^{11}\, b\, h^6\, t \\
&+ 688800\, C_1\, L^{11}\, b^6\, h\, t + 313600\, C_2\, L^{11}\, b\, h^6\, t + 598080\, C_2\, L^{11}\, b^6\, h\, t \\
&+ 169680\, C_3\, L^{11}\, b\, h^6\, t + 1219680\, C_3\, L^{11}\, b^6\, h\, t + 352800\, C_1\, L^{11}\, b^6\, h\, t\, \sigma_2 - 1411200\, C_2\, L^{11}\, b^6\, h\, t\, \sigma_2 \\
&+ 5292000\, C_3\, L^{11}\, b^6\, h\, t\, \sigma_2 - 352800\, C_1\, L^{11}\, b^6\, h\, t\, \sigma_1 + 1411200\, C_2\, L^{11}\, b^6\, h\, t\, \sigma_1 \\
&- 5292000\, C_3\, L^{11}\, b^6\, h\, t\, \sigma_1 + 8400\, C_1\, L^{11}\, b^2\, h^5\, t\, \sigma_2 + 84000\, C_1\, L^{11}\, b^3\, h^4\, t\, \sigma_2 \\
&+ 294000\, C_1\, L^{11}\, b^4\, h^3\, t\, \sigma_2 + 470400\, C_1\, L^{11}\, b^5\, h^2\, t\, \sigma_2 \\
&- 33600\, C_2\, L^{11}\, b^2\, h^5\, t\, \sigma_2 - 336000\, C_2\, L^{11}\, b^3\, h^4\, t\, \sigma_2 - 1176000\, C_2\, L^{11}\, b^4\, h^3\, t\, \sigma_2 \\
&- 1881600\, C_2\, L^{11}\, b^5\, h^2\, t\, \sigma_2 + 126000\, C_3\, L^{11}\, b^2\, h^5\, t\, \sigma_2 + 1260000\, C_3\, L^{11}\, b^3\, h^4\, t\, \sigma_2 \\
&+ 4410000\, C_3\, L^{11}\, b^4\, h^3\, t\, \sigma_2 + 7056000\, C_3\, L^{11}\, b^5\, h^2\, t\, \sigma_2 - 8400\, C_1\, L^{11}\, b^2\, h^5\, t\, \sigma_1 \\
&- 84000\, C_1\, L^{11}\, b^3\, h^4\, t\, \sigma_1 - 294000\, C_1\, L^{11}\, b^4\, h^3\, t\, \sigma_1 - 470400\, C_1\, L^{11}\, b^5\, h^2\, t\, \sigma_1 \\
&+ 33600\, C_2\, L^{11}\, b^2\, h^5\, t\, \sigma_1 + 336000\, C_2\, L^{11}\, b^3\, h^4\, t\, \sigma_1 + 1176000\, C_2\, L^{11}\, b^4\, h^3\, t\, \sigma_1 \\
&+ 1881600\, C_2\, L^{11}\, b^5\, h^2\, t\, \sigma_1 - 126000\, C_3\, L^{11}\, b^2\, h^5\, t\, \sigma_1 - 1260000\, C_3\, L^{11}\, b^3\, h^4\, t\, \sigma_1 \\
&- 4410000\, C_3\, L^{11}\, b^4\, h^3\, t\, \sigma_1 - 7056000\, C_3\, L^{11}\, b^5\, h^2\, t\, \sigma_1 \\
a_{h,6} &= 9800\, C_1\, L^{12}\, h^6\, t + 19600\, C_2\, L^{12}\, h^6\, t + 2100\, C_3\, L^{12}\, h^6\, t - 176400\, C_1\, L^{12}\, b^6\, t\, \sigma_1 \\
&+ 705600\, C_2\, L^{12}\, b^6\, t\, \sigma_1 - 2646000\, C_3\, L^{12}\, b^6\, t\, \sigma_1 + 521850\, C_1\, L^{12}\, b^2\, h^4\, t + 1119300\, C_1\, L^{12}\, b^3\, h^3\, t \\
&+ 1157100\, C_1\, L^{12}\, b^4\, h^2\, t + 113400\, C_2\, L^{12}\, b^2\, h^4\, t - 249200\, C_2\, L^{12}\, b^3\, h^3\, t - 487200\, C_2\, L^{12}\, b^4\, h^2\, t \\
&+ 1212750\, C_3\, L^{12}\, b^2\, h^4\, t + 3454500\, C_3\, L^{12}\, b^3\, h^3\, t + 3969000\, C_3\, L^{12}\, b^4\, h^2\, t + 176400\, C_1\, L^{12}\, b^6\, t\, \sigma_2 \\
&- 705600\, C_2\, L^{12}\, b^6\, t\, \sigma_2 + 2646000\, C_3\, L^{12}\, b^6\, t\, \sigma_2 + 116410\, C_1\, L^{12}\, b\, h^5\, t + 462840\, C_1\, L^{12}\, b^5\, h\, t \\
&+ 109480\, C_2\, L^{12}\, b\, h^5\, t - 194880\, C_2\, L^{12}\, b^5\, h\, t + 143850\, C_3\, L^{12}\, b\, h^5\, t + 1587600\, C_3\, L^{12}\, b^5\, h\, t \\
&+ 4200\, C_1\, L^{12}\, b\, h^5\, t\, \sigma_2 + 529200\, C_1\, L^{12}\, b^5\, h\, t\, \sigma_2 - 16800\, C_2\, L^{12}\, b\, h^5\, t\, \sigma_2 - 2116800\, C_2\, L^{12}\, b^5\, h\, t\, \sigma_2 \\
&+ 63000\, C_3\, L^{12}\, b\, h^5\, t\, \sigma_2 + 7938000\, C_3\, L^{12}\, b^5\, h\, t\, \sigma_2 - 4200\, C_1\, L^{12}\, b\, h^5\, t\, \sigma_1 - 529200\, C_1\, L^{12}\, b^5\, h\, t\, \sigma_1 \\
&+ 16800\, C_2\, L^{12}\, b\, h^5\, t\, \sigma_1 + 2116800\, C_2\, L^{12}\, b^5\, h\, t\, \sigma_1 - 63000\, C_3\, L^{12}\, b\, h^5\, t\, \sigma_1 \\
&- 7938000\, C_3\, L^{12}\, b^5\, h\, t\, \sigma_1 + 63000\, C_1\, L^{12}\, b^2\, h^4\, t\, \sigma_2 + 294000\, C_1\, L^{12}\, b^3\, h^3\, t\, \sigma_2 \\
&+ 588000\, C_1\, L^{12}\, b^4\, h^2\, t\, \sigma_2 - 252000\, C_2\, L^{12}\, b^2\, h^4\, t\, \sigma_2 - 1176000\, C_2\, L^{12}\, b^3\, h^3\, t\, \sigma_2 \\
&- 2352000\, C_2\, L^{12}\, b^4\, h^2\, t\, \sigma_2 + 945000\, C_3\, L^{12}\, b^2\, h^4\, t\, \sigma_2 + 4410000\, C_3\, L^{12}\, b^3\, h^3\, t\, \sigma_2 \\
&+ 8820000\, C_3\, L^{12}\, b^4\, h^2\, t\, \sigma_2 - 63000\, C_1\, L^{12}\, b^2\, h^4\, t\, \sigma_1 - 294000\, C_1\, L^{12}\, b^3\, h^3\, t\, \sigma_1 \\
&- 588000\, C_1\, L^{12}\, b^4\, h^2\, t\, \sigma_1 + 252000\, C_2\, L^{12}\, b^2\, h^4\, t\, \sigma_1 + 1176000\, C_2\, L^{12}\, b^3\, h^3\, t\, \sigma_1 \\
&+ 2352000\, C_2\, L^{12}\, b^4\, h^2\, t\, \sigma_1 - 945000\, C_3\, L^{12}\, b^2\, h^4\, t\, \sigma_1 - 4410000\, C_3\, L^{12}\, b^3\, h^3\, t\, \sigma_1 \\
&- 8820000\, C_3\, L^{12}\, b^4\, h^2\, t\, \sigma_1 \\
a_{h,5} &= 11690\, C_1\, L^{13}\, h^5\, t - 2296\, C_2\, L^{13}\, h^5\, t + 28770\, C_3\, L^{13}\, h^5\, t - 211680\, C_1\, L^{13}\, b^5\, t\, \sigma_1 \\
&+ 846720\, C_2\, L^{13}\, b^5\, t\, \sigma_1 - 3175200\, C_3\, L^{13}\, b^5\, t\, \sigma_1 - 840\, C_1\, L^{13}\, h^5\, t\, \sigma_1 + 3360\, C_2\, L^{13}\, h^5\, t\, \sigma_1 \\
&- 12600\, C_3\, L^{13}\, h^5\, t\, \sigma_1 + 350700\, C_1\, L^{13}\, b^2\, h^3\, t + 487200\, C_1\, L^{13}\, b^3\, h^2\, t - 498960\, C_2\, L^{13}\, b^2\, h^3\, t \\
&- 792960\, C_2\, L^{13}\, b^3\, h^2\, t + 2072700\, C_3\, L^{13}\, b^2\, h^3\, t + 3175200\, C_3\, L^{13}\, b^3\, h^2\, t + 211680\, C_1\, L^{13}\, b^5\, t\, \sigma_2 \\
&- 846720\, C_2\, L^{13}\, b^5\, t\, \sigma_2 + 3175200\, C_3\, L^{13}\, b^5\, t\, \sigma_2 + 840\, C_1\, L^{13}\, h^5\, t\, \sigma_2 - 3360\, C_2\, L^{13}\, h^5\, t\, \sigma_2
\end{aligned}
$$

$$
\begin{aligned}
&+ 12600\, C_3\, L^{13}\, h^5\, t\, \sigma_2 + 107100\, C_1\, L^{13}\, b\, h^4\, t + 243600\, C_1\, L^{13}\, b^4\, h\, t - 102480\, C_2\, L^{13}\, b\, h^4\, t \\
&- 396480\, C_2\, L^{13}\, b^4\, h\, t + 485100\, C_3\, L^{13}\, b\, h^4\, t + 1587600\, C_3\, L^{13}\, b^4\, h\, t + 25200\, C_1\, L^{13}\, b\, h^4\, t\, \sigma_2 \\
&+ 529200\, C_1\, L^{13}\, b^4\, h\, t\, \sigma_2 - 100800\, C_2\, L^{13}\, b\, h^4\, t\, \sigma_2 - 2116800\, C_2\, L^{13}\, b^4\, h\, t\, \sigma_2 \\
&+ 378000\, C_3\, L^{13}\, b\, h^4\, t\, \sigma_2 + 7938000\, C_3\, L^{13}\, b^4\, h\, t\, \sigma_2 - 25200\, C_1\, L^{13}\, b\, h^4\, t\, \sigma_1 \\
&- 529200\, C_1\, L^{13}\, b^4\, h\, t\, \sigma_1 + 100800\, C_2\, L^{13}\, b\, h^4\, t\, \sigma_1 + 2116800\, C_2\, L^{13}\, b^4\, h\, t\, \sigma_1 \\
&- 378000\, C_3\, L^{13}\, b\, h^4\, t\, \sigma_1 - 7938000\, C_3\, L^{13}\, b^4\, h\, t\, \sigma_1 + 176400\, C_1\, L^{13}\, b^2\, h^3\, t\, \sigma_2 \\
&+ 470400\, C_1\, L^{13}\, b^3\, h^2\, t\, \sigma_2 - 705600\, C_2\, L^{13}\, b^2\, h^3\, t\, \sigma_2 - 1881600\, C_2\, L^{13}\, b^3\, h^2\, t\, \sigma_2 \\
&+ 2646000\, C_3\, L^{13}\, b^2\, h^3\, t\, \sigma_2 + 7056000\, C_3\, L^{13}\, b^3\, h^2\, t\, \sigma_2 - 176400\, C_1\, L^{13}\, b^2\, h^3\, t\, \sigma_1 \\
&- 470400\, C_1\, L^{13}\, b^3\, h^2\, t\, \sigma_1 + 705600\, C_2\, L^{13}\, b^2\, h^3\, t\, \sigma_1 + 1881600\, C_2\, L^{13}\, b^3\, h^2\, t\, \sigma_1 \\
&- 2646000\, C_3\, L^{13}\, b^2\, h^3\, t\, \sigma_1 - 7056000\, C_3\, L^{13}\, b^3\, h^2\, t\, \sigma_1 \\
a_{h,4} &= 10570\, C_1\, L^{14}\, h^4\, t - 21560\, C_2\, L^{14}\, h^4\, t + 80850\, C_3\, L^{14}\, h^4\, t - 176400\, C_1\, L^{14}\, b^4\, t\, \sigma_1 \\
&+ 705600\, C_2\, L^{14}\, b^4\, t\, \sigma_1 - 2646000\, C_3\, L^{14}\, b^4\, t\, \sigma_1 - 4200\, C_1\, L^{14}\, h^4\, t\, \sigma_1 + 16800\, C_2\, L^{14}\, h^4\, t\, \sigma_1 \\
&- 63000\, C_3\, L^{14}\, h^4\, t\, \sigma_1 + 153720\, C_1\, L^{14}\, b^2\, h^2\, t - 423360\, C_2\, L^{14}\, b^2\, h^2\, t + 1587600\, C_3\, L^{14}\, b^2\, h^2\, t \\
&+ 176400\, C_1\, L^{14}\, b^4\, t\, \sigma_2 - 705600\, C_2\, L^{14}\, b^4\, t\, \sigma_2 + 2646000\, C_3\, L^{14}\, b^4\, t\, \sigma_2 + 4200\, C_1\, L^{14}\, h^4\, t\, \sigma_2 \\
&- 16800\, C_2\, L^{14}\, h^4\, t\, \sigma_2 + 63000\, C_3\, L^{14}\, h^4\, t\, \sigma_2 + 72380\, C_1\, L^{14}\, b\, h^3\, t + 102480\, C_1\, L^{14}\, b^3\, h\, t \\
&- 184240\, C_2\, L^{14}\, b\, h^3\, t - 282240\, C_2\, L^{14}\, b^3\, h\, t + 690900\, C_3\, L^{14}\, b\, h^3\, t + 1058400\, C_3\, L^{14}\, b^3\, h\, t \\
&+ 58800\, C_1\, L^{14}\, b\, h^3\, t\, \sigma_2 + 352800\, C_1\, L^{14}\, b^3\, h\, t\, \sigma_2 - 235200\, C_2\, L^{14}\, b\, h^3\, t\, \sigma_2 \\
&- 1411200\, C_2\, L^{14}\, b^3\, h\, t\, \sigma_2 + 882000\, C_3\, L^{14}\, b\, h^3\, t\, \sigma_2 + 5292000\, C_3\, L^{14}\, b^3\, h\, t\, \sigma_2 \\
&- 58800\, C_1\, L^{14}\, b\, h^3\, t\, \sigma_1 - 352800\, C_1\, L^{14}\, b^3\, h\, t\, \sigma_1 + 235200\, C_2\, L^{14}\, b\, h^3\, t\, \sigma_1 \\
&+ 1411200\, C_2\, L^{14}\, b^3\, h\, t\, \sigma_1 - 882000\, C_3\, L^{14}\, b\, h^3\, t\, \sigma_1 - 5292000\, C_3\, L^{14}\, b^3\, h\, t\, \sigma_1 \\
&+ 235200\, C_1\, L^{14}\, b^2\, h^2\, t\, \sigma_2 - 940800\, C_2\, L^{14}\, b^2\, h^2\, t\, \sigma_2 + 3528000\, C_3\, L^{14}\, b^2\, h^2\, t\, \sigma_2 \\
&- 235200\, C_1\, L^{14}\, b^2\, h^2\, t\, \sigma_1 + 940800\, C_2\, L^{14}\, b^2\, h^2\, t\, \sigma_1 - 3528000\, C_3\, L^{14}\, b^2\, h^2\, t\, \sigma_1 \\
a_{h,3} &= 7700\, C_1\, L^{15}\, h^3\, t - 26320\, C_2\, L^{15}\, h^3\, t + 98700\, C_3\, L^{15}\, h^3\, t - 100800\, C_1\, L^{15}\, b^3\, t\, \sigma_1 \\
&+ 403200\, C_2\, L^{15}\, b^3\, t\, \sigma_1 - 1512000\, C_3\, L^{15}\, b^3\, t\, \sigma_1 - 8400\, C_1\, L^{15}\, h^3\, t\, \sigma_1 + 33600\, C_2\, L^{15}\, h^3\, t\, \sigma_1 \\
&- 126000\, C_3\, L^{15}\, h^3\, t\, \sigma_1 + 100800\, C_1\, L^{15}\, b^3\, t\, \sigma_2 - 403200\, C_2\, L^{15}\, b^3\, t\, \sigma_2 + 1512000\, C_3\, L^{15}\, b^3\, t\, \sigma_2 \\
&+ 8400\, C_1\, L^{15}\, h^3\, t\, \sigma_2 - 33600\, C_2\, L^{15}\, h^3\, t\, \sigma_2 + 126000\, C_3\, L^{15}\, h^3\, t\, \sigma_2 + 33600\, C_1\, L^{15}\, b\, h^2\, t \\
&+ 33600\, C_1\, L^{15}\, b^2\, h\, t - 120960\, C_2\, L^{15}\, b\, h^2\, t - 120960\, C_2\, L^{15}\, b^2\, h\, t + 453600\, C_3\, L^{15}\, b\, h^2\, t \\
&+ 453600\, C_3\, L^{15}\, b^2\, h\, t + 67200\, C_1\, L^{15}\, b\, h^2\, t\, \sigma_2 + 151200\, C_1\, L^{15}\, b^2\, h\, t\, \sigma_2 \\
&- 268800\, C_2\, L^{15}\, b\, h^2\, t\, \sigma_2 - 604800\, C_2\, L^{15}\, b^2\, h\, t\, \sigma_2 + 1008000\, C_3\, L^{15}\, b\, h^2\, t\, \sigma_2 \\
&+ 2268000\, C_3\, L^{15}\, b^2\, h\, t\, \sigma_2 - 67200\, C_1\, L^{15}\, b\, h^2\, t\, \sigma_1 - 151200\, C_1\, L^{15}\, b^2\, h\, t\, \sigma_1 \\
&+ 268800\, C_2\, L^{15}\, b\, h^2\, t\, \sigma_1 + 604800\, C_2\, L^{15}\, b^2\, h\, t\, \sigma_1 - 1008000\, C_3\, L^{15}\, b\, h^2\, t\, \sigma_1 \\
&- 2268000\, C_3\, L^{15}\, b^2\, h\, t\, \sigma_1 \\
a_{h,2} &= 3780\, C_1\, L^{16}\, h^2\, t - 15120\, C_2\, L^{16}\, h^2\, t + 56700\, C_3\, L^{16}\, h^2\, t - 37800\, C_1\, L^{16}\, b^2\, t\, \sigma_1 \\
&+ 151200\, C_2\, L^{16}\, b^2\, t\, \sigma_1 - 567000\, C_3\, L^{16}\, b^2\, t\, \sigma_1 - 8400\, C_1\, L^{16}\, h^2\, t\, \sigma_1 + 33600\, C_2\, L^{16}\, h^2\, t\, \sigma_1
\end{aligned}
$$

$$
\begin{aligned}
&- 126000\, C_3\, L^{16}\, h^2\, t\, \sigma_1 + 7560\, C_1\, L^{16}\, b\, h\, t - 30240\, C_2\, L^{16}\, b\, h\, t + 113400\, C_3\, L^{16}\, b\, h\, t \\
&+ 37800\, C_1\, L^{16}\, b^2\, t\, \sigma_2 - 151200\, C_2\, L^{16}\, b^2\, t\, \sigma_2 + 567000\, C_3\, L^{16}\, b^2\, t\, \sigma_2 + 8400\, C_1\, L^{16}\, h^2\, t\, \sigma_2 \\
&- 33600\, C_2\, L^{16}\, h^2\, t\, \sigma_2 + 126000\, C_3\, L^{16}\, h^2\, t\, \sigma_2 - 37800\, C_1\, L^{16}\, b\, h\, t\, \sigma_1 + 151200\, C_2\, L^{16}\, b\, h\, t\, \sigma_1 \\
&- 567000\, C_3\, L^{16}\, b\, h\, t\, \sigma_1 + 37800\, C_1\, L^{16}\, b\, h\, t\, \sigma_2 - 151200\, C_2\, L^{16}\, b\, h\, t\, \sigma_2 + 567000\, C_3\, L^{16}\, b\, h\, t\, \sigma_2 \\
a_{h,1} &= 840\, C_1\, L^{17}\, h\, t - 3360\, C_2\, L^{17}\, h\, t + 12600\, C_3\, L^{17}\, h\, t + 8400\, C_1\, L^{17}\, b\, t\, \sigma_2 - 33600\, C_2\, L^{17}\, b\, t\, \sigma_2 \\
&+ 126000\, C_3\, L^{17}\, b\, t\, \sigma_2 + 4200\, C_1\, L^{17}\, h\, t\, \sigma_2 - 16800\, C_2\, L^{17}\, h\, t\, \sigma_2 + 63000\, C_3\, L^{17}\, h\, t\, \sigma_2 \\
&- 8400\, C_1\, L^{17}\, b\, t\, \sigma_1 + 33600\, C_2\, L^{17}\, b\, t\, \sigma_1 - 126000\, C_3\, L^{17}\, b\, t\, \sigma_1 - 4200\, C_1\, L^{17}\, h\, t\, \sigma_1 \\
&+ 16800\, C_2\, L^{17}\, h\, t\, \sigma_1 - 63000\, C_3\, L^{17}\, h\, t\, \sigma_1 \\
a_{h,0} &= 840\, C_1\, L^{18}\, t\, \sigma_2 - 3360\, C_2\, L^{18}\, t\, \sigma_2 + 12600\, C_3\, L^{18}\, t\, \sigma_2 - 840\, C_1\, L^{18}\, t\, \sigma_1 + 3360\, C_2\, L^{18}\, t\, \sigma_1 \\
&- 12600\, C_3\, L^{18}\, t\, \sigma_1 \\
b_{h,12} &= 210\, L^6\, b^{10} + 1050\, L^6\, b^9\, h + 2100\, L^6\, b^8\, h^2 + 2100\, L^6\, b^7\, h^3 + 1050\, L^6\, b^6\, h^4 + 210\, L^6\, b^5\, h^5 \\
b_{h,11} &= 2100\, L^7\, b^9 + 9450\, L^7\, b^8\, h + 16800\, L^7\, b^7\, h^2 + 14700\, L^7\, b^6\, h^3 + 6300\, L^7\, b^5\, h^4 + 1050\, L^7\, b^4\, h^5 \\
b_{h,10} &= 9450\, L^8\, b^8 + 37800\, L^8\, b^7\, h + 58800\, L^8\, b^6\, h^2 + 44100\, L^8\, b^5\, h^3 + 15750\, L^8\, b^4\, h^4 + 2100\, L^8\, b^3\, h^5 \\
b_{h,9} &= 25200\, L^9\, b^7 + 88200\, L^9\, b^6\, h + 117600\, L^9\, b^5\, h^2 + 73500\, L^9\, b^4\, h^3 + 21000\, L^9\, b^3\, h^4 \\
&+ 2100\, L^9\, b^2\, h^5 \\
b_{h,8} &= 44100\, L^{10}\, b^6 + 132300\, L^{10}\, b^5\, h + 147000\, L^{10}\, b^4\, h^2 + 73500\, L^{10}\, b^3\, h^3 + 15750\, L^{10}\, b^2\, h^4 \\
&+ 1050\, L^{10}\, b\, h^5 \\
b_{h,7} &= 52920\, L^{11}\, b^5 + 132300\, L^{11}\, b^4\, h + 117600\, L^{11}\, b^3\, h^2 + 44100\, L^{11}\, b^2\, h^3 + 6300\, L^{11}\, b\, h^4 \\
&+ 210\, L^{11}\, h^5 \\
b_{h,6} &= 44100\, L^{12}\, b^4 + 88200\, L^{12}\, b^3\, h + 58800\, L^{12}\, b^2\, h^2 + 14700\, L^{12}\, b\, h^3 + 1050\, L^{12}\, h^4 \\
b_{h,5} &= 25200\, L^{13}\, b^3 + 37800\, L^{13}\, b^2\, h + 16800\, L^{13}\, b\, h^2 + 2100\, L^{13}\, h^3 \\
b_{h,4} &= 9450\, L^{14}\, b^2 + 9450\, L^{14}\, b\, h + 2100\, L^{14}\, h^2 \\
b_{h,3} &= 2100\, L^{15}\, b + 1050\, L^{15}\, h \\
b_{h,2} &= 210\, L^{16}
\end{aligned}
$$

where
$\sigma_1 = \log(L + b\, q + h\, q) \qquad \sigma_2 = \log(L + b\, q)$

$\mathrm{a}_{t,13} = 2016\, C_3\, L\, \pi\, b^{14}\, r_0\, t + 1008\, C_3\, L\, \pi\, b^{14}\, t^2 + 28224\, C_3\, L\, \pi\, b^{13}\, h\, r_0\, t + 14112\, C_3\, L\, \pi\, b^{13}\, h\, t^2 + 3312\, C_3\, L\, \pi^2\, b^{13}\, {r_0}^2\, t + 3312\, C_3\, L\, \pi^2\, b^{13}\, r_0\, t^2 + 1104\, C_3\, L\, \pi^2\, b^{13}\, t^3 + 183456\, C_3\, L\, \pi\, b^{12}\, h^2\, r_0\, t + 91728\, C_3\, L\, \pi\, b^{12}\, h^2\, t^2 + 43056\, C_3\, L\, \pi^2\, b^{12}\, h\, {r_0}^2\, t + 43056\, C_3\, L\, \pi^2\, b^{12}\, h\, r_0\, t^2 + 14352\, C_3\, L\, \pi^2\, b^{12}\, h\, t^3 + 3096\, C_3\, L\, \pi^3\, b^{12}\, {r_0}^3\, t + 4644\, C_3\, L\, \pi^3\, b^{12}\, {r_0}^2\, t^2 + 3096\, C_3\, L\, \pi^3\, b^{12}\, r_0\, t^3 + 774\, C_3\, L\, \pi^3\, b^{12}\, t^4 + 733824\, C_3\, L\, \pi\, b^{11}\, h^3\, r_0\, t + 366912\, C_3\, L\, \pi\, b^{11}\, h^3\, t^2 + 258336\, C_3\, L\, \pi^2\, b^{11}\, h^2\, {r_0}^2\, t + 258336\, C_3\, L\, \pi^2\, b^{11}\, h^2\, r_0\, t^2 + 86112\, C_3\, L\, \pi^2\, b^{11}\, h^2\, t^3 + 37152\, C_3\, L\, \pi^3\, b^{11}\, h\, {r_0}^3\, t + 55728\, C_3\, L\, \pi^3\, b^{11}\, h\, {r_0}^2\, t^2 + 37152\, C_3\, L\, \pi^3\, b^{11}\, h\, r_0\, t^3 + 9288\, C_3\, L\, \pi^3\, b^{11}\, h\, t^4 + 2018016\, C_3\, L\, \pi\, b^{10}\, h^4\, r_0\, t +$

$1009008\,C_3\,L\,\pi\,b^{10}\,h^4\,t^2+947232\,C_3\,L\,\pi^2\,b^{10}\,h^3\,{r_0}^2\,t+947232\,C_3\,L\,\pi^2\,b^{10}\,h^3\,r_0\,t^2+$
$315744\,C_3\,L\,\pi^2\,b^{10}\,h^3\,t^3+204336\,C_3\,L\,\pi^3\,b^{10}\,h^2\,{r_0}^3\,t+306504\,C_3\,L\,\pi^3\,b^{10}\,h^2\,{r_0}^2\,t^2+$
$204336\,C_3\,L\,\pi^3\,b^{10}\,h^2\,r_0\,t^3+51084\,C_3\,L\,\pi^3\,b^{10}\,h^2\,t^4+4036032\,C_3\,L\,\pi\,b^9\,h^5\,r_0\,t+$
$2018016\,C_3\,L\,\pi\,b^9\,h^5\,t^2+2368080\,C_3\,L\,\pi^2\,b^9\,h^4\,{r_0}^2\,t+2368080\,C_3\,L\,\pi^2\,b^9\,h^4\,r_0\,t^2+$
$789360\,C_3\,L\,\pi^2\,b^9\,h^4\,t^3+681120\,C_3\,L\,\pi^3\,b^9\,h^3\,{r_0}^3\,t+1021680\,C_3\,L\,\pi^3\,b^9\,h^3\,{r_0}^2\,t^2+$
$681120\,C_3\,L\,\pi^3\,b^9\,h^3\,r_0\,t^3+170280\,C_3\,L\,\pi^3\,b^9\,h^3\,t^4+6054048\,C_3\,L\,\pi\,b^8\,h^6\,r_0\,t+$
$3027024\,C_3\,L\,\pi\,b^8\,h^6\,t^2+4262544\,C_3\,L\,\pi^2\,b^8\,h^5\,{r_0}^2\,t+4262544\,C_3\,L\,\pi^2\,b^8\,h^5\,r_0\,t^2+$
$1420848\,C_3\,L\,\pi^2\,b^8\,h^5\,t^3+1532520\,C_3\,L\,\pi^3\,b^8\,h^4\,{r_0}^3\,t+2298780\,C_3\,L\,\pi^3\,b^8\,h^4\,{r_0}^2\,t^2+$
$1532520\,C_3\,L\,\pi^3\,b^8\,h^4\,r_0\,t^3+383130\,C_3\,L\,\pi^3\,b^8\,h^4\,t^4+6918912\,C_3\,L\,\pi\,b^7\,h^7\,r_0\,t+$
$3459456\,C_3\,L\,\pi\,b^7\,h^7\,t^2+5683392\,C_3\,L\,\pi^2\,b^7\,h^6\,{r_0}^2\,t+5683392\,C_3\,L\,\pi^2\,b^7\,h^6\,r_0\,t^2+$
$1894464\,C_3\,L\,\pi^2\,b^7\,h^6\,t^3+2452032\,C_3\,L\,\pi^3\,b^7\,h^5\,{r_0}^3\,t+3678048\,C_3\,L\,\pi^3\,b^7\,h^5\,{r_0}^2\,t^2+$
$2452032\,C_3\,L\,\pi^3\,b^7\,h^5\,r_0\,t^3+613008\,C_3\,L\,\pi^3\,b^7\,h^5\,t^4+6054048\,C_3\,L\,\pi\,b^6\,h^8\,r_0\,t+$
$3027024\,C_3\,L\,\pi\,b^6\,h^8\,t^2+5683392\,C_3\,L\,\pi^2\,b^6\,h^7\,{r_0}^2\,t+5683392\,C_3\,L\,\pi^2\,b^6\,h^7\,r_0\,t^2+$
$1894464\,C_3\,L\,\pi^2\,b^6\,h^7\,t^3+2860704\,C_3\,L\,\pi^3\,b^6\,h^6\,{r_0}^3\,t+4291056\,C_3\,L\,\pi^3\,b^6\,h^6\,{r_0}^2\,t^2+$
$2860704\,C_3\,L\,\pi^3\,b^6\,h^6\,r_0\,t^3+715176\,C_3\,L\,\pi^3\,b^6\,h^6\,t^4+4036032\,C_3\,L\,\pi\,b^5\,h^9\,r_0\,t+$
$2018016\,C_3\,L\,\pi\,b^5\,h^9\,t^2+4262544\,C_3\,L\,\pi^2\,b^5\,h^8\,{r_0}^2\,t+4262544\,C_3\,L\,\pi^2\,b^5\,h^8\,r_0\,t^2+$
$1420848\,C_3\,L\,\pi^2\,b^5\,h^8\,t^3+2452032\,C_3\,L\,\pi^3\,b^5\,h^7\,{r_0}^3\,t+3678048\,C_3\,L\,\pi^3\,b^5\,h^7\,{r_0}^2\,t^2+$
$2452032\,C_3\,L\,\pi^3\,b^5\,h^7\,r_0\,t^3+613008\,C_3\,L\,\pi^3\,b^5\,h^7\,t^4+2018016\,C_3\,L\,\pi\,b^4\,h^{10}\,r_0\,t+$
$1009008\,C_3\,L\,\pi\,b^4\,h^{10}\,t^2+2368080\,C_3\,L\,\pi^2\,b^4\,h^9\,{r_0}^2\,t+2368080\,C_3\,L\,\pi^2\,b^4\,h^9\,r_0\,t^2+$
$789360\,C_3\,L\,\pi^2\,b^4\,h^9\,t^3+1532520\,C_3\,L\,\pi^3\,b^4\,h^8\,{r_0}^3\,t+2298780\,C_3\,L\,\pi^3\,b^4\,h^8\,{r_0}^2\,t^2+$
$1532520\,C_3\,L\,\pi^3\,b^4\,h^8\,r_0\,t^3+383130\,C_3\,L\,\pi^3\,b^4\,h^8\,t^4+733824\,C_3\,L\,\pi\,b^3\,h^{11}\,r_0\,t+$
$366912\,C_3\,L\,\pi\,b^3\,h^{11}\,t^2+947232\,C_3\,L\,\pi^2\,b^3\,h^{10}\,{r_0}^2\,t+947232\,C_3\,L\,\pi^2\,b^3\,h^{10}\,r_0\,t^2+$
$315744\,C_3\,L\,\pi^2\,b^3\,h^{10}\,t^3+681120\,C_3\,L\,\pi^3\,b^3\,h^9\,{r_0}^3\,t+1021680\,C_3\,L\,\pi^3\,b^3\,h^9\,{r_0}^2\,t^2+$
$681120\,C_3\,L\,\pi^3\,b^3\,h^9\,r_0\,t^3+170280\,C_3\,L\,\pi^3\,b^3\,h^9\,t^4+183456\,C_3\,L\,\pi\,b^2\,h^{12}\,r_0\,t+$
$91728\,C_3\,L\,\pi\,b^2\,h^{12}\,t^2+258336\,C_3\,L\,\pi^2\,b^2\,h^{11}\,{r_0}^2\,t+258336\,C_3\,L\,\pi^2\,b^2\,h^{11}\,r_0\,t^2+$
$86112\,C_3\,L\,\pi^2\,b^2\,h^{11}\,t^3+204336\,C_3\,L\,\pi^3\,b^2\,h^{10}\,{r_0}^3\,t+306504\,C_3\,L\,\pi^3\,b^2\,h^{10}\,{r_0}^2\,t^2+$
$204336\,C_3\,L\,\pi^3\,b^2\,h^{10}\,r_0\,t^3+51084\,C_3\,L\,\pi^3\,b^2\,h^{10}\,t^4+28224\,C_3\,L\,\pi\,b\,h^{13}\,r_0\,t+14112\,C_3\,L\,\pi\,b\,h^{13}\,t^2+$
$43056\,C_3\,L\,\pi^2\,b\,h^{12}\,{r_0}^2\,t+43056\,C_3\,L\,\pi^2\,b\,h^{12}\,r_0\,t^2+14352\,C_3\,L\,\pi^2\,b\,h^{12}\,t^3+37152\,C_3\,L\,\pi^3\,b\,h^{11}\,{r_0}^3\,t+$
$55728\,C_3\,L\,\pi^3\,b\,h^{11}\,{r_0}^2\,t^2+37152\,C_3\,L\,\pi^3\,b\,h^{11}\,r_0\,t^3+9288\,C_3\,L\,\pi^3\,b\,h^{11}\,t^4+2016\,C_3\,L\,\pi\,h^{14}\,r_0\,t+$
$1008\,C_3\,L\,\pi\,h^{14}\,t^2+3312\,C_3\,L\,\pi^2\,h^{13}\,{r_0}^2\,t+3312\,C_3\,L\,\pi^2\,h^{13}\,r_0\,t^2+1104\,C_3\,L\,\pi^2\,h^{13}\,t^3+$
$3096\,C_3\,L\,\pi^3\,h^{12}\,{r_0}^3\,t+4644\,C_3\,L\,\pi^3\,h^{12}\,{r_0}^2\,t^2+3096\,C_3\,L\,\pi^3\,h^{12}\,r_0\,t^3+774\,C_3\,L\,\pi^3\,h^{12}\,t^4$

$\mathrm{a}_{t,12}=48\,\pi\,C_2\,L^2\,b^{13}\,t^2+6264\,\pi\,C_3\,L^2\,b^{13}\,t^2+48\,\pi\,C_2\,L^2\,h^{13}\,t^2+6264\,\pi\,C_3\,L^2\,h^{13}\,t^2+$
$40\,C_2\,L^2\,b^{12}\,t^3\,\pi^2+20\,C_2\,L^2\,b^{11}\,t^4\,\pi^3+6468\,C_3\,L^2\,b^{12}\,t^3\,\pi^2+4254\,C_3\,L^2\,b^{11}\,t^4\,\pi^3+$
$40\,C_2\,L^2\,h^{12}\,t^3\,\pi^2+20\,C_2\,L^2\,h^{11}\,t^4\,\pi^3+6468\,C_3\,L^2\,h^{12}\,t^3\,\pi^2+4254\,C_3\,L^2\,h^{11}\,t^4\,\pi^3+$
$120\,C_2\,L^2\,b^{12}\,r_0\,t^2\,\pi^2+120\,C_2\,L^2\,b^{12}\,{r_0}^2\,t\,\pi^2+80\,C_2\,L^2\,b^{11}\,r_0\,t^3\,\pi^3+80\,C_2\,L^2\,b^{11}\,{r_0}^3\,t\,\pi^3+$
$19404\,C_3\,L^2\,b^{12}\,r_0\,t^2\,\pi^2+19404\,C_3\,L^2\,b^{12}\,{r_0}^2\,t\,\pi^2+17016\,C_3\,L^2\,b^{11}\,r_0\,t^3\,\pi^3+17016\,C_3\,L^2\,b^{11}\,{r_0}^3\,t\,\pi^3+$
$120\,C_2\,L^2\,h^{12}\,r_0\,t^2\,\pi^2+120\,C_2\,L^2\,h^{12}\,{r_0}^2\,t\,\pi^2+80\,C_2\,L^2\,h^{11}\,r_0\,t^3\,\pi^3+80\,C_2\,L^2\,h^{11}\,{r_0}^3\,t\,\pi^3+$
$19404\,C_3\,L^2\,h^{12}\,r_0\,t^2\,\pi^2+19404\,C_3\,L^2\,h^{12}\,{r_0}^2\,t\,\pi^2+17016\,C_3\,L^2\,h^{11}\,r_0\,t^3\,\pi^3+17016\,C_3\,L^2\,h^{11}\,{r_0}^3\,t\,\pi^3+$
$96\,\pi\,C_2\,L^2\,b^{13}\,r_0\,t+12528\,\pi\,C_3\,L^2\,b^{13}\,r_0\,t+96\,\pi\,C_2\,L^2\,h^{13}\,r_0\,t+12528\,\pi\,C_3\,L^2\,h^{13}\,r_0\,t+$
$2640\,C_2\,L^2\,b^2\,h^{10}\,t^3\,\pi^2+8800\,C_2\,L^2\,b^3\,h^9\,t^3\,\pi^2+19800\,C_2\,L^2\,b^4\,h^8\,t^3\,\pi^2+31680\,C_2\,L^2\,b^5\,h^7\,t^3\,\pi^2+$
$36960\,C_2\,L^2\,b^6\,h^6\,t^3\,\pi^2+31680\,C_2\,L^2\,b^7\,h^5\,t^3\,\pi^2+19800\,C_2\,L^2\,b^8\,h^4\,t^3\,\pi^2+8800\,C_2\,L^2\,b^9\,h^3\,t^3\,\pi^2+$
$2640\,C_2\,L^2\,b^{10}\,h^2\,t^3\,\pi^2+1100\,C_2\,L^2\,b^2\,h^9\,t^4\,\pi^3+3300\,C_2\,L^2\,b^3\,h^8\,t^4\,\pi^3+6600\,C_2\,L^2\,b^4\,h^7\,t^4\,\pi^3+$
$9240\,C_2\,L^2\,b^5\,h^6\,t^4\,\pi^3+9240\,C_2\,L^2\,b^6\,h^5\,t^4\,\pi^3+6600\,C_2\,L^2\,b^7\,h^4\,t^4\,\pi^3+3300\,C_2\,L^2\,b^8\,h^3\,t^4\,\pi^3+$
$1100\,C_2\,L^2\,b^9\,h^2\,t^4\,\pi^3+426888\,C_3\,L^2\,b^2\,h^{10}\,t^3\,\pi^2+1422960\,C_3\,L^2\,b^3\,h^9\,t^3\,\pi^2+3201660\,C_3\,L^2\,b^4\,h^8\,t^3\,\pi^2+$
$5122656\,C_3\,L^2\,b^5\,h^7\,t^3\,\pi^2+5976432\,C_3\,L^2\,b^6\,h^6\,t^3\,\pi^2+5122656\,C_3\,L^2\,b^7\,h^5\,t^3\,\pi^2+$

$3201660\,C_3\,L^2\,b^8\,h^4\,t^3\,\pi^2+1422960\,C_3\,L^2\,b^9\,h^3\,t^3\,\pi^2+426888\,C_3\,L^2\,b^{10}\,h^2\,t^3\,\pi^2+$
$233970\,C_3\,L^2\,b^2\,h^9\,t^4\,\pi^3+701910\,C_3\,L^2\,b^3\,h^8\,t^4\,\pi^3+1403820\,C_3\,L^2\,b^4\,h^7\,t^4\,\pi^3+$
$1965348\,C_3\,L^2\,b^5\,h^6\,t^4\,\pi^3+1965348\,C_3\,L^2\,b^6\,h^5\,t^4\,\pi^3+1403820\,C_3\,L^2\,b^7\,h^4\,t^4\,\pi^3+$
$701910\,C_3\,L^2\,b^8\,h^3\,t^4\,\pi^3+233970\,C_3\,L^2\,b^9\,h^2\,t^4\,\pi^3+120\,C_2\,L^2\,b^{11}\,{r_0}^2\,t^2\,\pi^3+25524\,C_3\,L^2\,b^{11}\,{r_0}^2\,t^2\,\pi^3+$
$120\,C_2\,L^2\,h^{11}\,{r_0}^2\,t^2\,\pi^3+25524\,C_3\,L^2\,h^{11}\,{r_0}^2\,t^2\,\pi^3+624\,\pi\,C_2\,L^2\,b\,h^{12}\,t^2+624\,\pi\,C_2\,L^2\,b^{12}\,h\,t^2+$
$81432\,\pi\,C_3\,L^2\,b\,h^{12}\,t^2+81432\,\pi\,C_3\,L^2\,b^{12}\,h\,t^2+3744\,\pi\,C_2\,L^2\,b^2\,h^{11}\,t^2+13728\,\pi\,C_2\,L^2\,b^3\,h^{10}\,t^2+$
$34320\,\pi\,C_2\,L^2\,b^4\,h^9\,t^2+61776\,\pi\,C_2\,L^2\,b^5\,h^8\,t^2+82368\,\pi\,C_2\,L^2\,b^6\,h^7\,t^2+82368\,\pi\,C_2\,L^2\,b^7\,h^6\,t^2+$
$61776\,\pi\,C_2\,L^2\,b^8\,h^5\,t^2+34320\,\pi\,C_2\,L^2\,b^9\,h^4\,t^2+13728\,\pi\,C_2\,L^2\,b^{10}\,h^3\,t^2+3744\,\pi\,C_2\,L^2\,b^{11}\,h^2\,t^2+$
$480\,C_2\,L^2\,b\,h^{11}\,t^3\,\pi^2+480\,C_2\,L^2\,b^{11}\,h\,t^3\,\pi^2+488592\,\pi\,C_3\,L^2\,b^2\,h^{11}\,t^2+1791504\,\pi\,C_3\,L^2\,b^3\,h^{10}\,t^2+$
$4478760\,\pi\,C_3\,L^2\,b^4\,h^9\,t^2+8061768\,\pi\,C_3\,L^2\,b^5\,h^8\,t^2+10749024\,\pi\,C_3\,L^2\,b^6\,h^7\,t^2+$
$10749024\,\pi\,C_3\,L^2\,b^7\,h^6\,t^2+8061768\,\pi\,C_3\,L^2\,b^8\,h^5\,t^2+4478760\,\pi\,C_3\,L^2\,b^9\,h^4\,t^2+$
$1791504\,\pi\,C_3\,L^2\,b^{10}\,h^3\,t^2+488592\,\pi\,C_3\,L^2\,b^{11}\,h^2\,t^2+220\,C_2\,L^2\,b\,h^{10}\,t^4\,\pi^3+220\,C_2\,L^2\,b^{10}\,h\,t^4\,\pi^3+$
$77616\,C_3\,L^2\,b\,h^{11}\,t^3\,\pi^2+77616\,C_3\,L^2\,b^{11}\,h\,t^3\,\pi^2+46794\,C_3\,L^2\,b\,h^{10}\,t^4\,\pi^3+46794\,C_3\,L^2\,b^{10}\,h\,t^4\,\pi^3+$
$7488\,\pi\,C_2\,L^2\,b^2\,h^{11}\,r_0\,t+27456\,\pi\,C_2\,L^2\,b^3\,h^{10}\,r_0\,t+68640\,\pi\,C_2\,L^2\,b^4\,h^9\,r_0\,t+123552\,\pi\,C_2\,L^2\,b^5\,h^8\,r_0\,t+$
$164736\,\pi\,C_2\,L^2\,b^6\,h^7\,r_0\,t+164736\,\pi\,C_2\,L^2\,b^7\,h^6\,r_0\,t+123552\,\pi\,C_2\,L^2\,b^8\,h^5\,r_0\,t+$
$68640\,\pi\,C_2\,L^2\,b^9\,h^4\,r_0\,t+27456\,\pi\,C_2\,L^2\,b^{10}\,h^3\,r_0\,t+7488\,\pi\,C_2\,L^2\,b^{11}\,h^2\,r_0\,t+977184\,\pi\,C_3\,L^2\,b^2\,h^{11}\,r_0\,t+$
$3583008\,\pi\,C_3\,L^2\,b^3\,h^{10}\,r_0\,t+8957520\,\pi\,C_3\,L^2\,b^4\,h^9\,r_0\,t+16123536\,\pi\,C_3\,L^2\,b^5\,h^8\,r_0\,t+$
$21498048\,\pi\,C_3\,L^2\,b^6\,h^7\,r_0\,t+21498048\,\pi\,C_3\,L^2\,b^7\,h^6\,r_0\,t+16123536\,\pi\,C_3\,L^2\,b^8\,h^5\,r_0\,t+$
$8957520\,\pi\,C_3\,L^2\,b^9\,h^4\,r_0\,t+3583008\,\pi\,C_3\,L^2\,b^{10}\,h^3\,r_0\,t+977184\,\pi\,C_3\,L^2\,b^{11}\,h^2\,r_0\,t+$
$6600\,C_2\,L^2\,b^2\,h^9\,{r_0}^2\,t^2\,\pi^3+19800\,C_2\,L^2\,b^3\,h^8\,{r_0}^2\,t^2\,\pi^3+39600\,C_2\,L^2\,b^4\,h^7\,{r_0}^2\,t^2\,\pi^3+$
$55440\,C_2\,L^2\,b^5\,h^6\,{r_0}^2\,t^2\,\pi^3+55440\,C_2\,L^2\,b^6\,h^5\,{r_0}^2\,t^2\,\pi^3+39600\,C_2\,L^2\,b^7\,h^4\,{r_0}^2\,t^2\,\pi^3+$
$19800\,C_2\,L^2\,b^8\,h^3\,{r_0}^2\,t^2\,\pi^3+6600\,C_2\,L^2\,b^9\,h^2\,{r_0}^2\,t^2\,\pi^3+1403820\,C_3\,L^2\,b^2\,h^9\,{r_0}^2\,t^2\,\pi^3+$
$4211460\,C_3\,L^2\,b^3\,h^8\,{r_0}^2\,t^2\,\pi^3+8422920\,C_3\,L^2\,b^4\,h^7\,{r_0}^2\,t^2\,\pi^3+11792088\,C_3\,L^2\,b^5\,h^6\,{r_0}^2\,t^2\,\pi^3+$
$11792088\,C_3\,L^2\,b^6\,h^5\,{r_0}^2\,t^2\,\pi^3+8422920\,C_3\,L^2\,b^7\,h^4\,{r_0}^2\,t^2\,\pi^3+4211460\,C_3\,L^2\,b^8\,h^3\,{r_0}^2\,t^2\,\pi^3+$
$1403820\,C_3\,L^2\,b^9\,h^2\,{r_0}^2\,t^2\,\pi^3+1440\,C_2\,L^2\,b\,h^{11}\,r_0\,t^2\,\pi^2+1440\,C_2\,L^2\,b\,h^{11}\,{r_0}^2\,t\,\pi^2+$
$1440\,C_2\,L^2\,b^{11}\,h\,r_0\,t^2\,\pi^2+1440\,C_2\,L^2\,b^{11}\,h\,{r_0}^2\,t\,\pi^2+880\,C_2\,L^2\,b\,h^{10}\,r_0\,t^3\,\pi^3+880\,C_2\,L^2\,b\,h^{10}\,{r_0}^3\,t\,\pi^3+$
$880\,C_2\,L^2\,b^{10}\,h\,r_0\,t^3\,\pi^3+880\,C_2\,L^2\,b^{10}\,h\,{r_0}^3\,t\,\pi^3+232848\,C_3\,L^2\,b\,h^{11}\,r_0\,t^2\,\pi^2+232848\,C_3\,L^2\,b\,h^{11}\,{r_0}^2\,t\,\pi^2+$
$232848\,C_3\,L^2\,b^{11}\,h\,r_0\,t^2\,\pi^2+232848\,C_3\,L^2\,b^{11}\,h\,{r_0}^2\,t\,\pi^2+187176\,C_3\,L^2\,b\,h^{10}\,r_0\,t^3\,\pi^3+$
$187176\,C_3\,L^2\,b\,h^{10}\,{r_0}^3\,t\,\pi^3+187176\,C_3\,L^2\,b^{10}\,h\,r_0\,t^3\,\pi^3+187176\,C_3\,L^2\,b^{10}\,h\,{r_0}^3\,t\,\pi^3+$
$1248\,\pi\,C_2\,L^2\,b\,h^{12}\,r_0\,t+1248\,\pi\,C_2\,L^2\,b^{12}\,h\,r_0\,t+162864\,\pi\,C_3\,L^2\,b\,h^{12}\,r_0\,t+162864\,\pi\,C_3\,L^2\,b^{12}\,h\,r_0\,t+$
$7920\,C_2\,L^2\,b^2\,h^{10}\,r_0\,t^2\,\pi^2+7920\,C_2\,L^2\,b^2\,h^{10}\,{r_0}^2\,t\,\pi^2+26400\,C_2\,L^2\,b^3\,h^9\,r_0\,t^2\,\pi^2+$
$26400\,C_2\,L^2\,b^3\,h^9\,{r_0}^2\,t\,\pi^2+59400\,C_2\,L^2\,b^4\,h^8\,r_0\,t^2\,\pi^2+59400\,C_2\,L^2\,b^4\,h^8\,{r_0}^2\,t\,\pi^2+$
$95040\,C_2\,L^2\,b^5\,h^7\,r_0\,t^2\,\pi^2+95040\,C_2\,L^2\,b^5\,h^7\,{r_0}^2\,t\,\pi^2+110880\,C_2\,L^2\,b^6\,h^6\,r_0\,t^2\,\pi^2+$
$110880\,C_2\,L^2\,b^6\,h^6\,{r_0}^2\,t\,\pi^2+95040\,C_2\,L^2\,b^7\,h^5\,r_0\,t^2\,\pi^2+95040\,C_2\,L^2\,b^7\,h^5\,{r_0}^2\,t\,\pi^2+$
$59400\,C_2\,L^2\,b^8\,h^4\,r_0\,t^2\,\pi^2+59400\,C_2\,L^2\,b^8\,h^4\,{r_0}^2\,t\,\pi^2+26400\,C_2\,L^2\,b^9\,h^3\,r_0\,t^2\,\pi^2+$
$26400\,C_2\,L^2\,b^9\,h^3\,{r_0}^2\,t\,\pi^2+7920\,C_2\,L^2\,b^{10}\,h^2\,r_0\,t^2\,\pi^2+7920\,C_2\,L^2\,b^{10}\,h^2\,{r_0}^2\,t\,\pi^2+$
$1320\,C_2\,L^2\,b\,h^{10}\,{r_0}^2\,t^2\,\pi^3+4400\,C_2\,L^2\,b^2\,h^9\,r_0\,t^3\,\pi^3+4400\,C_2\,L^2\,b^2\,h^9\,{r_0}^3\,t\,\pi^3+$
$13200\,C_2\,L^2\,b^3\,h^8\,r_0\,t^3\,\pi^3+13200\,C_2\,L^2\,b^3\,h^8\,{r_0}^3\,t\,\pi^3+26400\,C_2\,L^2\,b^4\,h^7\,r_0\,t^3\,\pi^3+$
$26400\,C_2\,L^2\,b^4\,h^7\,{r_0}^3\,t\,\pi^3+36960\,C_2\,L^2\,b^5\,h^6\,r_0\,t^3\,\pi^3+36960\,C_2\,L^2\,b^5\,h^6\,{r_0}^3\,t\,\pi^3+$
$36960\,C_2\,L^2\,b^6\,h^5\,r_0\,t^3\,\pi^3+36960\,C_2\,L^2\,b^6\,h^5\,{r_0}^3\,t\,\pi^3+26400\,C_2\,L^2\,b^7\,h^4\,r_0\,t^3\,\pi^3+$
$26400\,C_2\,L^2\,b^7\,h^4\,{r_0}^3\,t\,\pi^3+13200\,C_2\,L^2\,b^8\,h^3\,r_0\,t^3\,\pi^3+13200\,C_2\,L^2\,b^8\,h^3\,{r_0}^3\,t\,\pi^3+$
$4400\,C_2\,L^2\,b^9\,h^2\,r_0\,t^3\,\pi^3+4400\,C_2\,L^2\,b^9\,h^2\,{r_0}^3\,t\,\pi^3+1320\,C_2\,L^2\,b^{10}\,h\,{r_0}^2\,t^2\,\pi^3+$
$1280664\,C_3\,L^2\,b^2\,h^{10}\,r_0\,t^2\,\pi^2+1280664\,C_3\,L^2\,b^2\,h^{10}\,{r_0}^2\,t\,\pi^2+4268880\,C_3\,L^2\,b^3\,h^9\,r_0\,t^2\,\pi^2+$
$4268880\,C_3\,L^2\,b^3\,h^9\,{r_0}^2\,t\,\pi^2+9604980\,C_3\,L^2\,b^4\,h^8\,r_0\,t^2\,\pi^2+9604980\,C_3\,L^2\,b^4\,h^8\,{r_0}^2\,t\,\pi^2+$
$15367968\,C_3\,L^2\,b^5\,h^7\,r_0\,t^2\,\pi^2+15367968\,C_3\,L^2\,b^5\,h^7\,{r_0}^2\,t\,\pi^2+17929296\,C_3\,L^2\,b^6\,h^6\,r_0\,t^2\,\pi^2+$
$17929296\,C_3\,L^2\,b^6\,h^6\,{r_0}^2\,t\,\pi^2+15367968\,C_3\,L^2\,b^7\,h^5\,r_0\,t^2\,\pi^2+15367968\,C_3\,L^2\,b^7\,h^5\,{r_0}^2\,t\,\pi^2+$

$9604980\,C_3\,L^2\,b^8\,h^4\,r_0\,t^2\,\pi^2+9604980\,C_3\,L^2\,b^8\,h^4\,{r_0}^2\,t\,\pi^2+4268880\,C_3\,L^2\,b^9\,h^3\,r_0\,t^2\,\pi^2+$
$4268880\,C_3\,L^2\,b^9\,h^3\,{r_0}^2\,t\,\pi^2+1280664\,C_3\,L^2\,b^{10}\,h^2\,r_0\,t^2\,\pi^2+1280664\,C_3\,L^2\,b^{10}\,h^2\,{r_0}^2\,t\,\pi^2+$
$280764\,C_3\,L^2\,b\,h^{10}\,{r_0}^2\,t^2\,\pi^3+935880\,C_3\,L^2\,b^2\,h^9\,r_0\,t^3\,\pi^3+935880\,C_3\,L^2\,b^2\,h^9\,{r_0}^3\,t\,\pi^3+$
$2807640\,C_3\,L^2\,b^3\,h^8\,r_0\,t^3\,\pi^3+2807640\,C_3\,L^2\,b^3\,h^8\,{r_0}^3\,t\,\pi^3+5615280\,C_3\,L^2\,b^4\,h^7\,r_0\,t^3\,\pi^3+$
$5615280\,C_3\,L^2\,b^4\,h^7\,{r_0}^3\,t\,\pi^3+7861392\,C_3\,L^2\,b^5\,h^6\,r_0\,t^3\,\pi^3+7861392\,C_3\,L^2\,b^5\,h^6\,{r_0}^3\,t\,\pi^3+$
$7861392\,C_3\,L^2\,b^6\,h^5\,r_0\,t^3\,\pi^3+7861392\,C_3\,L^2\,b^6\,h^5\,{r_0}^3\,t\,\pi^3+5615280\,C_3\,L^2\,b^7\,h^4\,r_0\,t^3\,\pi^3+$
$5615280\,C_3\,L^2\,b^7\,h^4\,{r_0}^3\,t\,\pi^3+2807640\,C_3\,L^2\,b^8\,h^3\,r_0\,t^3\,\pi^3+2807640\,C_3\,L^2\,b^8\,h^3\,{r_0}^3\,t\,\pi^3+$
$935880\,C_3\,L^2\,b^9\,h^2\,r_0\,t^3\,\pi^3+935880\,C_3\,L^2\,b^9\,h^2\,{r_0}^3\,t\,\pi^3+280764\,C_3\,L^2\,b^{10}\,h\,{r_0}^2\,t^2\,\pi^3$

$\mathrm{a}_{t,11}=576\,\pi\,C_2\,L^3\,b^{12}\,t^2+22752\,\pi\,C_3\,L^3\,b^{12}\,t^2+576\,\pi\,C_2\,L^3\,h^{12}\,t^2+22752\,\pi\,C_3\,L^3\,h^{12}\,t^2+$
$448\,C_2\,L^3\,b^{11}\,t^3\,\pi^2+208\,C_2\,L^3\,b^{10}\,t^4\,\pi^3+22272\,C_3\,L^3\,b^{11}\,t^3\,\pi^2+13800\,C_3\,L^3\,b^{10}\,t^4\,\pi^3+$
$448\,C_2\,L^3\,h^{11}\,t^3\,\pi^2+208\,C_2\,L^3\,h^{10}\,t^4\,\pi^3+22272\,C_3\,L^3\,h^{11}\,t^3\,\pi^2+13800\,C_3\,L^3\,h^{10}\,t^4\,\pi^3+$
$1344\,C_2\,L^3\,b^{11}\,r_0\,t^2\,\pi^2+1344\,C_2\,L^3\,b^{11}\,{r_0}^2\,t\,\pi^2+832\,C_2\,L^3\,b^{10}\,r_0\,t^3\,\pi^3+832\,C_2\,L^3\,b^{10}\,{r_0}^3\,t\,\pi^3+$
$66816\,C_3\,L^3\,b^{11}\,r_0\,t^2\,\pi^2+66816\,C_3\,L^3\,b^{11}\,{r_0}^2\,t\,\pi^2+55200\,C_3\,L^3\,b^{10}\,r_0\,t^3\,\pi^3+55200\,C_3\,L^3\,b^{10}\,{r_0}^3\,t\,\pi^3+$
$1344\,C_2\,L^3\,h^{11}\,r_0\,t^2\,\pi^2+1344\,C_2\,L^3\,h^{11}\,{r_0}^2\,t\,\pi^2+832\,C_2\,L^3\,h^{10}\,r_0\,t^3\,\pi^3+832\,C_2\,L^3\,h^{10}\,{r_0}^3\,t\,\pi^3+$
$66816\,C_3\,L^3\,h^{11}\,r_0\,t^2\,\pi^2+66816\,C_3\,L^3\,h^{11}\,{r_0}^2\,t\,\pi^2+55200\,C_3\,L^3\,h^{10}\,r_0\,t^3\,\pi^3+55200\,C_3\,L^3\,h^{10}\,{r_0}^3\,t\,\pi^3+$
$1152\,\pi\,C_2\,L^3\,b^{12}\,r_0\,t+45504\,\pi\,C_3\,L^3\,b^{12}\,r_0\,t+1152\,\pi\,C_2\,L^3\,h^{12}\,r_0\,t+45504\,\pi\,C_3\,L^3\,h^{12}\,r_0\,t+$
$24640\,C_2\,L^3\,b^2\,h^9\,t^3\,\pi^2+73920\,C_2\,L^3\,b^3\,h^8\,t^3\,\pi^2+147840\,C_2\,L^3\,b^4\,h^7\,t^3\,\pi^2+206976\,C_2\,L^3\,b^5\,h^6\,t^3\,\pi^2+$
$206976\,C_2\,L^3\,b^6\,h^5\,t^3\,\pi^2+147840\,C_2\,L^3\,b^7\,h^4\,t^3\,\pi^2+73920\,C_2\,L^3\,b^8\,h^3\,t^3\,\pi^2+24640\,C_2\,L^3\,b^9\,h^2\,t^3\,\pi^2+$
$9360\,C_2\,L^3\,b^2\,h^8\,t^4\,\pi^3+24960\,C_2\,L^3\,b^3\,h^7\,t^4\,\pi^3+43680\,C_2\,L^3\,b^4\,h^6\,t^4\,\pi^3+52416\,C_2\,L^3\,b^5\,h^5\,t^4\,\pi^3+$
$43680\,C_2\,L^3\,b^6\,h^4\,t^4\,\pi^3+24960\,C_2\,L^3\,b^7\,h^3\,t^4\,\pi^3+9360\,C_2\,L^3\,b^8\,h^2\,t^4\,\pi^3+1224960\,C_3\,L^3\,b^2\,h^9\,t^3\,\pi^2+$
$3674880\,C_3\,L^3\,b^3\,h^8\,t^3\,\pi^2+7349760\,C_3\,L^3\,b^4\,h^7\,t^3\,\pi^2+10289664\,C_3\,L^3\,b^5\,h^6\,t^3\,\pi^2+$
$10289664\,C_3\,L^3\,b^6\,h^5\,t^3\,\pi^2+7349760\,C_3\,L^3\,b^7\,h^4\,t^3\,\pi^2+3674880\,C_3\,L^3\,b^8\,h^3\,t^3\,\pi^2+$
$1224960\,C_3\,L^3\,b^9\,h^2\,t^3\,\pi^2+621000\,C_3\,L^3\,b^2\,h^8\,t^4\,\pi^3+1656000\,C_3\,L^3\,b^3\,h^7\,t^4\,\pi^3+$
$2898000\,C_3\,L^3\,b^4\,h^6\,t^4\,\pi^3+3477600\,C_3\,L^3\,b^5\,h^5\,t^4\,\pi^3+2898000\,C_3\,L^3\,b^6\,h^4\,t^4\,\pi^3+$
$1656000\,C_3\,L^3\,b^7\,h^3\,t^4\,\pi^3+621000\,C_3\,L^3\,b^8\,h^2\,t^4\,\pi^3+1248\,C_2\,L^3\,b^{10}\,{r_0}^2\,t^2\,\pi^3+$
$82800\,C_3\,L^3\,b^{10}\,{r_0}^2\,t^2\,\pi^3+1248\,C_2\,L^3\,h^{10}\,{r_0}^2\,t^2\,\pi^3+82800\,C_3\,L^3\,h^{10}\,{r_0}^2\,t^2\,\pi^3+$
$6912\,\pi\,C_2\,L^3\,b\,h^{11}\,t^2+6912\,\pi\,C_2\,L^3\,b^{11}\,h\,t^2+273024\,\pi\,C_3\,L^3\,b\,h^{11}\,t^2+273024\,\pi\,C_3\,L^3\,b^{11}\,h\,t^2+$
$38016\,\pi\,C_2\,L^3\,b^2\,h^{10}\,t^2+126720\,\pi\,C_2\,L^3\,b^3\,h^9\,t^2+285120\,\pi\,C_2\,L^3\,b^4\,h^8\,t^2+456192\,\pi\,C_2\,L^3\,b^5\,h^7\,t^2+$
$532224\,\pi\,C_2\,L^3\,b^6\,h^6\,t^2+456192\,\pi\,C_2\,L^3\,b^7\,h^5\,t^2+285120\,\pi\,C_2\,L^3\,b^8\,h^4\,t^2+126720\,\pi\,C_2\,L^3\,b^9\,h^3\,t^2+$
$38016\,\pi\,C_2\,L^3\,b^{10}\,h^2\,t^2+4928\,C_2\,L^3\,b\,h^{10}\,t^3\,\pi^2+4928\,C_2\,L^3\,b^{10}\,h\,t^3\,\pi^2+1501632\,\pi\,C_3\,L^3\,b^2\,h^{10}\,t^2+$
$5005440\,\pi\,C_3\,L^3\,b^3\,h^9\,t^2+11262240\,\pi\,C_3\,L^3\,b^4\,h^8\,t^2+18019584\,\pi\,C_3\,L^3\,b^5\,h^7\,t^2+$
$21022848\,\pi\,C_3\,L^3\,b^6\,h^6\,t^2+18019584\,\pi\,C_3\,L^3\,b^7\,h^5\,t^2+11262240\,\pi\,C_3\,L^3\,b^8\,h^4\,t^2+$
$5005440\,\pi\,C_3\,L^3\,b^9\,h^3\,t^2+1501632\,\pi\,C_3\,L^3\,b^{10}\,h^2\,t^2+2080\,C_2\,L^3\,b\,h^9\,t^4\,\pi^3+2080\,C_2\,L^3\,b^9\,h\,t^4\,\pi^3+$
$244992\,C_3\,L^3\,b\,h^{10}\,t^3\,\pi^2+244992\,C_3\,L^3\,b^{10}\,h\,t^3\,\pi^2+138000\,C_3\,L^3\,b\,h^9\,t^4\,\pi^3+138000\,C_3\,L^3\,b^9\,h\,t^4\,\pi^3+$
$76032\,\pi\,C_2\,L^3\,b^2\,h^{10}\,r_0\,t+253440\,\pi\,C_2\,L^3\,b^3\,h^9\,r_0\,t+570240\,\pi\,C_2\,L^3\,b^4\,h^8\,r_0\,t+$
$912384\,\pi\,C_2\,L^3\,b^5\,h^7\,r_0\,t+1064448\,\pi\,C_2\,L^3\,b^6\,h^6\,r_0\,t+912384\,\pi\,C_2\,L^3\,b^7\,h^5\,r_0\,t+$
$570240\,\pi\,C_2\,L^3\,b^8\,h^4\,r_0\,t+253440\,\pi\,C_2\,L^3\,b^9\,h^3\,r_0\,t+76032\,\pi\,C_2\,L^3\,b^{10}\,h^2\,r_0\,t+$
$3003264\,\pi\,C_3\,L^3\,b^2\,h^{10}\,r_0\,t+10010880\,\pi\,C_3\,L^3\,b^3\,h^9\,r_0\,t+22524480\,\pi\,C_3\,L^3\,b^4\,h^8\,r_0\,t+$
$36039168\,\pi\,C_3\,L^3\,b^5\,h^7\,r_0\,t+42045696\,\pi\,C_3\,L^3\,b^6\,h^6\,r_0\,t+36039168\,\pi\,C_3\,L^3\,b^7\,h^5\,r_0\,t+$
$22524480\,\pi\,C_3\,L^3\,b^8\,h^4\,r_0\,t+10010880\,\pi\,C_3\,L^3\,b^9\,h^3\,r_0\,t+3003264\,\pi\,C_3\,L^3\,b^{10}\,h^2\,r_0\,t+$
$56160\,C_2\,L^3\,b^2\,h^8\,{r_0}^2\,t^2\,\pi^3+149760\,C_2\,L^3\,b^3\,h^7\,{r_0}^2\,t^2\,\pi^3+262080\,C_2\,L^3\,b^4\,h^6\,{r_0}^2\,t^2\,\pi^3+$
$314496\,C_2\,L^3\,b^5\,h^5\,{r_0}^2\,t^2\,\pi^3+262080\,C_2\,L^3\,b^6\,h^4\,{r_0}^2\,t^2\,\pi^3+149760\,C_2\,L^3\,b^7\,h^3\,{r_0}^2\,t^2\,\pi^3+$
$56160\,C_2\,L^3\,b^8\,h^2\,{r_0}^2\,t^2\,\pi^3+3726000\,C_3\,L^3\,b^2\,h^8\,{r_0}^2\,t^2\,\pi^3+9936000\,C_3\,L^3\,b^3\,h^7\,{r_0}^2\,t^2\,\pi^3+$
$17388000\,C_3\,L^3\,b^4\,h^6\,{r_0}^2\,t^2\,\pi^3+20865600\,C_3\,L^3\,b^5\,h^5\,{r_0}^2\,t^2\,\pi^3+17388000\,C_3\,L^3\,b^6\,h^4\,{r_0}^2\,t^2\,\pi^3+$
$9936000\,C_3\,L^3\,b^7\,h^3\,{r_0}^2\,t^2\,\pi^3+3726000\,C_3\,L^3\,b^8\,h^2\,{r_0}^2\,t^2\,\pi^3+14784\,C_2\,L^3\,b\,h^{10}\,r_0\,t^2\,\pi^2+$

$14784\, C_2\, L^3\, b\, h^{10}\, {r_0}^2\, t\, \pi^2 + 14784\, C_2\, L^3\, b^{10}\, h\, r_0\, t^2\, \pi^2 + 14784\, C_2\, L^3\, b^{10}\, h\, {r_0}^2\, t\, \pi^2 +$
$8320\, C_2\, L^3\, b\, h^9\, r_0\, t^3\, \pi^3 + 8320\, C_2\, L^3\, b\, h^9\, {r_0}^3\, t\, \pi^3 + 8320\, C_2\, L^3\, b^9\, h\, r_0\, t^3\, \pi^3 + 8320\, C_2\, L^3\, b^9\, h\, {r_0}^3\, t\, \pi^3 +$
$734976\, C_3\, L^3\, b\, h^{10}\, r_0\, t^2\, \pi^2 + 734976\, C_3\, L^3\, b\, h^{10}\, {r_0}^2\, t\, \pi^2 + 734976\, C_3\, L^3\, b^{10}\, h\, r_0\, t^2\, \pi^2 +$
$734976\, C_3\, L^3\, b^{10}\, h\, {r_0}^2\, t\, \pi^2 + 552000\, C_3\, L^3\, b\, h^9\, r_0\, t^3\, \pi^3 + 552000\, C_3\, L^3\, b\, h^9\, {r_0}^3\, t\, \pi^3 +$
$552000\, C_3\, L^3\, b^9\, h\, r_0\, t^3\, \pi^3 + 552000\, C_3\, L^3\, b^9\, h\, {r_0}^3\, t\, \pi^3 + 13824\, \pi\, C_2\, L^3\, b\, h^{11}\, r_0\, t +$
$13824\, \pi\, C_2\, L^3\, b^{11}\, h\, r_0\, t + 546048\, \pi\, C_3\, L^3\, b\, h^{11}\, r_0\, t + 546048\, \pi\, C_3\, L^3\, b^{11}\, h\, r_0\, t + 73920\, C_2\, L^3\, b^2\, h^9\, r_0\, t^2\, \pi^2 +$
$73920\, C_2\, L^3\, b^2\, h^9\, {r_0}^2\, t\, \pi^2 + 221760\, C_2\, L^3\, b^3\, h^8\, r_0\, t^2\, \pi^2 + 221760\, C_2\, L^3\, b^3\, h^8\, {r_0}^2\, t\, \pi^2 +$
$443520\, C_2\, L^3\, b^4\, h^7\, r_0\, t^2\, \pi^2 + 443520\, C_2\, L^3\, b^4\, h^7\, {r_0}^2\, t\, \pi^2 + 620928\, C_2\, L^3\, b^5\, h^6\, r_0\, t^2\, \pi^2 +$
$620928\, C_2\, L^3\, b^5\, h^6\, {r_0}^2\, t\, \pi^2 + 620928\, C_2\, L^3\, b^6\, h^5\, r_0\, t^2\, \pi^2 + 620928\, C_2\, L^3\, b^6\, h^5\, {r_0}^2\, t\, \pi^2 +$
$443520\, C_2\, L^3\, b^7\, h^4\, r_0\, t^2\, \pi^2 + 443520\, C_2\, L^3\, b^7\, h^4\, {r_0}^2\, t\, \pi^2 + 221760\, C_2\, L^3\, b^8\, h^3\, r_0\, t^2\, \pi^2 +$
$221760\, C_2\, L^3\, b^8\, h^3\, {r_0}^2\, t\, \pi^2 + 73920\, C_2\, L^3\, b^9\, h^2\, r_0\, t^2\, \pi^2 + 73920\, C_2\, L^3\, b^9\, h^2\, {r_0}^2\, t\, \pi^2 +$
$12480\, C_2\, L^3\, b\, h^9\, {r_0}^2\, t^2\, \pi^3 + 37440\, C_2\, L^3\, b^2\, h^8\, r_0\, t^3\, \pi^3 + 37440\, C_2\, L^3\, b^2\, h^8\, {r_0}^3\, t\, \pi^3 +$
$99840\, C_2\, L^3\, b^3\, h^7\, r_0\, t^3\, \pi^3 + 99840\, C_2\, L^3\, b^3\, h^7\, {r_0}^3\, t\, \pi^3 + 174720\, C_2\, L^3\, b^4\, h^6\, r_0\, t^3\, \pi^3 +$
$174720\, C_2\, L^3\, b^4\, h^6\, {r_0}^3\, t\, \pi^3 + 209664\, C_2\, L^3\, b^5\, h^5\, r_0\, t^3\, \pi^3 + 209664\, C_2\, L^3\, b^5\, h^5\, {r_0}^3\, t\, \pi^3 +$
$174720\, C_2\, L^3\, b^6\, h^4\, r_0\, t^3\, \pi^3 + 174720\, C_2\, L^3\, b^6\, h^4\, {r_0}^3\, t\, \pi^3 + 99840\, C_2\, L^3\, b^7\, h^3\, r_0\, t^3\, \pi^3 +$
$99840\, C_2\, L^3\, b^7\, h^3\, {r_0}^3\, t\, \pi^3 + 37440\, C_2\, L^3\, b^8\, h^2\, r_0\, t^3\, \pi^3 + 37440\, C_2\, L^3\, b^8\, h^2\, {r_0}^3\, t\, \pi^3 +$
$12480\, C_2\, L^3\, b^9\, h\, {r_0}^2\, t^2\, \pi^3 + 3674880\, C_3\, L^3\, b^2\, h^9\, r_0\, t^2\, \pi^2 + 3674880\, C_3\, L^3\, b^2\, h^9\, {r_0}^2\, t\, \pi^2 +$
$11024640\, C_3\, L^3\, b^3\, h^8\, r_0\, t^2\, \pi^2 + 11024640\, C_3\, L^3\, b^3\, h^8\, {r_0}^2\, t\, \pi^2 + 22049280\, C_3\, L^3\, b^4\, h^7\, r_0\, t^2\, \pi^2 +$
$22049280\, C_3\, L^3\, b^4\, h^7\, {r_0}^2\, t\, \pi^2 + 30868992\, C_3\, L^3\, b^5\, h^6\, r_0\, t^2\, \pi^2 + 30868992\, C_3\, L^3\, b^5\, h^6\, {r_0}^2\, t\, \pi^2 +$
$30868992\, C_3\, L^3\, b^6\, h^5\, r_0\, t^2\, \pi^2 + 30868992\, C_3\, L^3\, b^6\, h^5\, {r_0}^2\, t\, \pi^2 + 22049280\, C_3\, L^3\, b^7\, h^4\, r_0\, t^2\, \pi^2 +$
$22049280\, C_3\, L^3\, b^7\, h^4\, {r_0}^2\, t\, \pi^2 + 11024640\, C_3\, L^3\, b^8\, h^3\, r_0\, t^2\, \pi^2 + 11024640\, C_3\, L^3\, b^8\, h^3\, {r_0}^2\, t\, \pi^2 +$
$3674880\, C_3\, L^3\, b^9\, h^2\, r_0\, t^2\, \pi^2 + 3674880\, C_3\, L^3\, b^9\, h^2\, {r_0}^2\, t\, \pi^2 + 828000\, C_3\, L^3\, b\, h^9\, {r_0}^2\, t^2\, \pi^3 +$
$2484000\, C_3\, L^3\, b^2\, h^8\, r_0\, t^3\, \pi^3 + 2484000\, C_3\, L^3\, b^2\, h^8\, {r_0}^3\, t\, \pi^3 + 6624000\, C_3\, L^3\, b^3\, h^7\, r_0\, t^3\, \pi^3 +$
$6624000\, C_3\, L^3\, b^3\, h^7\, {r_0}^3\, t\, \pi^3 + 11592000\, C_3\, L^3\, b^4\, h^6\, r_0\, t^3\, \pi^3 + 11592000\, C_3\, L^3\, b^4\, h^6\, {r_0}^3\, t\, \pi^3 +$
$13910400\, C_3\, L^3\, b^5\, h^5\, r_0\, t^3\, \pi^3 + 13910400\, C_3\, L^3\, b^5\, h^5\, {r_0}^3\, t\, \pi^3 + 11592000\, C_3\, L^3\, b^6\, h^4\, r_0\, t^3\, \pi^3 +$
$11592000\, C_3\, L^3\, b^6\, h^4\, {r_0}^3\, t\, \pi^3 + 6624000\, C_3\, L^3\, b^7\, h^3\, r_0\, t^3\, \pi^3 + 6624000\, C_3\, L^3\, b^7\, h^3\, {r_0}^3\, t\, \pi^3 +$
$2484000\, C_3\, L^3\, b^8\, h^2\, r_0\, t^3\, \pi^3 + 2484000\, C_3\, L^3\, b^8\, h^2\, {r_0}^3\, t\, \pi^3 + 828000\, C_3\, L^3\, b^9\, h\, {r_0}^2\, t^2\, \pi^3$

$\mathrm{a}_{t,10} = 24\, \pi\, C_1\, L^4\, b^{11}\, t^2 + 3072\, \pi\, C_2\, L^4\, b^{11}\, t^2 + 53424\, \pi\, C_3\, L^4\, b^{11}\, t^2 + 24\, \pi\, C_1\, L^4\, h^{11}\, t^2 +$
$3072\, \pi\, C_2\, L^4\, h^{11}\, t^2 + 53424\, \pi\, C_3\, L^4\, h^{11}\, t^2 + 12\, C_1\, L^4\, b^{10}\, t^3\, \pi^2 + 3\, C_1\, L^4\, b^9\, t^4\, \pi^3 +$
$2240\, C_2\, L^4\, b^{10}\, t^3\, \pi^2 + 968\, C_2\, L^4\, b^9\, t^4\, \pi^3 + 49944\, C_3\, L^4\, b^{10}\, t^3\, \pi^2 + 29310\, C_3\, L^4\, b^9\, t^4\, \pi^3 +$
$12\, C_1\, L^4\, h^{10}\, t^3\, \pi^2 + 3\, C_1\, L^4\, h^9\, t^4\, \pi^3 + 2240\, C_2\, L^4\, h^{10}\, t^3\, \pi^2 + 968\, C_2\, L^4\, h^9\, t^4\, \pi^3 +$
$49944\, C_3\, L^4\, h^{10}\, t^3\, \pi^2 + 29310\, C_3\, L^4\, h^9\, t^4\, \pi^3 + 36\, C_1\, L^4\, b^{10}\, r_0\, t^2\, \pi^2 + 36\, C_1\, L^4\, b^{10}\, {r_0}^2\, t\, \pi^2 +$
$12\, C_1\, L^4\, b^9\, r_0\, t^3\, \pi^3 + 12\, C_1\, L^4\, b^9\, {r_0}^3\, t\, \pi^3 + 6720\, C_2\, L^4\, b^{10}\, r_0\, t^2\, \pi^2 + 6720\, C_2\, L^4\, b^{10}\, {r_0}^2\, t\, \pi^2 +$
$3872\, C_2\, L^4\, b^9\, r_0\, t^3\, \pi^3 + 3872\, C_2\, L^4\, b^9\, {r_0}^3\, t\, \pi^3 + 149832\, C_3\, L^4\, b^{10}\, r_0\, t^2\, \pi^2 + 149832\, C_3\, L^4\, b^{10}\, {r_0}^2\, t\, \pi^2 +$
$117240\, C_3\, L^4\, b^9\, r_0\, t^3\, \pi^3 + 117240\, C_3\, L^4\, b^9\, {r_0}^3\, t\, \pi^3 + 36\, C_1\, L^4\, h^{10}\, r_0\, t^2\, \pi^2 + 36\, C_1\, L^4\, h^{10}\, {r_0}^2\, t\, \pi^2 +$
$12\, C_1\, L^4\, h^9\, r_0\, t^3\, \pi^3 + 12\, C_1\, L^4\, h^9\, {r_0}^3\, t\, \pi^3 + 6720\, C_2\, L^4\, h^{10}\, r_0\, t^2\, \pi^2 + 6720\, C_2\, L^4\, h^{10}\, {r_0}^2\, t\, \pi^2 +$
$3872\, C_2\, L^4\, h^9\, r_0\, t^3\, \pi^3 + 3872\, C_2\, L^4\, h^9\, {r_0}^3\, t\, \pi^3 + 149832\, C_3\, L^4\, h^{10}\, r_0\, t^2\, \pi^2 + 149832\, C_3\, L^4\, h^{10}\, {r_0}^2\, t\, \pi^2 +$
$117240\, C_3\, L^4\, h^9\, r_0\, t^3\, \pi^3 + 117240\, C_3\, L^4\, h^9\, {r_0}^3\, t\, \pi^3 + 48\, \pi\, C_1\, L^4\, b^{11}\, r_0\, t + 6144\, \pi\, C_2\, L^4\, b^{11}\, r_0\, t +$
$106848\, \pi\, C_3\, L^4\, b^{11}\, r_0\, t + 48\, \pi\, C_1\, L^4\, h^{11}\, r_0\, t + 6144\, \pi\, C_2\, L^4\, h^{11}\, r_0\, t + 106848\, \pi\, C_3\, L^4\, h^{11}\, r_0\, t +$
$540\, C_1\, L^4\, b^2\, h^8\, t^3\, \pi^2 + 1440\, C_1\, L^4\, b^3\, h^7\, t^3\, \pi^2 + 2520\, C_1\, L^4\, b^4\, h^6\, t^3\, \pi^2 + 3024\, C_1\, L^4\, b^5\, h^5\, t^3\, \pi^2 +$
$2520\, C_1\, L^4\, b^6\, h^4\, t^3\, \pi^2 + 1440\, C_1\, L^4\, b^7\, h^3\, t^3\, \pi^2 + 540\, C_1\, L^4\, b^8\, h^2\, t^3\, \pi^2 + 108\, C_1\, L^4\, b^2\, h^7\, t^4\, \pi^3 +$
$252\, C_1\, L^4\, b^3\, h^6\, t^4\, \pi^3 + 378\, C_1\, L^4\, b^4\, h^5\, t^4\, \pi^3 + 378\, C_1\, L^4\, b^5\, h^4\, t^4\, \pi^3 + 252\, C_1\, L^4\, b^6\, h^3\, t^4\, \pi^3 +$
$108\, C_1\, L^4\, b^7\, h^2\, t^4\, \pi^3 + 100800\, C_2\, L^4\, b^2\, h^8\, t^3\, \pi^2 + 268800\, C_2\, L^4\, b^3\, h^7\, t^3\, \pi^2 + 470400\, C_2\, L^4\, b^4\, h^6\, t^3\, \pi^2 +$
$564480\, C_2\, L^4\, b^5\, h^5\, t^3\, \pi^2 + 470400\, C_2\, L^4\, b^6\, h^4\, t^3\, \pi^2 + 268800\, C_2\, L^4\, b^7\, h^3\, t^3\, \pi^2 + 100800\, C_2\, L^4\, b^8\, h^2\, t^3\, \pi^2 +$
$34848\, C_2\, L^4\, b^2\, h^7\, t^4\, \pi^3 + 81312\, C_2\, L^4\, b^3\, h^6\, t^4\, \pi^3 + 121968\, C_2\, L^4\, b^4\, h^5\, t^4\, \pi^3 + 121968\, C_2\, L^4\, b^5\, h^4\, t^4\, \pi^3 +$

$81312\,C_2\,L^4\,b^6\,h^3\,t^4\,\pi^3+34848\,C_2\,L^4\,b^7\,h^2\,t^4\,\pi^3+2247480\,C_3\,L^4\,b^2\,h^8\,t^3\,\pi^2+5993280\,C_3\,L^4\,b^3\,h^7\,t^3\,\pi^2+$
$10488240\,C_3\,L^4\,b^4\,h^6\,t^3\,\pi^2+12585888\,C_3\,L^4\,b^5\,h^5\,t^3\,\pi^2+10488240\,C_3\,L^4\,b^6\,h^4\,t^3\,\pi^2+$
$5993280\,C_3\,L^4\,b^7\,h^3\,t^3\,\pi^2+2247480\,C_3\,L^4\,b^8\,h^2\,t^3\,\pi^2+1055160\,C_3\,L^4\,b^2\,h^7\,t^4\,\pi^3+$
$2462040\,C_3\,L^4\,b^3\,h^6\,t^4\,\pi^3+3693060\,C_3\,L^4\,b^4\,h^5\,t^4\,\pi^3+3693060\,C_3\,L^4\,b^5\,h^4\,t^4\,\pi^3+$
$2462040\,C_3\,L^4\,b^6\,h^3\,t^4\,\pi^3+1055160\,C_3\,L^4\,b^7\,h^2\,t^4\,\pi^3+18\,C_1\,L^4\,b^9\,{r_0}^2\,t^2\,\pi^3+5808\,C_2\,L^4\,b^9\,{r_0}^2\,t^2\,\pi^3+$
$175860\,C_3\,L^4\,b^9\,{r_0}^2\,t^2\,\pi^3+18\,C_1\,L^4\,h^9\,{r_0}^2\,t^2\,\pi^3+5808\,C_2\,L^4\,h^9\,{r_0}^2\,t^2\,\pi^3+175860\,C_3\,L^4\,h^9\,{r_0}^2\,t^2\,\pi^3+$
$264\,\pi\,C_1\,L^4\,b\,h^{10}\,t^2+264\,\pi\,C_1\,L^4\,b^{10}\,h\,t^2+33792\,\pi\,C_2\,L^4\,b\,h^{10}\,t^2+33792\,\pi\,C_2\,L^4\,b^{10}\,h\,t^2+$
$587664\,\pi\,C_3\,L^4\,b\,h^{10}\,t^2+587664\,\pi\,C_3\,L^4\,b^{10}\,h\,t^2+1320\,\pi\,C_1\,L^4\,b^2\,h^9\,t^2+3960\,\pi\,C_1\,L^4\,b^3\,h^8\,t^2+$
$7920\,\pi\,C_1\,L^4\,b^4\,h^7\,t^2+11088\,\pi\,C_1\,L^4\,b^5\,h^6\,t^2+11088\,\pi\,C_1\,L^4\,b^6\,h^5\,t^2+7920\,\pi\,C_1\,L^4\,b^7\,h^4\,t^2+$
$3960\,\pi\,C_1\,L^4\,b^8\,h^3\,t^2+1320\,\pi\,C_1\,L^4\,b^9\,h^2\,t^2+120\,C_1\,L^4\,b\,h^9\,t^3\,\pi^2+120\,C_1\,L^4\,b^9\,h\,t^3\,\pi^2+$
$168960\,\pi\,C_2\,L^4\,b^2\,h^9\,t^2+506880\,\pi\,C_2\,L^4\,b^3\,h^8\,t^2+1013760\,\pi\,C_2\,L^4\,b^4\,h^7\,t^2+1419264\,\pi\,C_2\,L^4\,b^5\,h^6\,t^2+$
$1419264\,\pi\,C_2\,L^4\,b^6\,h^5\,t^2+1013760\,\pi\,C_2\,L^4\,b^7\,h^4\,t^2+506880\,\pi\,C_2\,L^4\,b^8\,h^3\,t^2+168960\,\pi\,C_2\,L^4\,b^9\,h^2\,t^2+$
$27\,C_1\,L^4\,b\,h^8\,t^4\,\pi^3+27\,C_1\,L^4\,b^8\,h\,t^4\,\pi^3+22400\,C_2\,L^4\,b\,h^9\,t^3\,\pi^2+22400\,C_2\,L^4\,b^9\,h\,t^3\,\pi^2+$
$2938320\,\pi\,C_3\,L^4\,b^2\,h^9\,t^2+8814960\,\pi\,C_3\,L^4\,b^3\,h^8\,t^2+17629920\,\pi\,C_3\,L^4\,b^4\,h^7\,t^2+$
$24681888\,\pi\,C_3\,L^4\,b^5\,h^6\,t^2+24681888\,\pi\,C_3\,L^4\,b^6\,h^5\,t^2+17629920\,\pi\,C_3\,L^4\,b^7\,h^4\,t^2+$
$8814960\,\pi\,C_3\,L^4\,b^8\,h^3\,t^2+2938320\,\pi\,C_3\,L^4\,b^9\,h^2\,t^2+8712\,C_2\,L^4\,b\,h^8\,t^4\,\pi^3+8712\,C_2\,L^4\,b^8\,h\,t^4\,\pi^3+$
$499440\,C_3\,L^4\,b\,h^9\,t^3\,\pi^2+499440\,C_3\,L^4\,b^9\,h\,t^3\,\pi^2+263790\,C_3\,L^4\,b\,h^8\,t^4\,\pi^3+263790\,C_3\,L^4\,b^8\,h\,t^4\,\pi^3+$
$2640\,\pi\,C_1\,L^4\,b^2\,h^9\,r_0\,t+7920\,\pi\,C_1\,L^4\,b^3\,h^8\,r_0\,t+15840\,\pi\,C_1\,L^4\,b^4\,h^7\,r_0\,t+22176\,\pi\,C_1\,L^4\,b^5\,h^6\,r_0\,t+$
$22176\,\pi\,C_1\,L^4\,b^6\,h^5\,r_0\,t+15840\,\pi\,C_1\,L^4\,b^7\,h^4\,r_0\,t+7920\,\pi\,C_1\,L^4\,b^8\,h^3\,r_0\,t+2640\,\pi\,C_1\,L^4\,b^9\,h^2\,r_0\,t+$
$337920\,\pi\,C_2\,L^4\,b^2\,h^9\,r_0\,t+1013760\,\pi\,C_2\,L^4\,b^3\,h^8\,r_0\,t+2027520\,\pi\,C_2\,L^4\,b^4\,h^7\,r_0\,t+$
$2838528\,\pi\,C_2\,L^4\,b^5\,h^6\,r_0\,t+2838528\,\pi\,C_2\,L^4\,b^6\,h^5\,r_0\,t+2027520\,\pi\,C_2\,L^4\,b^7\,h^4\,r_0\,t+$
$1013760\,\pi\,C_2\,L^4\,b^8\,h^3\,r_0\,t+337920\,\pi\,C_2\,L^4\,b^9\,h^2\,r_0\,t+5876640\,\pi\,C_3\,L^4\,b^2\,h^9\,r_0\,t+$
$17629920\,\pi\,C_3\,L^4\,b^3\,h^8\,r_0\,t+35259840\,\pi\,C_3\,L^4\,b^4\,h^7\,r_0\,t+49363776\,\pi\,C_3\,L^4\,b^5\,h^6\,r_0\,t+$
$49363776\,\pi\,C_3\,L^4\,b^6\,h^5\,r_0\,t+35259840\,\pi\,C_3\,L^4\,b^7\,h^4\,r_0\,t+17629920\,\pi\,C_3\,L^4\,b^8\,h^3\,r_0\,t+$
$5876640\,\pi\,C_3\,L^4\,b^9\,h^2\,r_0\,t+648\,C_1\,L^4\,b^2\,h^7\,{r_0}^2\,t^2\,\pi^3+1512\,C_1\,L^4\,b^3\,h^6\,{r_0}^2\,t^2\,\pi^3+$
$2268\,C_1\,L^4\,b^4\,h^5\,{r_0}^2\,t^2\,\pi^3+2268\,C_1\,L^4\,b^5\,h^4\,{r_0}^2\,t^2\,\pi^3+1512\,C_1\,L^4\,b^6\,h^3\,{r_0}^2\,t^2\,\pi^3+$
$648\,C_1\,L^4\,b^7\,h^2\,{r_0}^2\,t^2\,\pi^3+209088\,C_2\,L^4\,b^2\,h^7\,{r_0}^2\,t^2\,\pi^3+487872\,C_2\,L^4\,b^3\,h^6\,{r_0}^2\,t^2\,\pi^3+$
$731808\,C_2\,L^4\,b^4\,h^5\,{r_0}^2\,t^2\,\pi^3+731808\,C_2\,L^4\,b^5\,h^4\,{r_0}^2\,t^2\,\pi^3+487872\,C_2\,L^4\,b^6\,h^3\,{r_0}^2\,t^2\,\pi^3+$
$209088\,C_2\,L^4\,b^7\,h^2\,{r_0}^2\,t^2\,\pi^3+6330960\,C_3\,L^4\,b^2\,h^7\,{r_0}^2\,t^2\,\pi^3+14772240\,C_3\,L^4\,b^3\,h^6\,{r_0}^2\,t^2\,\pi^3+$
$22158360\,C_3\,L^4\,b^4\,h^5\,{r_0}^2\,t^2\,\pi^3+22158360\,C_3\,L^4\,b^5\,h^4\,{r_0}^2\,t^2\,\pi^3+14772240\,C_3\,L^4\,b^6\,h^3\,{r_0}^2\,t^2\,\pi^3+$
$6330960\,C_3\,L^4\,b^7\,h^2\,{r_0}^2\,t^2\,\pi^3+360\,C_1\,L^4\,b\,h^9\,r_0\,t^2\,\pi^2+360\,C_1\,L^4\,b\,h^9\,{r_0}^2\,t\,\pi^2+$
$360\,C_1\,L^4\,b^9\,h\,r_0\,t^2\,\pi^2+360\,C_1\,L^4\,b^9\,h\,{r_0}^2\,t\,\pi^2+108\,C_1\,L^4\,b\,h^8\,r_0\,t^3\,\pi^3+108\,C_1\,L^4\,b\,h^8\,{r_0}^3\,t\,\pi^3+$
$108\,C_1\,L^4\,b^8\,h\,r_0\,t^3\,\pi^3+108\,C_1\,L^4\,b^8\,h\,{r_0}^3\,t\,\pi^3+67200\,C_2\,L^4\,b\,h^9\,r_0\,t^2\,\pi^2+67200\,C_2\,L^4\,b\,h^9\,{r_0}^2\,t\,\pi^2+$
$67200\,C_2\,L^4\,b^9\,h\,r_0\,t^2\,\pi^2+67200\,C_2\,L^4\,b^9\,h\,{r_0}^2\,t\,\pi^2+34848\,C_2\,L^4\,b\,h^8\,r_0\,t^3\,\pi^3+$
$34848\,C_2\,L^4\,b\,h^8\,{r_0}^3\,t\,\pi^3+34848\,C_2\,L^4\,b^8\,h\,r_0\,t^3\,\pi^3+34848\,C_2\,L^4\,b^8\,h\,{r_0}^3\,t\,\pi^3+$
$1498320\,C_3\,L^4\,b\,h^9\,r_0\,t^2\,\pi^2+1498320\,C_3\,L^4\,b\,h^9\,{r_0}^2\,t\,\pi^2+1498320\,C_3\,L^4\,b^9\,h\,r_0\,t^2\,\pi^2+$
$1498320\,C_3\,L^4\,b^9\,h\,{r_0}^2\,t\,\pi^2+1055160\,C_3\,L^4\,b\,h^8\,r_0\,t^3\,\pi^3+1055160\,C_3\,L^4\,b\,h^8\,{r_0}^3\,t\,\pi^3+$
$1055160\,C_3\,L^4\,b^8\,h\,r_0\,t^3\,\pi^3+1055160\,C_3\,L^4\,b^8\,h\,{r_0}^3\,t\,\pi^3+528\,\pi\,C_1\,L^4\,b\,h^{10}\,r_0\,t+$
$528\,\pi\,C_1\,L^4\,b^{10}\,h\,r_0\,t+67584\,\pi\,C_2\,L^4\,b\,h^{10}\,r_0\,t+67584\,\pi\,C_2\,L^4\,b^{10}\,h\,r_0\,t+1175328\,\pi\,C_3\,L^4\,b\,h^{10}\,r_0\,t+$
$1175328\,\pi\,C_3\,L^4\,b^{10}\,h\,r_0\,t+1620\,C_1\,L^4\,b^2\,h^8\,r_0\,t^2\,\pi^2+1620\,C_1\,L^4\,b^2\,h^8\,{r_0}^2\,t\,\pi^2+$
$4320\,C_1\,L^4\,b^3\,h^7\,r_0\,t^2\,\pi^2+4320\,C_1\,L^4\,b^3\,h^7\,{r_0}^2\,t\,\pi^2+7560\,C_1\,L^4\,b^4\,h^6\,r_0\,t^2\,\pi^2+$
$7560\,C_1\,L^4\,b^4\,h^6\,{r_0}^2\,t\,\pi^2+9072\,C_1\,L^4\,b^5\,h^5\,r_0\,t^2\,\pi^2+9072\,C_1\,L^4\,b^5\,h^5\,{r_0}^2\,t\,\pi^2+$
$7560\,C_1\,L^4\,b^6\,h^4\,r_0\,t^2\,\pi^2+7560\,C_1\,L^4\,b^6\,h^4\,{r_0}^2\,t\,\pi^2+4320\,C_1\,L^4\,b^7\,h^3\,r_0\,t^2\,\pi^2+$
$4320\,C_1\,L^4\,b^7\,h^3\,{r_0}^2\,t\,\pi^2+1620\,C_1\,L^4\,b^8\,h^2\,r_0\,t^2\,\pi^2+1620\,C_1\,L^4\,b^8\,h^2\,{r_0}^2\,t\,\pi^2+$
$162\,C_1\,L^4\,b\,h^8\,{r_0}^2\,t^2\,\pi^3+432\,C_1\,L^4\,b^2\,h^7\,r_0\,t^3\,\pi^3+432\,C_1\,L^4\,b^2\,h^7\,{r_0}^3\,t\,\pi^3+1008\,C_1\,L^4\,b^3\,h^6\,r_0\,t^3\,\pi^3+$
$1008\,C_1\,L^4\,b^3\,h^6\,{r_0}^3\,t\,\pi^3+1512\,C_1\,L^4\,b^4\,h^5\,r_0\,t^3\,\pi^3+1512\,C_1\,L^4\,b^4\,h^5\,{r_0}^3\,t\,\pi^3+$

$$1512\,C_1\,L^4\,b^5\,h^4\,r_0\,t^3\,\pi^3\;+\;1512\,C_1\,L^4\,b^5\,h^4\,{r_0}^3\,t\,\pi^3\;+\;1008\,C_1\,L^4\,b^6\,h^3\,r_0\,t^3\,\pi^3\;+$$
$$1008\,C_1\,L^4\,b^6\,h^3\,{r_0}^3\,t\,\pi^3+432\,C_1\,L^4\,b^7\,h^2\,r_0\,t^3\,\pi^3+432\,C_1\,L^4\,b^7\,h^2\,{r_0}^3\,t\,\pi^3+162\,C_1\,L^4\,b^8\,h\,{r_0}^2\,t^2\,\pi^3+$$
$$302400\,C_2\,L^4\,b^2\,h^8\,r_0\,t^2\,\pi^2+302400\,C_2\,L^4\,b^2\,h^8\,{r_0}^2\,t\,\pi^2+806400\,C_2\,L^4\,b^3\,h^7\,r_0\,t^2\,\pi^2+$$
$$806400\,C_2\,L^4\,b^3\,h^7\,{r_0}^2\,t\,\pi^2+1411200\,C_2\,L^4\,b^4\,h^6\,r_0\,t^2\,\pi^2+1411200\,C_2\,L^4\,b^4\,h^6\,{r_0}^2\,t\,\pi^2+$$
$$1693440\,C_2\,L^4\,b^5\,h^5\,r_0\,t^2\,\pi^2+1693440\,C_2\,L^4\,b^5\,h^5\,{r_0}^2\,t\,\pi^2+1411200\,C_2\,L^4\,b^6\,h^4\,r_0\,t^2\,\pi^2+$$
$$1411200\,C_2\,L^4\,b^6\,h^4\,{r_0}^2\,t\,\pi^2+806400\,C_2\,L^4\,b^7\,h^3\,r_0\,t^2\,\pi^2+806400\,C_2\,L^4\,b^7\,h^3\,{r_0}^2\,t\,\pi^2+$$
$$302400\,C_2\,L^4\,b^8\,h^2\,r_0\,t^2\,\pi^2+302400\,C_2\,L^4\,b^8\,h^2\,{r_0}^2\,t\,\pi^2+52272\,C_2\,L^4\,b\,h^8\,{r_0}^2\,t^2\,\pi^3+$$
$$139392\,C_2\,L^4\,b^2\,h^7\,r_0\,t^3\,\pi^3+139392\,C_2\,L^4\,b^2\,h^7\,{r_0}^3\,t\,\pi^3+325248\,C_2\,L^4\,b^3\,h^6\,r_0\,t^3\,\pi^3+$$
$$325248\,C_2\,L^4\,b^3\,h^6\,{r_0}^3\,t\,\pi^3+487872\,C_2\,L^4\,b^4\,h^5\,r_0\,t^3\,\pi^3+487872\,C_2\,L^4\,b^4\,h^5\,{r_0}^3\,t\,\pi^3+$$
$$487872\,C_2\,L^4\,b^5\,h^4\,r_0\,t^3\,\pi^3+487872\,C_2\,L^4\,b^5\,h^4\,{r_0}^3\,t\,\pi^3+325248\,C_2\,L^4\,b^6\,h^3\,r_0\,t^3\,\pi^3+$$
$$325248\,C_2\,L^4\,b^6\,h^3\,{r_0}^3\,t\,\pi^3+139392\,C_2\,L^4\,b^7\,h^2\,r_0\,t^3\,\pi^3+139392\,C_2\,L^4\,b^7\,h^2\,{r_0}^3\,t\,\pi^3+$$
$$52272\,C_2\,L^4\,b^8\,h\,{r_0}^2\,t^2\,\pi^3+6742440\,C_3\,L^4\,b^2\,h^8\,r_0\,t^2\,\pi^2+6742440\,C_3\,L^4\,b^2\,h^8\,{r_0}^2\,t\,\pi^2+$$
$$17979840\,C_3\,L^4\,b^3\,h^7\,r_0\,t^2\,\pi^2+17979840\,C_3\,L^4\,b^3\,h^7\,{r_0}^2\,t\,\pi^2+31464720\,C_3\,L^4\,b^4\,h^6\,r_0\,t^2\,\pi^2+$$
$$31464720\,C_3\,L^4\,b^4\,h^6\,{r_0}^2\,t\,\pi^2+37757664\,C_3\,L^4\,b^5\,h^5\,r_0\,t^2\,\pi^2+37757664\,C_3\,L^4\,b^5\,h^5\,{r_0}^2\,t\,\pi^2+$$
$$31464720\,C_3\,L^4\,b^6\,h^4\,r_0\,t^2\,\pi^2+31464720\,C_3\,L^4\,b^6\,h^4\,{r_0}^2\,t\,\pi^2+17979840\,C_3\,L^4\,b^7\,h^3\,r_0\,t^2\,\pi^2+$$
$$17979840\,C_3\,L^4\,b^7\,h^3\,{r_0}^2\,t\,\pi^2+6742440\,C_3\,L^4\,b^8\,h^2\,r_0\,t^2\,\pi^2+6742440\,C_3\,L^4\,b^8\,h^2\,{r_0}^2\,t\,\pi^2+$$
$$1582740\,C_3\,L^4\,b\,h^8\,{r_0}^2\,t^2\,\pi^3+4220640\,C_3\,L^4\,b^2\,h^7\,r_0\,t^3\,\pi^3+4220640\,C_3\,L^4\,b^2\,h^7\,{r_0}^3\,t\,\pi^3+$$
$$9848160\,C_3\,L^4\,b^3\,h^6\,r_0\,t^3\,\pi^3+9848160\,C_3\,L^4\,b^3\,h^6\,{r_0}^3\,t\,\pi^3+14772240\,C_3\,L^4\,b^4\,h^5\,r_0\,t^3\,\pi^3+$$
$$14772240\,C_3\,L^4\,b^4\,h^5\,{r_0}^3\,t\,\pi^3+14772240\,C_3\,L^4\,b^5\,h^4\,r_0\,t^3\,\pi^3+14772240\,C_3\,L^4\,b^5\,h^4\,{r_0}^3\,t\,\pi^3+$$
$$9848160\,C_3\,L^4\,b^6\,h^3\,r_0\,t^3\,\pi^3+9848160\,C_3\,L^4\,b^6\,h^3\,{r_0}^3\,t\,\pi^3+4220640\,C_3\,L^4\,b^7\,h^2\,r_0\,t^3\,\pi^3+$$
$$4220640\,C_3\,L^4\,b^7\,h^2\,{r_0}^3\,t\,\pi^3\;+\;1582740\,C_3\,L^4\,b^8\,h\,{r_0}^2\,t^2\,\pi^3$$

$$\mathrm{a}_{t,9} = 240\,\pi\,C_1\,L^5\,b^{10}\,t^2+9600\,\pi\,C_2\,L^5\,b^{10}\,t^2+84384\,\pi\,C_3\,L^5\,b^{10}\,t^2+240\,\pi\,C_1\,L^5\,h^{10}\,t^2+$$
$$9600\,\pi\,C_2\,L^5\,h^{10}\,t^2+84384\,\pi\,C_3\,L^5\,h^{10}\,t^2+112\,C_1\,L^5\,b^9\,t^3\,\pi^2+26\,C_1\,L^5\,b^8\,t^4\,\pi^3+$$
$$6592\,C_2\,L^5\,b^9\,t^3\,\pi^2+2656\,C_2\,L^5\,b^8\,t^4\,\pi^3+76128\,C_3\,L^5\,b^9\,t^3\,\pi^2+42636\,C_3\,L^5\,b^8\,t^4\,\pi^3+$$
$$112\,C_1\,L^5\,h^9\,t^3\,\pi^2+26\,C_1\,L^5\,h^8\,t^4\,\pi^3+6592\,C_2\,L^5\,h^9\,t^3\,\pi^2+2656\,C_2\,L^5\,h^8\,t^4\,\pi^3+$$
$$76128\,C_3\,L^5\,h^9\,t^3\,\pi^2+42636\,C_3\,L^5\,h^8\,t^4\,\pi^3+336\,C_1\,L^5\,b^9\,r_0\,t^2\,\pi^2+336\,C_1\,L^5\,b^9\,{r_0}^2\,t\,\pi^2+$$
$$104\,C_1\,L^5\,b^8\,r_0\,t^3\,\pi^3+104\,C_1\,L^5\,b^8\,{r_0}^3\,t\,\pi^3+19776\,C_2\,L^5\,b^9\,r_0\,t^2\,\pi^2+19776\,C_2\,L^5\,b^9\,{r_0}^2\,t\,\pi^2+$$
$$10624\,C_2\,L^5\,b^8\,r_0\,t^3\,\pi^3+10624\,C_2\,L^5\,b^8\,{r_0}^3\,t\,\pi^3+228384\,C_3\,L^5\,b^9\,r_0\,t^2\,\pi^2+228384\,C_3\,L^5\,b^9\,{r_0}^2\,t\,\pi^2+$$
$$170544\,C_3\,L^5\,b^8\,r_0\,t^3\,\pi^3+170544\,C_3\,L^5\,b^8\,{r_0}^3\,t\,\pi^3+336\,C_1\,L^5\,h^9\,r_0\,t^2\,\pi^2+336\,C_1\,L^5\,h^9\,{r_0}^2\,t\,\pi^2+$$
$$104\,C_1\,L^5\,h^8\,r_0\,t^3\,\pi^3+104\,C_1\,L^5\,h^8\,{r_0}^3\,t\,\pi^3+19776\,C_2\,L^5\,h^9\,r_0\,t^2\,\pi^2+19776\,C_2\,L^5\,h^9\,{r_0}^2\,t\,\pi^2+$$
$$10624\,C_2\,L^5\,h^8\,r_0\,t^3\,\pi^3+10624\,C_2\,L^5\,h^8\,{r_0}^3\,t\,\pi^3+228384\,C_3\,L^5\,h^9\,r_0\,t^2\,\pi^2+228384\,C_3\,L^5\,h^9\,{r_0}^2\,t\,\pi^2+$$
$$170544\,C_3\,L^5\,h^8\,r_0\,t^3\,\pi^3+170544\,C_3\,L^5\,h^8\,{r_0}^3\,t\,\pi^3+480\,\pi\,C_1\,L^5\,b^{10}\,r_0\,t+19200\,\pi\,C_2\,L^5\,b^{10}\,r_0\,t+$$
$$168768\,\pi\,C_3\,L^5\,b^{10}\,r_0\,t+480\,\pi\,C_1\,L^5\,h^{10}\,r_0\,t+19200\,\pi\,C_2\,L^5\,h^{10}\,r_0\,t+168768\,\pi\,C_3\,L^5\,h^{10}\,r_0\,t+$$
$$4032\,C_1\,L^5\,b^2\,h^7\,t^3\,\pi^2+9408\,C_1\,L^5\,b^3\,h^6\,t^3\,\pi^2+14112\,C_1\,L^5\,b^4\,h^5\,t^3\,\pi^2+14112\,C_1\,L^5\,b^5\,h^4\,t^3\,\pi^2+$$
$$9408\,C_1\,L^5\,b^6\,h^3\,t^3\,\pi^2+4032\,C_1\,L^5\,b^7\,h^2\,t^3\,\pi^2+728\,C_1\,L^5\,b^2\,h^6\,t^4\,\pi^3+1456\,C_1\,L^5\,b^3\,h^5\,t^4\,\pi^3+$$
$$1820\,C_1\,L^5\,b^4\,h^4\,t^4\,\pi^3+1456\,C_1\,L^5\,b^5\,h^3\,t^4\,\pi^3+728\,C_1\,L^5\,b^6\,h^2\,t^4\,\pi^3+237312\,C_2\,L^5\,b^2\,h^7\,t^3\,\pi^2+$$
$$553728\,C_2\,L^5\,b^3\,h^6\,t^3\,\pi^2+830592\,C_2\,L^5\,b^4\,h^5\,t^3\,\pi^2+830592\,C_2\,L^5\,b^5\,h^4\,t^3\,\pi^2+553728\,C_2\,L^5\,b^6\,h^3\,t^3\,\pi^2+$$
$$237312\,C_2\,L^5\,b^7\,h^2\,t^3\,\pi^2+74368\,C_2\,L^5\,b^2\,h^6\,t^4\,\pi^3+148736\,C_2\,L^5\,b^3\,h^5\,t^4\,\pi^3+185920\,C_2\,L^5\,b^4\,h^4\,t^4\,\pi^3+$$
$$148736\,C_2\,L^5\,b^5\,h^3\,t^4\,\pi^3+74368\,C_2\,L^5\,b^6\,h^2\,t^4\,\pi^3+2740608\,C_3\,L^5\,b^2\,h^7\,t^3\,\pi^2+6394752\,C_3\,L^5\,b^3\,h^6\,t^3\,\pi^2+$$
$$9592128\,C_3\,L^5\,b^4\,h^5\,t^3\,\pi^2+9592128\,C_3\,L^5\,b^5\,h^4\,t^3\,\pi^2+6394752\,C_3\,L^5\,b^6\,h^3\,t^3\,\pi^2+$$
$$2740608\,C_3\,L^5\,b^7\,h^2\,t^3\,\pi^2+1193808\,C_3\,L^5\,b^2\,h^6\,t^4\,\pi^3+2387616\,C_3\,L^5\,b^3\,h^5\,t^4\,\pi^3+$$
$$2984520\,C_3\,L^5\,b^4\,h^4\,t^4\,\pi^3+2387616\,C_3\,L^5\,b^5\,h^3\,t^4\,\pi^3+1193808\,C_3\,L^5\,b^6\,h^2\,t^4\,\pi^3+$$
$$156\,C_1\,L^5\,b^8\,{r_0}^2\,t^2\,\pi^3+15936\,C_2\,L^5\,b^8\,{r_0}^2\,t^2\,\pi^3+255816\,C_3\,L^5\,b^8\,{r_0}^2\,t^2\,\pi^3+156\,C_1\,L^5\,h^8\,{r_0}^2\,t^2\,\pi^3+$$
$$15936\,C_2\,L^5\,h^8\,{r_0}^2\,t^2\,\pi^3+255816\,C_3\,L^5\,h^8\,{r_0}^2\,t^2\,\pi^3+2400\,\pi\,C_1\,L^5\,b\,h^9\,t^2+2400\,\pi\,C_1\,L^5\,b^9\,h\,t^2+$$
$$96000\,\pi\,C_2\,L^5\,b\,h^9\,t^2+96000\,\pi\,C_2\,L^5\,b^9\,h\,t^2+843840\,\pi\,C_3\,L^5\,b\,h^9\,t^2+843840\,\pi\,C_3\,L^5\,b^9\,h\,t^2+$$

$10800\,\pi\,C_1\,L^5\,b^2\,h^8\,t^2+28800\,\pi\,C_1\,L^5\,b^3\,h^7\,t^2+50400\,\pi\,C_1\,L^5\,b^4\,h^6\,t^2+60480\,\pi\,C_1\,L^5\,b^5\,h^5\,t^2+$
$50400\,\pi\,C_1\,L^5\,b^6\,h^4\,t^2+28800\,\pi\,C_1\,L^5\,b^7\,h^3\,t^2+10800\,\pi\,C_1\,L^5\,b^8\,h^2\,t^2+1008\,C_1\,L^5\,b\,h^8\,t^3\,\pi^2+$
$1008\,C_1\,L^5\,b^8\,h\,t^3\,\pi^2+432000\,\pi\,C_2\,L^5\,b^2\,h^8\,t^2+1152000\,\pi\,C_2\,L^5\,b^3\,h^7\,t^2+2016000\,\pi\,C_2\,L^5\,b^4\,h^6\,t^2+$
$2419200\,\pi\,C_2\,L^5\,b^5\,h^5\,t^2+2016000\,\pi\,C_2\,L^5\,b^6\,h^4\,t^2+1152000\,\pi\,C_2\,L^5\,b^7\,h^3\,t^2+432000\,\pi\,C_2\,L^5\,b^8\,h^2\,t^2+$
$208\,C_1\,L^5\,b\,h^7\,t^4\,\pi^3+208\,C_1\,L^5\,b^7\,h\,t^4\,\pi^3+59328\,C_2\,L^5\,b\,h^8\,t^3\,\pi^2+59328\,C_2\,L^5\,b^8\,h\,t^3\,\pi^2+$
$3797280\,\pi\,C_3\,L^5\,b^2\,h^8\,t^2+10126080\,\pi\,C_3\,L^5\,b^3\,h^7\,t^2+17720640\,\pi\,C_3\,L^5\,b^4\,h^6\,t^2+$
$21264768\,\pi\,C_3\,L^5\,b^5\,h^5\,t^2+17720640\,\pi\,C_3\,L^5\,b^6\,h^4\,t^2+10126080\,\pi\,C_3\,L^5\,b^7\,h^3\,t^2+$
$3797280\,\pi\,C_3\,L^5\,b^8\,h^2\,t^2+21248\,C_2\,L^5\,b\,h^7\,t^4\,\pi^3+21248\,C_2\,L^5\,b^7\,h\,t^4\,\pi^3+685152\,C_3\,L^5\,b\,h^8\,t^3\,\pi^2+$
$685152\,C_3\,L^5\,b^8\,h\,t^3\,\pi^2+341088\,C_3\,L^5\,b\,h^7\,t^4\,\pi^3+341088\,C_3\,L^5\,b^7\,h\,t^4\,\pi^3+21600\,\pi\,C_1\,L^5\,b^2\,h^8\,r_0\,t+$
$57600\,\pi\,C_1\,L^5\,b^3\,h^7\,r_0\,t+100800\,\pi\,C_1\,L^5\,b^4\,h^6\,r_0\,t+120960\,\pi\,C_1\,L^5\,b^5\,h^5\,r_0\,t+100800\,\pi\,C_1\,L^5\,b^6\,h^4\,r_0\,t+$
$57600\,\pi\,C_1\,L^5\,b^7\,h^3\,r_0\,t+21600\,\pi\,C_1\,L^5\,b^8\,h^2\,r_0\,t+864000\,\pi\,C_2\,L^5\,b^2\,h^8\,r_0\,t+2304000\,\pi\,C_2\,L^5\,b^3\,h^7\,r_0\,t+$
$4032000\,\pi\,C_2\,L^5\,b^4\,h^6\,r_0\,t+4838400\,\pi\,C_2\,L^5\,b^5\,h^5\,r_0\,t+4032000\,\pi\,C_2\,L^5\,b^6\,h^4\,r_0\,t+$
$2304000\,\pi\,C_2\,L^5\,b^7\,h^3\,r_0\,t+864000\,\pi\,C_2\,L^5\,b^8\,h^2\,r_0\,t+7594560\,\pi\,C_3\,L^5\,b^2\,h^8\,r_0\,t+$
$20252160\,\pi\,C_3\,L^5\,b^3\,h^7\,r_0\,t+35441280\,\pi\,C_3\,L^5\,b^4\,h^6\,r_0\,t+42529536\,\pi\,C_3\,L^5\,b^5\,h^5\,r_0\,t+$
$35441280\,\pi\,C_3\,L^5\,b^6\,h^4\,r_0\,t+20252160\,\pi\,C_3\,L^5\,b^7\,h^3\,r_0\,t+7594560\,\pi\,C_3\,L^5\,b^8\,h^2\,r_0\,t+$
$4368\,C_1\,L^5\,b^2\,h^6\,{r_0}^2\,t^2\,\pi^3+8736\,C_1\,L^5\,b^3\,h^5\,{r_0}^2\,t^2\,\pi^3+10920\,C_1\,L^5\,b^4\,h^4\,{r_0}^2\,t^2\,\pi^3+$
$8736\,C_1\,L^5\,b^5\,h^3\,{r_0}^2\,t^2\,\pi^3+4368\,C_1\,L^5\,b^6\,h^2\,{r_0}^2\,t^2\,\pi^3+446208\,C_2\,L^5\,b^2\,h^6\,{r_0}^2\,t^2\,\pi^3+$
$892416\,C_2\,L^5\,b^3\,h^5\,{r_0}^2\,t^2\,\pi^3+1115520\,C_2\,L^5\,b^4\,h^4\,{r_0}^2\,t^2\,\pi^3+892416\,C_2\,L^5\,b^5\,h^3\,{r_0}^2\,t^2\,\pi^3+$
$446208\,C_2\,L^5\,b^6\,h^2\,{r_0}^2\,t^2\,\pi^3+7162848\,C_3\,L^5\,b^2\,h^6\,{r_0}^2\,t^2\,\pi^3+14325696\,C_3\,L^5\,b^3\,h^5\,{r_0}^2\,t^2\,\pi^3+$
$17907120\,C_3\,L^5\,b^4\,h^4\,{r_0}^2\,t^2\,\pi^3+14325696\,C_3\,L^5\,b^5\,h^3\,{r_0}^2\,t^2\,\pi^3+7162848\,C_3\,L^5\,b^6\,h^2\,{r_0}^2\,t^2\,\pi^3+$
$3024\,C_1\,L^5\,b\,h^8\,r_0\,t^2\,\pi^2+3024\,C_1\,L^5\,b\,h^8\,{r_0}^2\,t\,\pi^2+3024\,C_1\,L^5\,b^8\,h\,r_0\,t^2\,\pi^2+3024\,C_1\,L^5\,b^8\,h\,{r_0}^2\,t\,\pi^2+$
$832\,C_1\,L^5\,b\,h^7\,r_0\,t^3\,\pi^3+832\,C_1\,L^5\,b\,h^7\,{r_0}^3\,t\,\pi^3+832\,C_1\,L^5\,b^7\,h\,r_0\,t^3\,\pi^3+832\,C_1\,L^5\,b^7\,h\,{r_0}^3\,t\,\pi^3+$
$177984\,C_2\,L^5\,b\,h^8\,r_0\,t^2\,\pi^2+177984\,C_2\,L^5\,b\,h^8\,{r_0}^2\,t\,\pi^2+177984\,C_2\,L^5\,b^8\,h\,r_0\,t^2\,\pi^2+$
$177984\,C_2\,L^5\,b^8\,h\,{r_0}^2\,t\,\pi^2+84992\,C_2\,L^5\,b\,h^7\,r_0\,t^3\,\pi^3+84992\,C_2\,L^5\,b\,h^7\,{r_0}^3\,t\,\pi^3+$
$84992\,C_2\,L^5\,b^7\,h\,r_0\,t^3\,\pi^3+84992\,C_2\,L^5\,b^7\,h\,{r_0}^3\,t\,\pi^3+2055456\,C_3\,L^5\,b\,h^8\,r_0\,t^2\,\pi^2+$
$2055456\,C_3\,L^5\,b\,h^8\,{r_0}^2\,t\,\pi^2+2055456\,C_3\,L^5\,b^8\,h\,r_0\,t^2\,\pi^2+2055456\,C_3\,L^5\,b^8\,h\,{r_0}^2\,t\,\pi^2+$
$1364352\,C_3\,L^5\,b\,h^7\,r_0\,t^3\,\pi^3+1364352\,C_3\,L^5\,b\,h^7\,{r_0}^3\,t\,\pi^3+1364352\,C_3\,L^5\,b^7\,h\,r_0\,t^3\,\pi^3+$
$1364352\,C_3\,L^5\,b^7\,h\,{r_0}^3\,t\,\pi^3+4800\,\pi\,C_1\,L^5\,b\,h^9\,r_0\,t+4800\,\pi\,C_1\,L^5\,b^9\,h\,r_0\,t+192000\,\pi\,C_2\,L^5\,b\,h^9\,r_0\,t+$
$192000\,\pi\,C_2\,L^5\,b^9\,h\,r_0\,t+1687680\,\pi\,C_3\,L^5\,b\,h^9\,r_0\,t+1687680\,\pi\,C_3\,L^5\,b^9\,h\,r_0\,t+12096\,C_1\,L^5\,b^2\,h^7\,r_0\,t^2\,\pi^2+$
$12096\,C_1\,L^5\,b^2\,h^7\,{r_0}^2\,t\,\pi^2+28224\,C_1\,L^5\,b^3\,h^6\,r_0\,t^2\,\pi^2+28224\,C_1\,L^5\,b^3\,h^6\,{r_0}^2\,t\,\pi^2+$
$42336\,C_1\,L^5\,b^4\,h^5\,r_0\,t^2\,\pi^2+42336\,C_1\,L^5\,b^4\,h^5\,{r_0}^2\,t\,\pi^2+42336\,C_1\,L^5\,b^5\,h^4\,r_0\,t^2\,\pi^2+$
$42336\,C_1\,L^5\,b^5\,h^4\,{r_0}^2\,t\,\pi^2+28224\,C_1\,L^5\,b^6\,h^3\,r_0\,t^2\,\pi^2+28224\,C_1\,L^5\,b^6\,h^3\,{r_0}^2\,t\,\pi^2+$
$12096\,C_1\,L^5\,b^7\,h^2\,r_0\,t^2\,\pi^2+12096\,C_1\,L^5\,b^7\,h^2\,{r_0}^2\,t\,\pi^2+1248\,C_1\,L^5\,b\,h^7\,{r_0}^2\,t^2\,\pi^3+$
$2912\,C_1\,L^5\,b^2\,h^6\,r_0\,t^3\,\pi^3+2912\,C_1\,L^5\,b^2\,h^6\,{r_0}^3\,t\,\pi^3+5824\,C_1\,L^5\,b^3\,h^5\,r_0\,t^3\,\pi^3+$
$5824\,C_1\,L^5\,b^3\,h^5\,{r_0}^3\,t\,\pi^3+7280\,C_1\,L^5\,b^4\,h^4\,r_0\,t^3\,\pi^3+7280\,C_1\,L^5\,b^4\,h^4\,{r_0}^3\,t\,\pi^3+$
$5824\,C_1\,L^5\,b^5\,h^3\,r_0\,t^3\,\pi^3+5824\,C_1\,L^5\,b^5\,h^3\,{r_0}^3\,t\,\pi^3+2912\,C_1\,L^5\,b^6\,h^2\,r_0\,t^3\,\pi^3+$
$2912\,C_1\,L^5\,b^6\,h^2\,{r_0}^3\,t\,\pi^3+1248\,C_1\,L^5\,b^7\,h\,{r_0}^2\,t^2\,\pi^3+711936\,C_2\,L^5\,b^2\,h^7\,r_0\,t^2\,\pi^2+$
$711936\,C_2\,L^5\,b^2\,h^7\,{r_0}^2\,t\,\pi^2+1661184\,C_2\,L^5\,b^3\,h^6\,r_0\,t^2\,\pi^2+1661184\,C_2\,L^5\,b^3\,h^6\,{r_0}^2\,t\,\pi^2+$
$2491776\,C_2\,L^5\,b^4\,h^5\,r_0\,t^2\,\pi^2+2491776\,C_2\,L^5\,b^4\,h^5\,{r_0}^2\,t\,\pi^2+2491776\,C_2\,L^5\,b^5\,h^4\,r_0\,t^2\,\pi^2+$
$2491776\,C_2\,L^5\,b^5\,h^4\,{r_0}^2\,t\,\pi^2+1661184\,C_2\,L^5\,b^6\,h^3\,r_0\,t^2\,\pi^2+1661184\,C_2\,L^5\,b^6\,h^3\,{r_0}^2\,t\,\pi^2+$
$711936\,C_2\,L^5\,b^7\,h^2\,r_0\,t^2\,\pi^2+711936\,C_2\,L^5\,b^7\,h^2\,{r_0}^2\,t\,\pi^2+127488\,C_2\,L^5\,b\,h^7\,{r_0}^2\,t^2\,\pi^3+$
$297472\,C_2\,L^5\,b^2\,h^6\,r_0\,t^3\,\pi^3+297472\,C_2\,L^5\,b^2\,h^6\,{r_0}^3\,t\,\pi^3+594944\,C_2\,L^5\,b^3\,h^5\,r_0\,t^3\,\pi^3+$
$594944\,C_2\,L^5\,b^3\,h^5\,{r_0}^3\,t\,\pi^3+743680\,C_2\,L^5\,b^4\,h^4\,r_0\,t^3\,\pi^3+743680\,C_2\,L^5\,b^4\,h^4\,{r_0}^3\,t\,\pi^3+$
$594944\,C_2\,L^5\,b^5\,h^3\,r_0\,t^3\,\pi^3+594944\,C_2\,L^5\,b^5\,h^3\,{r_0}^3\,t\,\pi^3+297472\,C_2\,L^5\,b^6\,h^2\,r_0\,t^3\,\pi^3+$
$297472\,C_2\,L^5\,b^6\,h^2\,{r_0}^3\,t\,\pi^3+127488\,C_2\,L^5\,b^7\,h\,{r_0}^2\,t^2\,\pi^3+8221824\,C_3\,L^5\,b^2\,h^7\,r_0\,t^2\,\pi^2+$
$8221824\,C_3\,L^5\,b^2\,h^7\,{r_0}^2\,t\,\pi^2+19184256\,C_3\,L^5\,b^3\,h^6\,r_0\,t^2\,\pi^2+19184256\,C_3\,L^5\,b^3\,h^6\,{r_0}^2\,t\,\pi^2+$

$28776384\,C_3\,L^5\,b^4\,h^5\,r_0\,t^2\,\pi^2+28776384\,C_3\,L^5\,b^4\,h^5\,{r_0}^2\,t\,\pi^2+28776384\,C_3\,L^5\,b^5\,h^4\,r_0\,t^2\,\pi^2+$
$28776384\,C_3\,L^5\,b^5\,h^4\,{r_0}^2\,t\,\pi^2+19184256\,C_3\,L^5\,b^6\,h^3\,r_0\,t^2\,\pi^2+19184256\,C_3\,L^5\,b^6\,h^3\,{r_0}^2\,t\,\pi^2+$
$8221824\,C_3\,L^5\,b^7\,h^2\,r_0\,t^2\,\pi^2+8221824\,C_3\,L^5\,b^7\,h^2\,{r_0}^2\,t\,\pi^2+2046528\,C_3\,L^5\,b\,h^7\,{r_0}^2\,t^2\,\pi^3+$
$4775232\,C_3\,L^5\,b^2\,h^6\,r_0\,t^3\,\pi^3+4775232\,C_3\,L^5\,b^2\,h^6\,{r_0}^3\,t\,\pi^3+9550464\,C_3\,L^5\,b^3\,h^5\,r_0\,t^3\,\pi^3+$
$9550464\,C_3\,L^5\,b^3\,h^5\,{r_0}^3\,t\,\pi^3+11938080\,C_3\,L^5\,b^4\,h^4\,r_0\,t^3\,\pi^3+11938080\,C_3\,L^5\,b^4\,h^4\,{r_0}^3\,t\,\pi^3+$
$9550464\,C_3\,L^5\,b^5\,h^3\,r_0\,t^3\,\pi^3+9550464\,C_3\,L^5\,b^5\,h^3\,{r_0}^3\,t\,\pi^3+4775232\,C_3\,L^5\,b^6\,h^2\,r_0\,t^3\,\pi^3+$
$4775232\,C_3\,L^5\,b^6\,h^2\,{r_0}^3\,t\,\pi^3+2046528\,C_3\,L^5\,b^7\,h\,{r_0}^2\,t^2\,\pi^3$

$\mathrm{a}_{t,8}=1080\,\pi\,C_1\,L^6\,b^9\,t^2+19440\,\pi\,C_2\,L^6\,b^9\,t^2+89856\,\pi\,C_3\,L^6\,b^9\,t^2+1080\,\pi\,C_1\,L^6\,h^9\,t^2+$
$19440\,\pi\,C_2\,L^6\,h^9\,t^2+89856\,\pi\,C_3\,L^6\,h^9\,t^2+468\,C_1\,L^6\,b^8\,t^3\,\pi^2+100\,C_1\,L^6\,b^7\,t^4\,\pi^3+$
$12648\,C_2\,L^6\,b^8\,t^3\,\pi^2+4760\,C_2\,L^6\,b^7\,t^4\,\pi^3+79488\,C_3\,L^6\,b^8\,t^3\,\pi^2+43008\,C_3\,L^6\,b^7\,t^4\,\pi^3+$
$468\,C_1\,L^6\,h^8\,t^3\,\pi^2+100\,C_1\,L^6\,h^7\,t^4\,\pi^3+12648\,C_2\,L^6\,h^8\,t^3\,\pi^2+4760\,C_2\,L^6\,h^7\,t^4\,\pi^3+$
$79488\,C_3\,L^6\,h^8\,t^3\,\pi^2+43008\,C_3\,L^6\,h^7\,t^4\,\pi^3+1404\,C_1\,L^6\,b^8\,r_0\,t^2\,\pi^2+1404\,C_1\,L^6\,b^8\,{r_0}^2\,t\,\pi^2+$
$400\,C_1\,L^6\,b^7\,r_0\,t^3\,\pi^3+400\,C_1\,L^6\,b^7\,{r_0}^3\,t\,\pi^3+37944\,C_2\,L^6\,b^8\,r_0\,t^2\,\pi^2+37944\,C_2\,L^6\,b^8\,{r_0}^2\,t\,\pi^2+$
$19040\,C_2\,L^6\,b^7\,r_0\,t^3\,\pi^3+19040\,C_2\,L^6\,b^7\,{r_0}^3\,t\,\pi^3+238464\,C_3\,L^6\,b^8\,r_0\,t^2\,\pi^2+238464\,C_3\,L^6\,b^8\,{r_0}^2\,t\,\pi^2+$
$172032\,C_3\,L^6\,b^7\,r_0\,t^3\,\pi^3+172032\,C_3\,L^6\,b^7\,{r_0}^3\,t\,\pi^3+1404\,C_1\,L^6\,h^8\,r_0\,t^2\,\pi^2+1404\,C_1\,L^6\,h^8\,{r_0}^2\,t\,\pi^2+$
$400\,C_1\,L^6\,h^7\,r_0\,t^3\,\pi^3+400\,C_1\,L^6\,h^7\,{r_0}^3\,t\,\pi^3+37944\,C_2\,L^6\,h^8\,r_0\,t^2\,\pi^2+37944\,C_2\,L^6\,h^8\,{r_0}^2\,t\,\pi^2+$
$19040\,C_2\,L^6\,h^7\,r_0\,t^3\,\pi^3+19040\,C_2\,L^6\,h^7\,{r_0}^3\,t\,\pi^3+238464\,C_3\,L^6\,h^8\,r_0\,t^2\,\pi^2+238464\,C_3\,L^6\,h^8\,{r_0}^2\,t\,\pi^2+$
$172032\,C_3\,L^6\,h^7\,r_0\,t^3\,\pi^3+172032\,C_3\,L^6\,h^7\,{r_0}^3\,t\,\pi^3+2160\,\pi\,C_1\,L^6\,b^9\,r_0\,t+38880\,\pi\,C_2\,L^6\,b^9\,r_0\,t+$
$179712\,\pi\,C_3\,L^6\,b^9\,r_0\,t+2160\,\pi\,C_1\,L^6\,h^9\,r_0\,t+38880\,\pi\,C_2\,L^6\,h^9\,r_0\,t+179712\,\pi\,C_3\,L^6\,h^9\,r_0\,t+$
$13104\,C_1\,L^6\,b^2\,h^6\,t^3\,\pi^2+26208\,C_1\,L^6\,b^3\,h^5\,t^3\,\pi^2+32760\,C_1\,L^6\,b^4\,h^4\,t^3\,\pi^2+26208\,C_1\,L^6\,b^5\,h^3\,t^3\,\pi^2+$
$13104\,C_1\,L^6\,b^6\,h^2\,t^3\,\pi^2+2100\,C_1\,L^6\,b^2\,h^5\,t^4\,\pi^3+3500\,C_1\,L^6\,b^3\,h^4\,t^4\,\pi^3+3500\,C_1\,L^6\,b^4\,h^3\,t^4\,\pi^3+$
$2100\,C_1\,L^6\,b^5\,h^2\,t^4\,\pi^3+354144\,C_2\,L^6\,b^2\,h^6\,t^3\,\pi^2+708288\,C_2\,L^6\,b^3\,h^5\,t^3\,\pi^2+885360\,C_2\,L^6\,b^4\,h^4\,t^3\,\pi^2+$
$708288\,C_2\,L^6\,b^5\,h^3\,t^3\,\pi^2+354144\,C_2\,L^6\,b^6\,h^2\,t^3\,\pi^2+99960\,C_2\,L^6\,b^2\,h^5\,t^4\,\pi^3+166600\,C_2\,L^6\,b^3\,h^4\,t^4\,\pi^3+$
$166600\,C_2\,L^6\,b^4\,h^3\,t^4\,\pi^3+99960\,C_2\,L^6\,b^5\,h^2\,t^4\,\pi^3+2225664\,C_3\,L^6\,b^2\,h^6\,t^3\,\pi^2+4451328\,C_3\,L^6\,b^3\,h^5\,t^3\,\pi^2+$
$5564160\,C_3\,L^6\,b^4\,h^4\,t^3\,\pi^2+4451328\,C_3\,L^6\,b^5\,h^3\,t^3\,\pi^2+2225664\,C_3\,L^6\,b^6\,h^2\,t^3\,\pi^2+$
$903168\,C_3\,L^6\,b^2\,h^5\,t^4\,\pi^3+1505280\,C_3\,L^6\,b^3\,h^4\,t^4\,\pi^3+1505280\,C_3\,L^6\,b^4\,h^3\,t^4\,\pi^3+$
$903168\,C_3\,L^6\,b^5\,h^2\,t^4\,\pi^3+600\,C_1\,L^6\,b^7\,{r_0}^2\,t^2\,\pi^3+28560\,C_2\,L^6\,b^7\,{r_0}^2\,t^2\,\pi^3+258048\,C_3\,L^6\,b^7\,{r_0}^2\,t^2\,\pi^3+$
$600\,C_1\,L^6\,h^7\,{r_0}^2\,t^2\,\pi^3+28560\,C_2\,L^6\,h^7\,{r_0}^2\,t^2\,\pi^3+258048\,C_3\,L^6\,h^7\,{r_0}^2\,t^2\,\pi^3+9720\,\pi\,C_1\,L^6\,b\,h^8\,t^2+$
$9720\,\pi\,C_1\,L^6\,b^8\,h\,t^2+174960\,\pi\,C_2\,L^6\,b\,h^8\,t^2+174960\,\pi\,C_2\,L^6\,b^8\,h\,t^2+808704\,\pi\,C_3\,L^6\,b\,h^8\,t^2+$
$808704\,\pi\,C_3\,L^6\,b^8\,h\,t^2+38880\,\pi\,C_1\,L^6\,b^2\,h^7\,t^2+90720\,\pi\,C_1\,L^6\,b^3\,h^6\,t^2+136080\,\pi\,C_1\,L^6\,b^4\,h^5\,t^2+$
$136080\,\pi\,C_1\,L^6\,b^5\,h^4\,t^2+90720\,\pi\,C_1\,L^6\,b^6\,h^3\,t^2+38880\,\pi\,C_1\,L^6\,b^7\,h^2\,t^2+3744\,C_1\,L^6\,b\,h^7\,t^3\,\pi^2+$
$3744\,C_1\,L^6\,b^7\,h\,t^3\,\pi^2+699840\,\pi\,C_2\,L^6\,b^2\,h^7\,t^2+1632960\,\pi\,C_2\,L^6\,b^3\,h^6\,t^2+2449440\,\pi\,C_2\,L^6\,b^4\,h^5\,t^2+$
$2449440\,\pi\,C_2\,L^6\,b^5\,h^4\,t^2+1632960\,\pi\,C_2\,L^6\,b^6\,h^3\,t^2+699840\,\pi\,C_2\,L^6\,b^7\,h^2\,t^2+700\,C_1\,L^6\,b\,h^6\,t^4\,\pi^3+$
$700\,C_1\,L^6\,b^6\,h\,t^4\,\pi^3+101184\,C_2\,L^6\,b\,h^7\,t^3\,\pi^2+101184\,C_2\,L^6\,b^7\,h\,t^3\,\pi^2+3234816\,\pi\,C_3\,L^6\,b^2\,h^7\,t^2+$
$7547904\,\pi\,C_3\,L^6\,b^3\,h^6\,t^2+11321856\,\pi\,C_3\,L^6\,b^4\,h^5\,t^2+11321856\,\pi\,C_3\,L^6\,b^5\,h^4\,t^2+$
$7547904\,\pi\,C_3\,L^6\,b^6\,h^3\,t^2+3234816\,\pi\,C_3\,L^6\,b^7\,h^2\,t^2+33320\,C_2\,L^6\,b\,h^6\,t^4\,\pi^3+33320\,C_2\,L^6\,b^6\,h\,t^4\,\pi^3+$
$635904\,C_3\,L^6\,b\,h^7\,t^3\,\pi^2+635904\,C_3\,L^6\,b^7\,h\,t^3\,\pi^2+301056\,C_3\,L^6\,b\,h^6\,t^4\,\pi^3+301056\,C_3\,L^6\,b^6\,h\,t^4\,\pi^3+$
$77760\,\pi\,C_1\,L^6\,b^2\,h^7\,r_0\,t+181440\,\pi\,C_1\,L^6\,b^3\,h^6\,r_0\,t+272160\,\pi\,C_1\,L^6\,b^4\,h^5\,r_0\,t+272160\,\pi\,C_1\,L^6\,b^5\,h^4\,r_0\,t+$
$181440\,\pi\,C_1\,L^6\,b^6\,h^3\,r_0\,t+77760\,\pi\,C_1\,L^6\,b^7\,h^2\,r_0\,t+1399680\,\pi\,C_2\,L^6\,b^2\,h^7\,r_0\,t+$
$3265920\,\pi\,C_2\,L^6\,b^3\,h^6\,r_0\,t+4898880\,\pi\,C_2\,L^6\,b^4\,h^5\,r_0\,t+4898880\,\pi\,C_2\,L^6\,b^5\,h^4\,r_0\,t+$
$3265920\,\pi\,C_2\,L^6\,b^6\,h^3\,r_0\,t+1399680\,\pi\,C_2\,L^6\,b^7\,h^2\,r_0\,t+6469632\,\pi\,C_3\,L^6\,b^2\,h^7\,r_0\,t+$
$15095808\,\pi\,C_3\,L^6\,b^3\,h^6\,r_0\,t+22643712\,\pi\,C_3\,L^6\,b^4\,h^5\,r_0\,t+22643712\,\pi\,C_3\,L^6\,b^5\,h^4\,r_0\,t+$
$15095808\,\pi\,C_3\,L^6\,b^6\,h^3\,r_0\,t+6469632\,\pi\,C_3\,L^6\,b^7\,h^2\,r_0\,t+12600\,C_1\,L^6\,b^2\,h^5\,{r_0}^2\,t^2\,\pi^3+$
$21000\,C_1\,L^6\,b^3\,h^4\,{r_0}^2\,t^2\,\pi^3+21000\,C_1\,L^6\,b^4\,h^3\,{r_0}^2\,t^2\,\pi^3+12600\,C_1\,L^6\,b^5\,h^2\,{r_0}^2\,t^2\,\pi^3+$
$599760\,C_2\,L^6\,b^2\,h^5\,{r_0}^2\,t^2\,\pi^3+999600\,C_2\,L^6\,b^3\,h^4\,{r_0}^2\,t^2\,\pi^3+999600\,C_2\,L^6\,b^4\,h^3\,{r_0}^2\,t^2\,\pi^3+$

$599760\,C_2\,L^6\,b^5\,h^2\,{r_0}^2\,t^2\,\pi^3+5419008\,C_3\,L^6\,b^2\,h^5\,{r_0}^2\,t^2\,\pi^3+9031680\,C_3\,L^6\,b^3\,h^4\,{r_0}^2\,t^2\,\pi^3+$
$9031680\,C_3\,L^6\,b^4\,h^3\,{r_0}^2\,t^2\,\pi^3+5419008\,C_3\,L^6\,b^5\,h^2\,{r_0}^2\,t^2\,\pi^3+11232\,C_1\,L^6\,b\,h^7\,r_0\,t^2\,\pi^2+$
$11232\,C_1\,L^6\,b\,h^7\,{r_0}^2\,t\,\pi^2+11232\,C_1\,L^6\,b^7\,h\,r_0\,t^2\,\pi^2+11232\,C_1\,L^6\,b^7\,h\,{r_0}^2\,t\,\pi^2+$
$2800\,C_1\,L^6\,b\,h^6\,r_0\,t^3\,\pi^3+2800\,C_1\,L^6\,b\,h^6\,{r_0}^3\,t\,\pi^3+2800\,C_1\,L^6\,b^6\,h\,r_0\,t^3\,\pi^3+2800\,C_1\,L^6\,b^6\,h\,{r_0}^3\,t\,\pi^3+$
$303552\,C_2\,L^6\,b\,h^7\,r_0\,t^2\,\pi^2+303552\,C_2\,L^6\,b\,h^7\,{r_0}^2\,t\,\pi^2+303552\,C_2\,L^6\,b^7\,h\,r_0\,t^2\,\pi^2+$
$303552\,C_2\,L^6\,b^7\,h\,{r_0}^2\,t\,\pi^2+133280\,C_2\,L^6\,b\,h^6\,r_0\,t^3\,\pi^3+133280\,C_2\,L^6\,b\,h^6\,{r_0}^3\,t\,\pi^3+$
$133280\,C_2\,L^6\,b^6\,h\,r_0\,t^3\,\pi^3+133280\,C_2\,L^6\,b^6\,h\,{r_0}^3\,t\,\pi^3+1907712\,C_3\,L^6\,b\,h^7\,r_0\,t^2\,\pi^2+$
$1907712\,C_3\,L^6\,b\,h^7\,{r_0}^2\,t\,\pi^2+1907712\,C_3\,L^6\,b^7\,h\,r_0\,t^2\,\pi^2+1907712\,C_3\,L^6\,b^7\,h\,{r_0}^2\,t\,\pi^2+$
$1204224\,C_3\,L^6\,b\,h^6\,r_0\,t^3\,\pi^3+1204224\,C_3\,L^6\,b\,h^6\,{r_0}^3\,t\,\pi^3+1204224\,C_3\,L^6\,b^6\,h\,r_0\,t^3\,\pi^3+$
$1204224\,C_3\,L^6\,b^6\,h\,{r_0}^3\,t\,\pi^3+19440\,\pi\,C_1\,L^6\,b\,h^8\,r_0\,t+19440\,\pi\,C_1\,L^6\,b^8\,h\,r_0\,t+349920\,\pi\,C_2\,L^6\,b\,h^8\,r_0\,t+$
$349920\,\pi\,C_2\,L^6\,b^8\,h\,r_0\,t+1617408\,\pi\,C_3\,L^6\,b\,h^8\,r_0\,t+1617408\,\pi\,C_3\,L^6\,b^8\,h\,r_0\,t+39312\,C_1\,L^6\,b^2\,h^6\,r_0\,t^2\,\pi^2+$
$39312\,C_1\,L^6\,b^2\,h^6\,{r_0}^2\,t\,\pi^2+78624\,C_1\,L^6\,b^3\,h^5\,r_0\,t^2\,\pi^2+78624\,C_1\,L^6\,b^3\,h^5\,{r_0}^2\,t\,\pi^2+$
$98280\,C_1\,L^6\,b^4\,h^4\,r_0\,t^2\,\pi^2+98280\,C_1\,L^6\,b^4\,h^4\,{r_0}^2\,t\,\pi^2+78624\,C_1\,L^6\,b^5\,h^3\,r_0\,t^2\,\pi^2+$
$78624\,C_1\,L^6\,b^5\,h^3\,{r_0}^2\,t\,\pi^2+39312\,C_1\,L^6\,b^6\,h^2\,r_0\,t^2\,\pi^2+39312\,C_1\,L^6\,b^6\,h^2\,{r_0}^2\,t\,\pi^2+$
$4200\,C_1\,L^6\,b\,h^6\,{r_0}^2\,t^2\,\pi^3+8400\,C_1\,L^6\,b^2\,h^5\,r_0\,t^3\,\pi^3+8400\,C_1\,L^6\,b^2\,h^5\,{r_0}^3\,t\,\pi^3+$
$14000\,C_1\,L^6\,b^3\,h^4\,r_0\,t^3\,\pi^3+14000\,C_1\,L^6\,b^3\,h^4\,{r_0}^3\,t\,\pi^3+14000\,C_1\,L^6\,b^4\,h^3\,r_0\,t^3\,\pi^3+$
$14000\,C_1\,L^6\,b^4\,h^3\,{r_0}^3\,t\,\pi^3+8400\,C_1\,L^6\,b^5\,h^2\,r_0\,t^3\,\pi^3+8400\,C_1\,L^6\,b^5\,h^2\,{r_0}^3\,t\,\pi^3+$
$4200\,C_1\,L^6\,b^6\,h\,{r_0}^2\,t^2\,\pi^3+1062432\,C_2\,L^6\,b^2\,h^6\,r_0\,t^2\,\pi^2+1062432\,C_2\,L^6\,b^2\,h^6\,{r_0}^2\,t\,\pi^2+$
$2124864\,C_2\,L^6\,b^3\,h^5\,r_0\,t^2\,\pi^2+2124864\,C_2\,L^6\,b^3\,h^5\,{r_0}^2\,t\,\pi^2+2656080\,C_2\,L^6\,b^4\,h^4\,r_0\,t^2\,\pi^2+$
$2656080\,C_2\,L^6\,b^4\,h^4\,{r_0}^2\,t\,\pi^2+2124864\,C_2\,L^6\,b^5\,h^3\,r_0\,t^2\,\pi^2+2124864\,C_2\,L^6\,b^5\,h^3\,{r_0}^2\,t\,\pi^2+$
$1062432\,C_2\,L^6\,b^6\,h^2\,r_0\,t^2\,\pi^2+1062432\,C_2\,L^6\,b^6\,h^2\,{r_0}^2\,t\,\pi^2+199920\,C_2\,L^6\,b\,h^6\,{r_0}^2\,t^2\,\pi^3+$
$399840\,C_2\,L^6\,b^2\,h^5\,r_0\,t^3\,\pi^3+399840\,C_2\,L^6\,b^2\,h^5\,{r_0}^3\,t\,\pi^3+666400\,C_2\,L^6\,b^3\,h^4\,r_0\,t^3\,\pi^3+$
$666400\,C_2\,L^6\,b^3\,h^4\,{r_0}^3\,t\,\pi^3+666400\,C_2\,L^6\,b^4\,h^3\,r_0\,t^3\,\pi^3+666400\,C_2\,L^6\,b^4\,h^3\,{r_0}^3\,t\,\pi^3+$
$399840\,C_2\,L^6\,b^5\,h^2\,r_0\,t^3\,\pi^3+399840\,C_2\,L^6\,b^5\,h^2\,{r_0}^3\,t\,\pi^3+199920\,C_2\,L^6\,b^6\,h\,{r_0}^2\,t^2\,\pi^3+$
$6676992\,C_3\,L^6\,b^2\,h^6\,r_0\,t^2\,\pi^2+6676992\,C_3\,L^6\,b^2\,h^6\,{r_0}^2\,t\,\pi^2+13353984\,C_3\,L^6\,b^3\,h^5\,r_0\,t^2\,\pi^2+$
$13353984\,C_3\,L^6\,b^3\,h^5\,{r_0}^2\,t\,\pi^2+16692480\,C_3\,L^6\,b^4\,h^4\,r_0\,t^2\,\pi^2+16692480\,C_3\,L^6\,b^4\,h^4\,{r_0}^2\,t\,\pi^2+$
$13353984\,C_3\,L^6\,b^5\,h^3\,r_0\,t^2\,\pi^2+13353984\,C_3\,L^6\,b^5\,h^3\,{r_0}^2\,t\,\pi^2+6676992\,C_3\,L^6\,b^6\,h^2\,r_0\,t^2\,\pi^2+$
$6676992\,C_3\,L^6\,b^6\,h^2\,{r_0}^2\,t\,\pi^2+1806336\,C_3\,L^6\,b\,h^6\,{r_0}^2\,t^2\,\pi^3+3612672\,C_3\,L^6\,b^2\,h^5\,r_0\,t^3\,\pi^3+$
$3612672\,C_3\,L^6\,b^2\,h^5\,{r_0}^3\,t\,\pi^3+6021120\,C_3\,L^6\,b^3\,h^4\,r_0\,t^3\,\pi^3+6021120\,C_3\,L^6\,b^3\,h^4\,{r_0}^3\,t\,\pi^3+$
$6021120\,C_3\,L^6\,b^4\,h^3\,r_0\,t^3\,\pi^3+6021120\,C_3\,L^6\,b^4\,h^3\,{r_0}^3\,t\,\pi^3+3612672\,C_3\,L^6\,b^5\,h^2\,r_0\,t^3\,\pi^3+$
$3612672\,C_3\,L^6\,b^5\,h^2\,{r_0}^3\,t\,\pi^3+1806336\,C_3\,L^6\,b^6\,h\,{r_0}^2\,t^2\,\pi^3$

$\mathrm{a}_{t,7}=2880\,\pi\,C_1\,L^7\,b^8\,t^2+26496\,\pi\,C_2\,L^7\,b^8\,t^2+62208\,\pi\,C_3\,L^7\,b^8\,t^2+2880\,\pi\,C_1\,L^7\,h^8\,t^2+$
$26496\,\pi\,C_2\,L^7\,h^8\,t^2+62208\,\pi\,C_3\,L^7\,h^8\,t^2+1152\,C_1\,L^7\,b^7\,t^3\,\pi^2+224\,C_1\,L^7\,b^6\,t^4\,\pi^3+$
$16512\,C_2\,L^7\,b^7\,t^3\,\pi^2+5824\,C_2\,L^7\,b^6\,t^4\,\pi^3+55296\,C_3\,L^7\,b^7\,t^3\,\pi^2+29568\,C_3\,L^7\,b^6\,t^4\,\pi^3+$
$1152\,C_1\,L^7\,h^7\,t^3\,\pi^2+224\,C_1\,L^7\,h^6\,t^4\,\pi^3+16512\,C_2\,L^7\,h^7\,t^3\,\pi^2+5824\,C_2\,L^7\,h^6\,t^4\,\pi^3+$
$55296\,C_3\,L^7\,h^7\,t^3\,\pi^2+29568\,C_3\,L^7\,h^6\,t^4\,\pi^3+3456\,C_1\,L^7\,b^7\,r_0\,t^2\,\pi^2+3456\,C_1\,L^7\,b^7\,{r_0}^2\,t\,\pi^2+$
$896\,C_1\,L^7\,b^6\,r_0\,t^3\,\pi^3+896\,C_1\,L^7\,b^6\,{r_0}^3\,t\,\pi^3+49536\,C_2\,L^7\,b^7\,r_0\,t^2\,\pi^2+49536\,C_2\,L^7\,b^7\,{r_0}^2\,t\,\pi^2+$
$23296\,C_2\,L^7\,b^6\,r_0\,t^3\,\pi^3+23296\,C_2\,L^7\,b^6\,{r_0}^3\,t\,\pi^3+165888\,C_3\,L^7\,b^7\,r_0\,t^2\,\pi^2+165888\,C_3\,L^7\,b^7\,{r_0}^2\,t\,\pi^2+$
$118272\,C_3\,L^7\,b^6\,r_0\,t^3\,\pi^3+118272\,C_3\,L^7\,b^6\,{r_0}^3\,t\,\pi^3+3456\,C_1\,L^7\,h^7\,r_0\,t^2\,\pi^2+3456\,C_1\,L^7\,h^7\,{r_0}^2\,t\,\pi^2+$
$896\,C_1\,L^7\,h^6\,r_0\,t^3\,\pi^3+896\,C_1\,L^7\,h^6\,{r_0}^3\,t\,\pi^3+49536\,C_2\,L^7\,h^7\,r_0\,t^2\,\pi^2+49536\,C_2\,L^7\,h^7\,{r_0}^2\,t\,\pi^2+$
$23296\,C_2\,L^7\,h^6\,r_0\,t^3\,\pi^3+23296\,C_2\,L^7\,h^6\,{r_0}^3\,t\,\pi^3+165888\,C_3\,L^7\,h^7\,r_0\,t^2\,\pi^2+165888\,C_3\,L^7\,h^7\,{r_0}^2\,t\,\pi^2+$
$118272\,C_3\,L^7\,h^6\,r_0\,t^3\,\pi^3+118272\,C_3\,L^7\,h^6\,{r_0}^3\,t\,\pi^3+5760\,\pi\,C_1\,L^7\,b^8\,r_0\,t+52992\,\pi\,C_2\,L^7\,b^8\,r_0\,t+$
$124416\,\pi\,C_3\,L^7\,b^8\,r_0\,t+5760\,\pi\,C_1\,L^7\,h^8\,r_0\,t+52992\,\pi\,C_2\,L^7\,h^8\,r_0\,t+124416\,\pi\,C_3\,L^7\,h^8\,r_0\,t+$
$24192\,C_1\,L^7\,b^2\,h^5\,t^3\,\pi^2+40320\,C_1\,L^7\,b^3\,h^4\,t^3\,\pi^2+40320\,C_1\,L^7\,b^4\,h^3\,t^3\,\pi^2+24192\,C_1\,L^7\,b^5\,h^2\,t^3\,\pi^2+$
$3360\,C_1\,L^7\,b^2\,h^4\,t^4\,\pi^3+4480\,C_1\,L^7\,b^3\,h^3\,t^4\,\pi^3+3360\,C_1\,L^7\,b^4\,h^2\,t^4\,\pi^3+346752\,C_2\,L^7\,b^2\,h^5\,t^3\,\pi^2+$

$577920\,C_2\,L^7\,b^3\,h^4\,t^3\,\pi^2+577920\,C_2\,L^7\,b^4\,h^3\,t^3\,\pi^2+346752\,C_2\,L^7\,b^5\,h^2\,t^3\,\pi^2+87360\,C_2\,L^7\,b^2\,h^4\,t^4\,\pi^3+$
$116480\,C_2\,L^7\,b^3\,h^3\,t^4\,\pi^3+87360\,C_2\,L^7\,b^4\,h^2\,t^4\,\pi^3+1161216\,C_3\,L^7\,b^2\,h^5\,t^3\,\pi^2+1935360\,C_3\,L^7\,b^3\,h^4\,t^3\,\pi^2+$
$1935360\,C_3\,L^7\,b^4\,h^3\,t^3\,\pi^2+1161216\,C_3\,L^7\,b^5\,h^2\,t^3\,\pi^2+443520\,C_3\,L^7\,b^2\,h^4\,t^4\,\pi^3+$
$591360\,C_3\,L^7\,b^3\,h^3\,t^4\,\pi^3+443520\,C_3\,L^7\,b^4\,h^2\,t^4\,\pi^3+1344\,C_1\,L^7\,b^6\,{r_0}^2\,t^2\,\pi^3+34944\,C_2\,L^7\,b^6\,{r_0}^2\,t^2\,\pi^3+$
$177408\,C_3\,L^7\,b^6\,{r_0}^2\,t^2\,\pi^3+1344\,C_1\,L^7\,h^6\,{r_0}^2\,t^2\,\pi^3+34944\,C_2\,L^7\,h^6\,{r_0}^2\,t^2\,\pi^3+177408\,C_3\,L^7\,h^6\,{r_0}^2\,t^2\,\pi^3+$
$23040\,\pi\,C_1\,L^7\,b\,h^7\,t^2+23040\,\pi\,C_1\,L^7\,b^7\,h\,t^2+211968\,\pi\,C_2\,L^7\,b\,h^7\,t^2+211968\,\pi\,C_2\,L^7\,b^7\,h\,t^2+$
$497664\,\pi\,C_3\,L^7\,b\,h^7\,t^2+497664\,\pi\,C_3\,L^7\,b^7\,h\,t^2+80640\,\pi\,C_1\,L^7\,b^2\,h^6\,t^2+161280\,\pi\,C_1\,L^7\,b^3\,h^5\,t^2+$
$201600\,\pi\,C_1\,L^7\,b^4\,h^4\,t^2+161280\,\pi\,C_1\,L^7\,b^5\,h^3\,t^2+80640\,\pi\,C_1\,L^7\,b^6\,h^2\,t^2+8064\,C_1\,L^7\,b\,h^6\,t^3\,\pi^2+$
$8064\,C_1\,L^7\,b^6\,h\,t^3\,\pi^2+741888\,\pi\,C_2\,L^7\,b^2\,h^6\,t^2+1483776\,\pi\,C_2\,L^7\,b^3\,h^5\,t^2+1854720\,\pi\,C_2\,L^7\,b^4\,h^4\,t^2+$
$1483776\,\pi\,C_2\,L^7\,b^5\,h^3\,t^2+741888\,\pi\,C_2\,L^7\,b^6\,h^2\,t^2+1344\,C_1\,L^7\,b\,h^5\,t^4\,\pi^3+1344\,C_1\,L^7\,b^5\,h\,t^4\,\pi^3+$
$115584\,C_2\,L^7\,b\,h^6\,t^3\,\pi^2+115584\,C_2\,L^7\,b^6\,h\,t^3\,\pi^2+1741824\,\pi\,C_3\,L^7\,b^2\,h^6\,t^2+3483648\,\pi\,C_3\,L^7\,b^3\,h^5\,t^2+$
$4354560\,\pi\,C_3\,L^7\,b^4\,h^4\,t^2+3483648\,\pi\,C_3\,L^7\,b^5\,h^3\,t^2+1741824\,\pi\,C_3\,L^7\,b^6\,h^2\,t^2+34944\,C_2\,L^7\,b\,h^5\,t^4\,\pi^3+$
$34944\,C_2\,L^7\,b^5\,h\,t^4\,\pi^3+387072\,C_3\,L^7\,b\,h^6\,t^3\,\pi^2+387072\,C_3\,L^7\,b^6\,h\,t^3\,\pi^2+177408\,C_3\,L^7\,b\,h^5\,t^4\,\pi^3+$
$177408\,C_3\,L^7\,b^5\,h\,t^4\,\pi^3+161280\,\pi\,C_1\,L^7\,b^2\,h^6\,r_0\,t+322560\,\pi\,C_1\,L^7\,b^3\,h^5\,r_0\,t+403200\,\pi\,C_1\,L^7\,b^4\,h^4\,r_0\,t+$
$322560\,\pi\,C_1\,L^7\,b^5\,h^3\,r_0\,t+161280\,\pi\,C_1\,L^7\,b^6\,h^2\,r_0\,t+1483776\,\pi\,C_2\,L^7\,b^2\,h^6\,r_0\,t+$
$2967552\,\pi\,C_2\,L^7\,b^3\,h^5\,r_0\,t+3709440\,\pi\,C_2\,L^7\,b^4\,h^4\,r_0\,t+2967552\,\pi\,C_2\,L^7\,b^5\,h^3\,r_0\,t+$
$1483776\,\pi\,C_2\,L^7\,b^6\,h^2\,r_0\,t+3483648\,\pi\,C_3\,L^7\,b^2\,h^6\,r_0\,t+6967296\,\pi\,C_3\,L^7\,b^3\,h^5\,r_0\,t+$
$8709120\,\pi\,C_3\,L^7\,b^4\,h^4\,r_0\,t+6967296\,\pi\,C_3\,L^7\,b^5\,h^3\,r_0\,t+3483648\,\pi\,C_3\,L^7\,b^6\,h^2\,r_0\,t+$
$20160\,C_1\,L^7\,b^2\,h^4\,{r_0}^2\,t^2\,\pi^3+26880\,C_1\,L^7\,b^3\,h^3\,{r_0}^2\,t^2\,\pi^3+20160\,C_1\,L^7\,b^4\,h^2\,{r_0}^2\,t^2\,\pi^3+$
$524160\,C_2\,L^7\,b^2\,h^4\,{r_0}^2\,t^2\,\pi^3+698880\,C_2\,L^7\,b^3\,h^3\,{r_0}^2\,t^2\,\pi^3+524160\,C_2\,L^7\,b^4\,h^2\,{r_0}^2\,t^2\,\pi^3+$
$2661120\,C_3\,L^7\,b^2\,h^4\,{r_0}^2\,t^2\,\pi^3+3548160\,C_3\,L^7\,b^3\,h^3\,{r_0}^2\,t^2\,\pi^3+2661120\,C_3\,L^7\,b^4\,h^2\,{r_0}^2\,t^2\,\pi^3+$
$24192\,C_1\,L^7\,b\,h^6\,r_0\,t^2\,\pi^2+24192\,C_1\,L^7\,b\,h^6\,{r_0}^2\,t\,\pi^2+24192\,C_1\,L^7\,b^6\,h\,r_0\,t^2\,\pi^2+$
$24192\,C_1\,L^7\,b^6\,h\,{r_0}^2\,t\,\pi^2+5376\,C_1\,L^7\,b\,h^5\,r_0\,t^3\,\pi^3+5376\,C_1\,L^7\,b\,h^5\,{r_0}^3\,t\,\pi^3+5376\,C_1\,L^7\,b^5\,h\,r_0\,t^3\,\pi^3+$
$5376\,C_1\,L^7\,b^5\,h\,{r_0}^3\,t\,\pi^3+346752\,C_2\,L^7\,b\,h^6\,r_0\,t^2\,\pi^2+346752\,C_2\,L^7\,b\,h^6\,{r_0}^2\,t\,\pi^2+$
$346752\,C_2\,L^7\,b^6\,h\,r_0\,t^2\,\pi^2+346752\,C_2\,L^7\,b^6\,h\,{r_0}^2\,t\,\pi^2+139776\,C_2\,L^7\,b\,h^5\,r_0\,t^3\,\pi^3+$
$139776\,C_2\,L^7\,b\,h^5\,{r_0}^3\,t\,\pi^3+139776\,C_2\,L^7\,b^5\,h\,r_0\,t^3\,\pi^3+139776\,C_2\,L^7\,b^5\,h\,{r_0}^3\,t\,\pi^3+$
$1161216\,C_3\,L^7\,b\,h^6\,r_0\,t^2\,\pi^2+1161216\,C_3\,L^7\,b\,h^6\,{r_0}^2\,t\,\pi^2+1161216\,C_3\,L^7\,b^6\,h\,r_0\,t^2\,\pi^2+$
$1161216\,C_3\,L^7\,b^6\,h\,{r_0}^2\,t\,\pi^2+709632\,C_3\,L^7\,b\,h^5\,r_0\,t^3\,\pi^3+709632\,C_3\,L^7\,b\,h^5\,{r_0}^3\,t\,\pi^3+$
$709632\,C_3\,L^7\,b^5\,h\,r_0\,t^3\,\pi^3+709632\,C_3\,L^7\,b^5\,h\,{r_0}^3\,t\,\pi^3+46080\,\pi\,C_1\,L^7\,b\,h^7\,r_0\,t+$
$46080\,\pi\,C_1\,L^7\,b^7\,h\,r_0\,t+423936\,\pi\,C_2\,L^7\,b\,h^7\,r_0\,t+423936\,\pi\,C_2\,L^7\,b^7\,h\,r_0\,t+995328\,\pi\,C_3\,L^7\,b\,h^7\,r_0\,t+$
$995328\,\pi\,C_3\,L^7\,b^7\,h\,r_0\,t+72576\,C_1\,L^7\,b^2\,h^5\,r_0\,t^2\,\pi^2+72576\,C_1\,L^7\,b^2\,h^5\,{r_0}^2\,t\,\pi^2+$
$120960\,C_1\,L^7\,b^3\,h^4\,r_0\,t^2\,\pi^2+120960\,C_1\,L^7\,b^3\,h^4\,{r_0}^2\,t\,\pi^2+120960\,C_1\,L^7\,b^4\,h^3\,r_0\,t^2\,\pi^2+$
$120960\,C_1\,L^7\,b^4\,h^3\,{r_0}^2\,t\,\pi^2+72576\,C_1\,L^7\,b^5\,h^2\,r_0\,t^2\,\pi^2+72576\,C_1\,L^7\,b^5\,h^2\,{r_0}^2\,t\,\pi^2+$
$8064\,C_1\,L^7\,b\,h^5\,{r_0}^2\,t^2\,\pi^3+13440\,C_1\,L^7\,b^2\,h^4\,r_0\,t^3\,\pi^3+13440\,C_1\,L^7\,b^2\,h^4\,{r_0}^3\,t\,\pi^3+$
$17920\,C_1\,L^7\,b^3\,h^3\,r_0\,t^3\,\pi^3+17920\,C_1\,L^7\,b^3\,h^3\,{r_0}^3\,t\,\pi^3+13440\,C_1\,L^7\,b^4\,h^2\,r_0\,t^3\,\pi^3+$
$13440\,C_1\,L^7\,b^4\,h^2\,{r_0}^3\,t\,\pi^3+8064\,C_1\,L^7\,b^5\,h\,{r_0}^2\,t^2\,\pi^3+1040256\,C_2\,L^7\,b^2\,h^5\,r_0\,t^2\,\pi^2+$
$1040256\,C_2\,L^7\,b^2\,h^5\,{r_0}^2\,t\,\pi^2+1733760\,C_2\,L^7\,b^3\,h^4\,r_0\,t^2\,\pi^2+1733760\,C_2\,L^7\,b^3\,h^4\,{r_0}^2\,t\,\pi^2+$
$1733760\,C_2\,L^7\,b^4\,h^3\,r_0\,t^2\,\pi^2+1733760\,C_2\,L^7\,b^4\,h^3\,{r_0}^2\,t\,\pi^2+1040256\,C_2\,L^7\,b^5\,h^2\,r_0\,t^2\,\pi^2+$
$1040256\,C_2\,L^7\,b^5\,h^2\,{r_0}^2\,t\,\pi^2+209664\,C_2\,L^7\,b\,h^5\,{r_0}^2\,t^2\,\pi^3+349440\,C_2\,L^7\,b^2\,h^4\,r_0\,t^3\,\pi^3+$
$349440\,C_2\,L^7\,b^2\,h^4\,{r_0}^3\,t\,\pi^3+465920\,C_2\,L^7\,b^3\,h^3\,r_0\,t^3\,\pi^3+465920\,C_2\,L^7\,b^3\,h^3\,{r_0}^3\,t\,\pi^3+$
$349440\,C_2\,L^7\,b^4\,h^2\,r_0\,t^3\,\pi^3+349440\,C_2\,L^7\,b^4\,h^2\,{r_0}^3\,t\,\pi^3+209664\,C_2\,L^7\,b^5\,h\,{r_0}^2\,t^2\,\pi^3+$
$3483648\,C_3\,L^7\,b^2\,h^5\,r_0\,t^2\,\pi^2+3483648\,C_3\,L^7\,b^2\,h^5\,{r_0}^2\,t\,\pi^2+5806080\,C_3\,L^7\,b^3\,h^4\,r_0\,t^2\,\pi^2+$
$5806080\,C_3\,L^7\,b^3\,h^4\,{r_0}^2\,t\,\pi^2+5806080\,C_3\,L^7\,b^4\,h^3\,r_0\,t^2\,\pi^2+5806080\,C_3\,L^7\,b^4\,h^3\,{r_0}^2\,t\,\pi^2+$
$3483648\,C_3\,L^7\,b^5\,h^2\,r_0\,t^2\,\pi^2+3483648\,C_3\,L^7\,b^5\,h^2\,{r_0}^2\,t\,\pi^2+1064448\,C_3\,L^7\,b\,h^5\,{r_0}^2\,t^2\,\pi^3+$
$1774080\,C_3\,L^7\,b^2\,h^4\,r_0\,t^3\,\pi^3+1774080\,C_3\,L^7\,b^2\,h^4\,{r_0}^3\,t\,\pi^3+2365440\,C_3\,L^7\,b^3\,h^3\,r_0\,t^3\,\pi^3+$
$2365440\,C_3\,L^7\,b^3\,h^3\,{r_0}^3\,t\,\pi^3+1774080\,C_3\,L^7\,b^4\,h^2\,r_0\,t^3\,\pi^3+1774080\,C_3\,L^7\,b^4\,h^2\,{r_0}^3\,t\,\pi^3+$

$1064448\,C_3\,L^7\,b^5\,h\,{r_0}^2\,t^2\,\pi^3$

$\mathrm{a}_{t,6} = 5016\,\pi\,C_1\,L^8\,b^7\,t^2{+}24288\,\pi\,C_2\,L^8\,b^7\,t^2{+}25344\,\pi\,C_3\,L^8\,b^7\,t^2{+}5016\,\pi\,C_1\,L^8\,h^7\,t^2{+}$
$24288\,\pi\,C_2\,L^8\,h^7\,t^2{+}25344\,\pi\,C_3\,L^8\,h^7\,t^2{+}1852\,C_1\,L^8\,b^6\,t^3\,\pi^2{+}321\,C_1\,L^8\,b^5\,t^4\,\pi^3{+}$
$14768\,C_2\,L^8\,b^6\,t^3\,\pi^2{+}4932\,C_2\,L^8\,b^5\,t^4\,\pi^3{+}23424\,C_3\,L^8\,b^6\,t^3\,\pi^2{+}12960\,C_3\,L^8\,b^5\,t^4\,\pi^3{+}$
$1852\,C_1\,L^8\,h^6\,t^3\,\pi^2{+}321\,C_1\,L^8\,h^5\,t^4\,\pi^3{+}14768\,C_2\,L^8\,h^6\,t^3\,\pi^2{+}4932\,C_2\,L^8\,h^5\,t^4\,\pi^3{+}$
$23424\,C_3\,L^8\,h^6\,t^3\,\pi^2{+}12960\,C_3\,L^8\,h^5\,t^4\,\pi^3{+}5556\,C_1\,L^8\,b^6\,r_0\,t^2\,\pi^2{+}5556\,C_1\,L^8\,b^6\,{r_0}^2\,t\,\pi^2{+}$
$1284\,C_1\,L^8\,b^5\,r_0\,t^3\,\pi^3{+}1284\,C_1\,L^8\,b^5\,{r_0}^3\,t\,\pi^3{+}44304\,C_2\,L^8\,b^6\,r_0\,t^2\,\pi^2{+}44304\,C_2\,L^8\,b^6\,{r_0}^2\,t\,\pi^2{+}$
$19728\,C_2\,L^8\,b^5\,r_0\,t^3\,\pi^3{+}19728\,C_2\,L^8\,b^5\,{r_0}^3\,t\,\pi^3{+}70272\,C_3\,L^8\,b^6\,r_0\,t^2\,\pi^2{+}70272\,C_3\,L^8\,b^6\,{r_0}^2\,t\,\pi^2{+}$
$51840\,C_3\,L^8\,b^5\,r_0\,t^3\,\pi^3{+}51840\,C_3\,L^8\,b^5\,{r_0}^3\,t\,\pi^3{+}5556\,C_1\,L^8\,h^6\,r_0\,t^2\,\pi^2{+}5556\,C_1\,L^8\,h^6\,{r_0}^2\,t\,\pi^2{+}$
$1284\,C_1\,L^8\,h^5\,r_0\,t^3\,\pi^3{+}1284\,C_1\,L^8\,h^5\,{r_0}^3\,t\,\pi^3{+}44304\,C_2\,L^8\,h^6\,r_0\,t^2\,\pi^2{+}44304\,C_2\,L^8\,h^6\,{r_0}^2\,t\,\pi^2{+}$
$19728\,C_2\,L^8\,h^5\,r_0\,t^3\,\pi^3{+}19728\,C_2\,L^8\,h^5\,{r_0}^3\,t\,\pi^3{+}70272\,C_3\,L^8\,h^6\,r_0\,t^2\,\pi^2{+}70272\,C_3\,L^8\,h^6\,{r_0}^2\,t\,\pi^2{+}$
$51840\,C_3\,L^8\,h^5\,r_0\,t^3\,\pi^3{+}51840\,C_3\,L^8\,h^5\,{r_0}^3\,t\,\pi^3{+}10032\,\pi\,C_1\,L^8\,b^7\,r_0\,t{+}48576\,\pi\,C_2\,L^8\,b^7\,r_0\,t{+}$
$50688\,\pi\,C_3\,L^8\,b^7\,r_0\,t{+}10032\,\pi\,C_1\,L^8\,h^7\,r_0\,t{+}48576\,\pi\,C_2\,L^8\,h^7\,r_0\,t{+}50688\,\pi\,C_3\,L^8\,h^7\,r_0\,t{+}$
$27780\,C_1\,L^8\,b^2\,h^4\,t^3\,\pi^2{+}37040\,C_1\,L^8\,b^3\,h^3\,t^3\,\pi^2{+}27780\,C_1\,L^8\,b^4\,h^2\,t^3\,\pi^2{+}3210\,C_1\,L^8\,b^2\,h^3\,t^4\,\pi^3{+}$
$3210\,C_1\,L^8\,b^3\,h^2\,t^4\,\pi^3{+}221520\,C_2\,L^8\,b^2\,h^4\,t^3\,\pi^2{+}295360\,C_2\,L^8\,b^3\,h^3\,t^3\,\pi^2{+}221520\,C_2\,L^8\,b^4\,h^2\,t^3\,\pi^2{+}$
$49320\,C_2\,L^8\,b^2\,h^3\,t^4\,\pi^3{+}49320\,C_2\,L^8\,b^3\,h^2\,t^4\,\pi^3{+}351360\,C_3\,L^8\,b^2\,h^4\,t^3\,\pi^2{+}468480\,C_3\,L^8\,b^3\,h^3\,t^3\,\pi^2{+}$
$351360\,C_3\,L^8\,b^4\,h^2\,t^3\,\pi^2{+}129600\,C_3\,L^8\,b^2\,h^3\,t^4\,\pi^3{+}129600\,C_3\,L^8\,b^3\,h^2\,t^4\,\pi^3{+}1926\,C_1\,L^8\,b^5\,{r_0}^2\,t^2\,\pi^3{+}$
$29592\,C_2\,L^8\,b^5\,{r_0}^2\,t^2\,\pi^3{+}77760\,C_3\,L^8\,b^5\,{r_0}^2\,t^2\,\pi^3{+}1926\,C_1\,L^8\,h^5\,{r_0}^2\,t^2\,\pi^3{+}29592\,C_2\,L^8\,h^5\,{r_0}^2\,t^2\,\pi^3{+}$
$77760\,C_3\,L^8\,h^5\,{r_0}^2\,t^2\,\pi^3{+}35112\,\pi\,C_1\,L^8\,b\,h^6\,t^2{+}35112\,\pi\,C_1\,L^8\,b^6\,h\,t^2{+}170016\,\pi\,C_2\,L^8\,b\,h^6\,t^2{+}$
$170016\,\pi\,C_2\,L^8\,b^6\,h\,t^2{+}177408\,\pi\,C_3\,L^8\,b\,h^6\,t^2{+}177408\,\pi\,C_3\,L^8\,b^6\,h\,t^2{+}105336\,\pi\,C_1\,L^8\,b^2\,h^5\,t^2{+}$
$175560\,\pi\,C_1\,L^8\,b^3\,h^4\,t^2{+}175560\,\pi\,C_1\,L^8\,b^4\,h^3\,t^2{+}105336\,\pi\,C_1\,L^8\,b^5\,h^2\,t^2{+}11112\,C_1\,L^8\,b\,h^5\,t^3\,\pi^2{+}$
$11112\,C_1\,L^8\,b^5\,h\,t^3\,\pi^2{+}510048\,\pi\,C_2\,L^8\,b^2\,h^5\,t^2{+}850080\,\pi\,C_2\,L^8\,b^3\,h^4\,t^2{+}850080\,\pi\,C_2\,L^8\,b^4\,h^3\,t^2{+}$
$510048\,\pi\,C_2\,L^8\,b^5\,h^2\,t^2{+}1605\,C_1\,L^8\,b\,h^4\,t^4\,\pi^3{+}1605\,C_1\,L^8\,b^4\,h\,t^4\,\pi^3{+}88608\,C_2\,L^8\,b\,h^5\,t^3\,\pi^2{+}$
$88608\,C_2\,L^8\,b^5\,h\,t^3\,\pi^2{+}532224\,\pi\,C_3\,L^8\,b^2\,h^5\,t^2{+}887040\,\pi\,C_3\,L^8\,b^3\,h^4\,t^2{+}887040\,\pi\,C_3\,L^8\,b^4\,h^3\,t^2{+}$
$532224\,\pi\,C_3\,L^8\,b^5\,h^2\,t^2{+}24660\,C_2\,L^8\,b\,h^4\,t^4\,\pi^3{+}24660\,C_2\,L^8\,b^4\,h\,t^4\,\pi^3{+}140544\,C_3\,L^8\,b\,h^5\,t^3\,\pi^2{+}$
$140544\,C_3\,L^8\,b^5\,h\,t^3\,\pi^2{+}64800\,C_3\,L^8\,b\,h^4\,t^4\,\pi^3{+}64800\,C_3\,L^8\,b^4\,h\,t^4\,\pi^3{+}210672\,\pi\,C_1\,L^8\,b^2\,h^5\,r_0\,t{+}$
$351120\,\pi\,C_1\,L^8\,b^3\,h^4\,r_0\,t + 351120\,\pi\,C_1\,L^8\,b^4\,h^3\,r_0\,t + 210672\,\pi\,C_1\,L^8\,b^5\,h^2\,r_0\,t +$
$1020096\,\pi\,C_2\,L^8\,b^2\,h^5\,r_0\,t{+}1700160\,\pi\,C_2\,L^8\,b^3\,h^4\,r_0\,t{+}1700160\,\pi\,C_2\,L^8\,b^4\,h^3\,r_0\,t{+}$
$1020096\,\pi\,C_2\,L^8\,b^5\,h^2\,r_0\,t{+}1064448\,\pi\,C_3\,L^8\,b^2\,h^5\,r_0\,t{+}1774080\,\pi\,C_3\,L^8\,b^3\,h^4\,r_0\,t{+}$
$1774080\,\pi\,C_3\,L^8\,b^4\,h^3\,r_0\,t{+}1064448\,\pi\,C_3\,L^8\,b^5\,h^2\,r_0\,t{+}19260\,C_1\,L^8\,b^2\,h^3\,{r_0}^2\,t^2\,\pi^3{+}$
$19260\,C_1\,L^8\,b^3\,h^2\,{r_0}^2\,t^2\,\pi^3{+}295920\,C_2\,L^8\,b^2\,h^3\,{r_0}^2\,t^2\,\pi^3{+}295920\,C_2\,L^8\,b^3\,h^2\,{r_0}^2\,t^2\,\pi^3{+}$
$777600\,C_3\,L^8\,b^2\,h^3\,{r_0}^2\,t^2\,\pi^3{+}777600\,C_3\,L^8\,b^3\,h^2\,{r_0}^2\,t^2\,\pi^3{+}33336\,C_1\,L^8\,b\,h^5\,r_0\,t^2\,\pi^2{+}$
$33336\,C_1\,L^8\,b\,h^5\,{r_0}^2\,t\,\pi^2 + 33336\,C_1\,L^8\,b^5\,h\,r_0\,t^2\,\pi^2 + 33336\,C_1\,L^8\,b^5\,h\,{r_0}^2\,t\,\pi^2 +$
$6420\,C_1\,L^8\,b\,h^4\,r_0\,t^3\,\pi^3{+}6420\,C_1\,L^8\,b\,h^4\,{r_0}^3\,t\,\pi^3{+}6420\,C_1\,L^8\,b^4\,h\,r_0\,t^3\,\pi^3{+}6420\,C_1\,L^8\,b^4\,h\,{r_0}^3\,t\,\pi^3{+}$
$265824\,C_2\,L^8\,b\,h^5\,r_0\,t^2\,\pi^2{+}265824\,C_2\,L^8\,b\,h^5\,{r_0}^2\,t\,\pi^2{+}265824\,C_2\,L^8\,b^5\,h\,r_0\,t^2\,\pi^2{+}$
$265824\,C_2\,L^8\,b^5\,h\,{r_0}^2\,t\,\pi^2 + 98640\,C_2\,L^8\,b\,h^4\,r_0\,t^3\,\pi^3 + 98640\,C_2\,L^8\,b\,h^4\,{r_0}^3\,t\,\pi^3 +$
$98640\,C_2\,L^8\,b^4\,h\,r_0\,t^3\,\pi^3 + 98640\,C_2\,L^8\,b^4\,h\,{r_0}^3\,t\,\pi^3 + 421632\,C_3\,L^8\,b\,h^5\,r_0\,t^2\,\pi^2 +$
$421632\,C_3\,L^8\,b\,h^5\,{r_0}^2\,t\,\pi^2{+}421632\,C_3\,L^8\,b^5\,h\,r_0\,t^2\,\pi^2{+}421632\,C_3\,L^8\,b^5\,h\,{r_0}^2\,t\,\pi^2{+}$
$259200\,C_3\,L^8\,b\,h^4\,r_0\,t^3\,\pi^3{+}259200\,C_3\,L^8\,b\,h^4\,{r_0}^3\,t\,\pi^3{+}259200\,C_3\,L^8\,b^4\,h\,r_0\,t^3\,\pi^3{+}$
$259200\,C_3\,L^8\,b^4\,h\,{r_0}^3\,t\,\pi^3{+}70224\,\pi\,C_1\,L^8\,b\,h^6\,r_0\,t{+}70224\,\pi\,C_1\,L^8\,b^6\,h\,r_0\,t{+}340032\,\pi\,C_2\,L^8\,b\,h^6\,r_0\,t{+}$
$340032\,\pi\,C_2\,L^8\,b^6\,h\,r_0\,t{+}354816\,\pi\,C_3\,L^8\,b\,h^6\,r_0\,t{+}354816\,\pi\,C_3\,L^8\,b^6\,h\,r_0\,t{+}83340\,C_1\,L^8\,b^2\,h^4\,r_0\,t^2\,\pi^2{+}$
$83340\,C_1\,L^8\,b^2\,h^4\,{r_0}^2\,t\,\pi^2{+}111120\,C_1\,L^8\,b^3\,h^3\,r_0\,t^2\,\pi^2{+}111120\,C_1\,L^8\,b^3\,h^3\,{r_0}^2\,t\,\pi^2{+}$
$83340\,C_1\,L^8\,b^4\,h^2\,r_0\,t^2\,\pi^2 + 83340\,C_1\,L^8\,b^4\,h^2\,{r_0}^2\,t\,\pi^2 + 9630\,C_1\,L^8\,b\,h^4\,{r_0}^2\,t^2\,\pi^3 +$
$12840\,C_1\,L^8\,b^2\,h^3\,r_0\,t^3\,\pi^3{+}12840\,C_1\,L^8\,b^2\,h^3\,{r_0}^3\,t\,\pi^3{+}12840\,C_1\,L^8\,b^3\,h^2\,r_0\,t^3\,\pi^3{+}$
$12840\,C_1\,L^8\,b^3\,h^2\,{r_0}^3\,t\,\pi^3{+}9630\,C_1\,L^8\,b^4\,h\,{r_0}^2\,t^2\,\pi^3{+}664560\,C_2\,L^8\,b^2\,h^4\,r_0\,t^2\,\pi^2{+}$

$664560\,C_2\,L^8\,b^2\,h^4\,{r_0}^2\,t\,\pi^2+886080\,C_2\,L^8\,b^3\,h^3\,r_0\,t^2\,\pi^2+886080\,C_2\,L^8\,b^3\,h^3\,{r_0}^2\,t\,\pi^2+$
$664560\,C_2\,L^8\,b^4\,h^2\,r_0\,t^2\,\pi^2+664560\,C_2\,L^8\,b^4\,h^2\,{r_0}^2\,t\,\pi^2+147960\,C_2\,L^8\,b\,h^4\,{r_0}^2\,t^2\,\pi^3+$
$197280\,C_2\,L^8\,b^2\,h^3\,r_0\,t^3\,\pi^3+197280\,C_2\,L^8\,b^2\,h^3\,{r_0}^3\,t\,\pi^3+197280\,C_2\,L^8\,b^3\,h^2\,r_0\,t^3\,\pi^3+$
$197280\,C_2\,L^8\,b^3\,h^2\,{r_0}^3\,t\,\pi^3+147960\,C_2\,L^8\,b^4\,h\,{r_0}^2\,t^2\,\pi^3+1054080\,C_3\,L^8\,b^2\,h^4\,r_0\,t^2\,\pi^2+$
$1054080\,C_3\,L^8\,b^2\,h^4\,{r_0}^2\,t\,\pi^2+1405440\,C_3\,L^8\,b^3\,h^3\,r_0\,t^2\,\pi^2+1405440\,C_3\,L^8\,b^3\,h^3\,{r_0}^2\,t\,\pi^2+$
$1054080\,C_3\,L^8\,b^4\,h^2\,r_0\,t^2\,\pi^2+1054080\,C_3\,L^8\,b^4\,h^2\,{r_0}^2\,t\,\pi^2+388800\,C_3\,L^8\,b\,h^4\,{r_0}^2\,t^2\,\pi^3+$
$518400\,C_3\,L^8\,b^2\,h^3\,r_0\,t^3\,\pi^3+518400\,C_3\,L^8\,b^2\,h^3\,{r_0}^3\,t\,\pi^3+518400\,C_3\,L^8\,b^3\,h^2\,r_0\,t^3\,\pi^3+$
$518400\,C_3\,L^8\,b^3\,h^2\,{r_0}^3\,t\,\pi^3+388800\,C_3\,L^8\,b^4\,h\,{r_0}^2\,t^2\,\pi^3$

$\mathrm{a}_{t,5}=5904\,\pi\,C_1\,L^9\,b^6\,t^2+14400\,\pi\,C_2\,L^9\,b^6\,t^2+4608\,\pi\,C_3\,L^9\,b^6\,t^2+5904\,\pi\,C_1\,L^9\,h^6\,t^2+$
$14400\,\pi\,C_2\,L^9\,h^6\,t^2+4608\,\pi\,C_3\,L^9\,h^6\,t^2+2032\,C_1\,L^9\,b^5\,t^3\,\pi^2+306\,C_1\,L^9\,b^4\,t^4\,\pi^3+$
$8768\,C_2\,L^9\,b^5\,t^3\,\pi^2+2856\,C_2\,L^9\,b^4\,t^4\,\pi^3+4608\,C_3\,L^9\,b^5\,t^3\,\pi^2+2880\,C_3\,L^9\,b^4\,t^4\,\pi^3+$
$2032\,C_1\,L^9\,h^5\,t^3\,\pi^2+306\,C_1\,L^9\,h^4\,t^4\,\pi^3+8768\,C_2\,L^9\,h^5\,t^3\,\pi^2+2856\,C_2\,L^9\,h^4\,t^4\,\pi^3+$
$4608\,C_3\,L^9\,h^5\,t^3\,\pi^2+2880\,C_3\,L^9\,h^4\,t^4\,\pi^3+6096\,C_1\,L^9\,b^5\,r_0\,t^2\,\pi^2+6096\,C_1\,L^9\,b^5\,{r_0}^2\,t\,\pi^2+$
$1224\,C_1\,L^9\,b^4\,r_0\,t^3\,\pi^3+1224\,C_1\,L^9\,b^4\,{r_0}^3\,t\,\pi^3+26304\,C_2\,L^9\,b^5\,r_0\,t^2\,\pi^2+26304\,C_2\,L^9\,b^5\,{r_0}^2\,t\,\pi^2+$
$11424\,C_2\,L^9\,b^4\,r_0\,t^3\,\pi^3+11424\,C_2\,L^9\,b^4\,{r_0}^3\,t\,\pi^3+13824\,C_3\,L^9\,b^5\,r_0\,t^2\,\pi^2+13824\,C_3\,L^9\,b^5\,{r_0}^2\,t\,\pi^2+$
$11520\,C_3\,L^9\,b^4\,r_0\,t^3\,\pi^3+11520\,C_3\,L^9\,b^4\,{r_0}^3\,t\,\pi^3+6096\,C_1\,L^9\,h^5\,r_0\,t^2\,\pi^2+6096\,C_1\,L^9\,h^5\,{r_0}^2\,t\,\pi^2+$
$1224\,C_1\,L^9\,h^4\,r_0\,t^3\,\pi^3+1224\,C_1\,L^9\,h^4\,{r_0}^3\,t\,\pi^3+26304\,C_2\,L^9\,h^5\,r_0\,t^2\,\pi^2+26304\,C_2\,L^9\,h^5\,{r_0}^2\,t\,\pi^2+$
$11424\,C_2\,L^9\,h^4\,r_0\,t^3\,\pi^3+11424\,C_2\,L^9\,h^4\,{r_0}^3\,t\,\pi^3+13824\,C_3\,L^9\,h^5\,r_0\,t^2\,\pi^2+13824\,C_3\,L^9\,h^5\,{r_0}^2\,t\,\pi^2+$
$11520\,C_3\,L^9\,h^4\,r_0\,t^3\,\pi^3+11520\,C_3\,L^9\,h^4\,{r_0}^3\,t\,\pi^3+11808\,\pi\,C_1\,L^9\,b^6\,r_0\,t+28800\,\pi\,C_2\,L^9\,b^6\,r_0\,t+$
$9216\,\pi\,C_3\,L^9\,b^6\,r_0\,t+11808\,\pi\,C_1\,L^9\,h^6\,r_0\,t+28800\,\pi\,C_2\,L^9\,h^6\,r_0\,t+9216\,\pi\,C_3\,L^9\,h^6\,r_0\,t+$
$20320\,C_1\,L^9\,b^2\,h^3\,t^3\,\pi^2+20320\,C_1\,L^9\,b^3\,h^2\,t^3\,\pi^2+1836\,C_1\,L^9\,b^2\,h^2\,t^4\,\pi^3+87680\,C_2\,L^9\,b^2\,h^3\,t^3\,\pi^2+$
$87680\,C_2\,L^9\,b^3\,h^2\,t^3\,\pi^2+17136\,C_2\,L^9\,b^2\,h^2\,t^4\,\pi^3+46080\,C_3\,L^9\,b^2\,h^3\,t^3\,\pi^2+46080\,C_3\,L^9\,b^3\,h^2\,t^3\,\pi^2+$
$17280\,C_3\,L^9\,b^2\,h^2\,t^4\,\pi^3+1836\,C_1\,L^9\,b^4\,{r_0}^2\,t^2\,\pi^3+17136\,C_2\,L^9\,b^4\,{r_0}^2\,t^2\,\pi^3+17280\,C_3\,L^9\,b^4\,{r_0}^2\,t^2\,\pi^3+$
$1836\,C_1\,L^9\,h^4\,{r_0}^2\,t^2\,\pi^3+17136\,C_2\,L^9\,h^4\,{r_0}^2\,t^2\,\pi^3+17280\,C_3\,L^9\,h^4\,{r_0}^2\,t^2\,\pi^3+35424\,\pi\,C_1\,L^9\,b\,h^5\,t^2+$
$35424\,\pi\,C_1\,L^9\,b^5\,h\,t^2+86400\,\pi\,C_2\,L^9\,b\,h^5\,t^2+86400\,\pi\,C_2\,L^9\,b^5\,h\,t^2+27648\,\pi\,C_3\,L^9\,b\,h^5\,t^2+$
$27648\,\pi\,C_3\,L^9\,b^5\,h\,t^2+88560\,\pi\,C_1\,L^9\,b^2\,h^4\,t^2+118080\,\pi\,C_1\,L^9\,b^3\,h^3\,t^2+88560\,\pi\,C_1\,L^9\,b^4\,h^2\,t^2+$
$10160\,C_1\,L^9\,b\,h^4\,t^3\,\pi^2+10160\,C_1\,L^9\,b^4\,h\,t^3\,\pi^2+216000\,\pi\,C_2\,L^9\,b^2\,h^4\,t^2+288000\,\pi\,C_2\,L^9\,b^3\,h^3\,t^2+$
$216000\,\pi\,C_2\,L^9\,b^4\,h^2\,t^2+1224\,C_1\,L^9\,b\,h^3\,t^4\,\pi^3+1224\,C_1\,L^9\,b^3\,h\,t^4\,\pi^3+43840\,C_2\,L^9\,b\,h^4\,t^3\,\pi^2+$
$43840\,C_2\,L^9\,b^4\,h\,t^3\,\pi^2+69120\,\pi\,C_3\,L^9\,b^2\,h^4\,t^2+92160\,\pi\,C_3\,L^9\,b^3\,h^3\,t^2+69120\,\pi\,C_3\,L^9\,b^4\,h^2\,t^2+$
$11424\,C_2\,L^9\,b\,h^3\,t^4\,\pi^3+11424\,C_2\,L^9\,b^3\,h\,t^4\,\pi^3+23040\,C_3\,L^9\,b\,h^4\,t^3\,\pi^2+23040\,C_3\,L^9\,b^4\,h\,t^3\,\pi^2+$
$11520\,C_3\,L^9\,b\,h^3\,t^4\,\pi^3+11520\,C_3\,L^9\,b^3\,h\,t^4\,\pi^3+177120\,\pi\,C_1\,L^9\,b^2\,h^4\,r_0\,t+236160\,\pi\,C_1\,L^9\,b^3\,h^3\,r_0\,t+$
$177120\,\pi\,C_1\,L^9\,b^4\,h^2\,r_0\,t+432000\,\pi\,C_2\,L^9\,b^2\,h^4\,r_0\,t+576000\,\pi\,C_2\,L^9\,b^3\,h^3\,r_0\,t+$
$432000\,\pi\,C_2\,L^9\,b^4\,h^2\,r_0\,t+138240\,\pi\,C_3\,L^9\,b^2\,h^4\,r_0\,t+184320\,\pi\,C_3\,L^9\,b^3\,h^3\,r_0\,t+$
$138240\,\pi\,C_3\,L^9\,b^4\,h^2\,r_0\,t+11016\,C_1\,L^9\,b^2\,h^2\,{r_0}^2\,t^2\,\pi^3+102816\,C_2\,L^9\,b^2\,h^2\,{r_0}^2\,t^2\,\pi^3+$
$103680\,C_3\,L^9\,b^2\,h^2\,{r_0}^2\,t^2\,\pi^3+30480\,C_1\,L^9\,b\,h^4\,r_0\,t^2\,\pi^2+30480\,C_1\,L^9\,b\,h^4\,{r_0}^2\,t\,\pi^2+$
$30480\,C_1\,L^9\,b^4\,h\,r_0\,t^2\,\pi^2+30480\,C_1\,L^9\,b^4\,h\,{r_0}^2\,t\,\pi^2+4896\,C_1\,L^9\,b\,h^3\,r_0\,t^3\,\pi^3+4896\,C_1\,L^9\,b\,h^3\,{r_0}^3\,t\,\pi^3+$
$4896\,C_1\,L^9\,b^3\,h\,r_0\,t^3\,\pi^3+4896\,C_1\,L^9\,b^3\,h\,{r_0}^3\,t\,\pi^3+131520\,C_2\,L^9\,b\,h^4\,r_0\,t^2\,\pi^2+131520\,C_2\,L^9\,b\,h^4\,{r_0}^2\,t\,\pi^2+$
$131520\,C_2\,L^9\,b^4\,h\,r_0\,t^2\,\pi^2+131520\,C_2\,L^9\,b^4\,h\,{r_0}^2\,t\,\pi^2+45696\,C_2\,L^9\,b\,h^3\,r_0\,t^3\,\pi^3+$
$45696\,C_2\,L^9\,b\,h^3\,{r_0}^3\,t\,\pi^3+45696\,C_2\,L^9\,b^3\,h\,r_0\,t^3\,\pi^3+45696\,C_2\,L^9\,b^3\,h\,{r_0}^3\,t\,\pi^3+$
$69120\,C_3\,L^9\,b\,h^4\,r_0\,t^2\,\pi^2+69120\,C_3\,L^9\,b\,h^4\,{r_0}^2\,t\,\pi^2+69120\,C_3\,L^9\,b^4\,h\,r_0\,t^2\,\pi^2+$
$69120\,C_3\,L^9\,b^4\,h\,{r_0}^2\,t\,\pi^2+46080\,C_3\,L^9\,b\,h^3\,r_0\,t^3\,\pi^3+46080\,C_3\,L^9\,b\,h^3\,{r_0}^3\,t\,\pi^3+$
$46080\,C_3\,L^9\,b^3\,h\,r_0\,t^3\,\pi^3+46080\,C_3\,L^9\,b^3\,h\,{r_0}^3\,t\,\pi^3+70848\,\pi\,C_1\,L^9\,b\,h^5\,r_0\,t+70848\,\pi\,C_1\,L^9\,b^5\,h\,r_0\,t+$
$172800\,\pi\,C_2\,L^9\,b\,h^5\,r_0\,t+172800\,\pi\,C_2\,L^9\,b^5\,h\,r_0\,t+55296\,\pi\,C_3\,L^9\,b\,h^5\,r_0\,t+55296\,\pi\,C_3\,L^9\,b^5\,h\,r_0\,t+$
$60960\,C_1\,L^9\,b^2\,h^3\,r_0\,t^2\,\pi^2+60960\,C_1\,L^9\,b^2\,h^3\,{r_0}^2\,t\,\pi^2+60960\,C_1\,L^9\,b^3\,h^2\,r_0\,t^2\,\pi^2+$
$60960\,C_1\,L^9\,b^3\,h^2\,{r_0}^2\,t\,\pi^2+7344\,C_1\,L^9\,b\,h^3\,{r_0}^2\,t^2\,\pi^3+7344\,C_1\,L^9\,b^2\,h^2\,r_0\,t^3\,\pi^3+$

$7344\, C_1\, L^9\, b^2\, h^2\, {r_0}^3\, t\, \pi^3 + 7344\, C_1\, L^9\, b^3\, h\, {r_0}^2\, t^2\, \pi^3 + 263040\, C_2\, L^9\, b^2\, h^3\, r_0\, t^2\, \pi^2 +$
$263040\, C_2\, L^9\, b^2\, h^3\, {r_0}^2\, t\, \pi^2 + 263040\, C_2\, L^9\, b^3\, h^2\, r_0\, t^2\, \pi^2 + 263040\, C_2\, L^9\, b^3\, h^2\, {r_0}^2\, t\, \pi^2 +$
$68544\, C_2\, L^9\, b\, h^3\, {r_0}^2\, t^2\, \pi^3 + 68544\, C_2\, L^9\, b^2\, h^2\, r_0\, t^3\, \pi^3 + 68544\, C_2\, L^9\, b^2\, h^2\, {r_0}^3\, t\, \pi^3 +$
$68544\, C_2\, L^9\, b^3\, h\, {r_0}^2\, t^2\, \pi^3 + 138240\, C_3\, L^9\, b^2\, h^3\, r_0\, t^2\, \pi^2 + 138240\, C_3\, L^9\, b^2\, h^3\, {r_0}^2\, t\, \pi^2 +$
$138240\, C_3\, L^9\, b^3\, h^2\, r_0\, t^2\, \pi^2 + 138240\, C_3\, L^9\, b^3\, h^2\, {r_0}^2\, t\, \pi^2 + 69120\, C_3\, L^9\, b\, h^3\, {r_0}^2\, t^2\, \pi^3 +$
$69120\, C_3\, L^9\, b^2\, h^2\, r_0\, t^3\, \pi^3 + 69120\, C_3\, L^9\, b^2\, h^2\, {r_0}^3\, t\, \pi^3 + 69120\, C_3\, L^9\, b^3\, h\, {r_0}^2\, t^2\, \pi^3$

$\mathrm{a}_{t,4} = 4680\, \pi\, C_1\, L^{10}\, b^5\, t^2 + 4992\, \pi\, C_2\, L^{10}\, b^5\, t^2 + 4680\, \pi\, C_1\, L^{10}\, h^5\, t^2 + 4992\, \pi\, C_2\, L^{10}\, h^5\, t^2 +$
$1532\, C_1\, L^{10}\, b^4\, t^3\, \pi^2 + 198\, C_1\, L^{10}\, b^3\, t^4\, \pi^3 + 3136\, C_2\, L^{10}\, b^4\, t^3\, \pi^2 + 1056\, C_2\, L^{10}\, b^3\, t^4\, \pi^3 +$
$1532\, C_1\, L^{10}\, h^4\, t^3\, \pi^2 + 198\, C_1\, L^{10}\, h^3\, t^4\, \pi^3 + 3136\, C_2\, L^{10}\, h^4\, t^3\, \pi^2 + 1056\, C_2\, L^{10}\, h^3\, t^4\, \pi^3 +$
$4596\, C_1\, L^{10}\, b^4\, r_0\, t^2\, \pi^2 + 4596\, C_1\, L^{10}\, b^4\, {r_0}^2\, t\, \pi^2 + 792\, C_1\, L^{10}\, b^3\, r_0\, t^3\, \pi^3 + 792\, C_1\, L^{10}\, b^3\, {r_0}^3\, t\, \pi^3 +$
$9408\, C_2\, L^{10}\, b^4\, r_0\, t^2\, \pi^2 + 9408\, C_2\, L^{10}\, b^4\, {r_0}^2\, t\, \pi^2 + 4224\, C_2\, L^{10}\, b^3\, r_0\, t^3\, \pi^3 + 4224\, C_2\, L^{10}\, b^3\, {r_0}^3\, t\, \pi^3 +$
$4596\, C_1\, L^{10}\, h^4\, r_0\, t^2\, \pi^2 + 4596\, C_1\, L^{10}\, h^4\, {r_0}^2\, t\, \pi^2 + 792\, C_1\, L^{10}\, h^3\, r_0\, t^3\, \pi^3 + 792\, C_1\, L^{10}\, h^3\, {r_0}^3\, t\, \pi^3 +$
$9408\, C_2\, L^{10}\, h^4\, r_0\, t^2\, \pi^2 + 9408\, C_2\, L^{10}\, h^4\, {r_0}^2\, t\, \pi^2 + 4224\, C_2\, L^{10}\, h^3\, r_0\, t^3\, \pi^3 + 4224\, C_2\, L^{10}\, h^3\, {r_0}^3\, t\, \pi^3 +$
$9360\, \pi\, C_1\, L^{10}\, b^5\, r_0\, t + 9984\, \pi\, C_2\, L^{10}\, b^5\, r_0\, t + 9360\, \pi\, C_1\, L^{10}\, h^5\, r_0\, t + 9984\, \pi\, C_2\, L^{10}\, h^5\, r_0\, t +$
$9192\, C_1\, L^{10}\, b^2\, h^2\, t^3\, \pi^2 + 18816\, C_2\, L^{10}\, b^2\, h^2\, t^3\, \pi^2 + 1188\, C_1\, L^{10}\, b^3\, {r_0}^2\, t^2\, \pi^3 + 6336\, C_2\, L^{10}\, b^3\, {r_0}^2\, t^2\, \pi^3 +$
$1188\, C_1\, L^{10}\, h^3\, {r_0}^2\, t^2\, \pi^3 + 6336\, C_2\, L^{10}\, h^3\, {r_0}^2\, t^2\, \pi^3 + 23400\, \pi\, C_1\, L^{10}\, b\, h^4\, t^2 + 23400\, \pi\, C_1\, L^{10}\, b^4\, h\, t^2 +$
$24960\, \pi\, C_2\, L^{10}\, b\, h^4\, t^2 + 24960\, \pi\, C_2\, L^{10}\, b^4\, h\, t^2 + 46800\, \pi\, C_1\, L^{10}\, b^2\, h^3\, t^2 + 46800\, \pi\, C_1\, L^{10}\, b^3\, h^2\, t^2 +$
$6128\, C_1\, L^{10}\, b\, h^3\, t^3\, \pi^2 + 6128\, C_1\, L^{10}\, b^3\, h\, t^3\, \pi^2 + 49920\, \pi\, C_2\, L^{10}\, b^2\, h^3\, t^2 + 49920\, \pi\, C_2\, L^{10}\, b^3\, h^2\, t^2 +$
$594\, C_1\, L^{10}\, b\, h^2\, t^4\, \pi^3 + 594\, C_1\, L^{10}\, b^2\, h\, t^4\, \pi^3 + 12544\, C_2\, L^{10}\, b\, h^3\, t^3\, \pi^2 + 12544\, C_2\, L^{10}\, b^3\, h\, t^3\, \pi^2 +$
$3168\, C_2\, L^{10}\, b\, h^2\, t^4\, \pi^3 + 3168\, C_2\, L^{10}\, b^2\, h\, t^4\, \pi^3 + 93600\, \pi\, C_1\, L^{10}\, b^2\, h^3\, r_0\, t + 93600\, \pi\, C_1\, L^{10}\, b^3\, h^2\, r_0\, t +$
$99840\, \pi\, C_2\, L^{10}\, b^2\, h^3\, r_0\, t + 99840\, \pi\, C_2\, L^{10}\, b^3\, h^2\, r_0\, t + 18384\, C_1\, L^{10}\, b\, h^3\, r_0\, t^2\, \pi^2 +$
$18384\, C_1\, L^{10}\, b\, h^3\, {r_0}^2\, t\, \pi^2 + 18384\, C_1\, L^{10}\, b^3\, h\, r_0\, t^2\, \pi^2 + 18384\, C_1\, L^{10}\, b^3\, h\, {r_0}^2\, t\, \pi^2 +$
$2376\, C_1\, L^{10}\, b\, h^2\, r_0\, t^3\, \pi^3 + 2376\, C_1\, L^{10}\, b\, h^2\, {r_0}^3\, t\, \pi^3 + 2376\, C_1\, L^{10}\, b^2\, h\, r_0\, t^3\, \pi^3 + 2376\, C_1\, L^{10}\, b^2\, h\, {r_0}^3\, t\, \pi^3 +$
$37632\, C_2\, L^{10}\, b\, h^3\, r_0\, t^2\, \pi^2 + 37632\, C_2\, L^{10}\, b\, h^3\, {r_0}^2\, t\, \pi^2 + 37632\, C_2\, L^{10}\, b^3\, h\, r_0\, t^2\, \pi^2 +$
$37632\, C_2\, L^{10}\, b^3\, h\, {r_0}^2\, t\, \pi^2 + 12672\, C_2\, L^{10}\, b\, h^2\, r_0\, t^3\, \pi^3 + 12672\, C_2\, L^{10}\, b\, h^2\, {r_0}^3\, t\, \pi^3 +$
$12672\, C_2\, L^{10}\, b^2\, h\, r_0\, t^3\, \pi^3 + 12672\, C_2\, L^{10}\, b^2\, h\, {r_0}^3\, t\, \pi^3 + 46800\, \pi\, C_1\, L^{10}\, b\, h^4\, r_0\, t +$
$46800\, \pi\, C_1\, L^{10}\, b^4\, h\, r_0\, t + 49920\, \pi\, C_2\, L^{10}\, b\, h^4\, r_0\, t + 49920\, \pi\, C_2\, L^{10}\, b^4\, h\, r_0\, t + 27576\, C_1\, L^{10}\, b^2\, h^2\, r_0\, t^2\, \pi^2 +$
$27576\, C_1\, L^{10}\, b^2\, h^2\, {r_0}^2\, t\, \pi^2 + 3564\, C_1\, L^{10}\, b\, h^2\, {r_0}^2\, t^2\, \pi^3 + 3564\, C_1\, L^{10}\, b^2\, h\, {r_0}^2\, t^2\, \pi^3 +$
$56448\, C_2\, L^{10}\, b^2\, h^2\, r_0\, t^2\, \pi^2 + 56448\, C_2\, L^{10}\, b^2\, h^2\, {r_0}^2\, t\, \pi^2 + 19008\, C_2\, L^{10}\, b\, h^2\, {r_0}^2\, t^2\, \pi^3 +$
$19008\, C_2\, L^{10}\, b^2\, h\, {r_0}^2\, t^2\, \pi^3$

$\mathrm{a}_{t,3} = 2400\, \pi\, C_1\, L^{11}\, b^4\, t^2 + 768\, \pi\, C_2\, L^{11}\, b^4\, t^2 + 2400\, \pi\, C_1\, L^{11}\, h^4\, t^2 + 768\, \pi\, C_2\, L^{11}\, h^4\, t^2 +$
$768\, C_1\, L^{11}\, b^3\, t^3\, \pi^2 + 88\, C_1\, L^{11}\, b^2\, t^4\, \pi^3 + 512\, C_2\, L^{11}\, b^3\, t^3\, \pi^2 + 192\, C_2\, L^{11}\, b^2\, t^4\, \pi^3 +$
$768\, C_1\, L^{11}\, h^3\, t^3\, \pi^2 + 88\, C_1\, L^{11}\, h^2\, t^4\, \pi^3 + 512\, C_2\, L^{11}\, h^3\, t^3\, \pi^2 + 192\, C_2\, L^{11}\, h^2\, t^4\, \pi^3 +$
$2304\, C_1\, L^{11}\, b^3\, r_0\, t^2\, \pi^2 + 2304\, C_1\, L^{11}\, b^3\, {r_0}^2\, t\, \pi^2 + 352\, C_1\, L^{11}\, b^2\, r_0\, t^3\, \pi^3 + 352\, C_1\, L^{11}\, b^2\, {r_0}^3\, t\, \pi^3 +$
$1536\, C_2\, L^{11}\, b^3\, r_0\, t^2\, \pi^2 + 1536\, C_2\, L^{11}\, b^3\, {r_0}^2\, t\, \pi^2 + 768\, C_2\, L^{11}\, b^2\, r_0\, t^3\, \pi^3 + 768\, C_2\, L^{11}\, b^2\, {r_0}^3\, t\, \pi^3 +$
$2304\, C_1\, L^{11}\, h^3\, r_0\, t^2\, \pi^2 + 2304\, C_1\, L^{11}\, h^3\, {r_0}^2\, t\, \pi^2 + 352\, C_1\, L^{11}\, h^2\, r_0\, t^3\, \pi^3 + 352\, C_1\, L^{11}\, h^2\, {r_0}^3\, t\, \pi^3 +$
$1536\, C_2\, L^{11}\, h^3\, r_0\, t^2\, \pi^2 + 1536\, C_2\, L^{11}\, h^3\, {r_0}^2\, t\, \pi^2 + 768\, C_2\, L^{11}\, h^2\, r_0\, t^3\, \pi^3 + 768\, C_2\, L^{11}\, h^2\, {r_0}^3\, t\, \pi^3 +$
$4800\, \pi\, C_1\, L^{11}\, b^4\, r_0\, t + 1536\, \pi\, C_2\, L^{11}\, b^4\, r_0\, t + 4800\, \pi\, C_1\, L^{11}\, h^4\, r_0\, t + 1536\, \pi\, C_2\, L^{11}\, h^4\, r_0\, t +$
$528\, C_1\, L^{11}\, b^2\, {r_0}^2\, t^2\, \pi^3 + 1152\, C_2\, L^{11}\, b^2\, {r_0}^2\, t^2\, \pi^3 + 528\, C_1\, L^{11}\, h^2\, {r_0}^2\, t^2\, \pi^3 + 1152\, C_2\, L^{11}\, h^2\, {r_0}^2\, t^2\, \pi^3 +$
$9600\, \pi\, C_1\, L^{11}\, b\, h^3\, t^2 + 9600\, \pi\, C_1\, L^{11}\, b^3\, h\, t^2 + 3072\, \pi\, C_2\, L^{11}\, b\, h^3\, t^2 + 3072\, \pi\, C_2\, L^{11}\, b^3\, h\, t^2 +$
$176\, C_1\, L^{11}\, b\, h\, t^4\, \pi^3 + 384\, C_2\, L^{11}\, b\, h\, t^4\, \pi^3 + 14400\, \pi\, C_1\, L^{11}\, b^2\, h^2\, t^2 + 2304\, C_1\, L^{11}\, b\, h^2\, t^3\, \pi^2 +$
$2304\, C_1\, L^{11}\, b^2\, h\, t^3\, \pi^2 + 4608\, \pi\, C_2\, L^{11}\, b^2\, h^2\, t^2 + 1536\, C_2\, L^{11}\, b\, h^2\, t^3\, \pi^2 + 1536\, C_2\, L^{11}\, b^2\, h\, t^3\, \pi^2 +$
$28800\, \pi\, C_1\, L^{11}\, b^2\, h^2\, r_0\, t + 9216\, \pi\, C_2\, L^{11}\, b^2\, h^2\, r_0\, t + 704\, C_1\, L^{11}\, b\, h\, r_0\, t^3\, \pi^3 + 704\, C_1\, L^{11}\, b\, h\, {r_0}^3\, t\, \pi^3 +$
$1536\, C_2\, L^{11}\, b\, h\, r_0\, t^3\, \pi^3 + 1536\, C_2\, L^{11}\, b\, h\, {r_0}^3\, t\, \pi^3 + 6912\, C_1\, L^{11}\, b\, h^2\, r_0\, t^2\, \pi^2 + 6912\, C_1\, L^{11}\, b\, h^2\, {r_0}^2\, t\, \pi^2 +$

$6912\,C_1\,L^{11}\,b^2\,h\,r_0\,t^2\,\pi^2+6912\,C_1\,L^{11}\,b^2\,h\,{r_0}^2\,t\,\pi^2+1056\,C_1\,L^{11}\,b\,h\,{r_0}^2\,t^2\,\pi^3+4608\,C_2\,L^{11}\,b\,h^2\,r_0\,t^2\,\pi^2+$
$4608\,C_2\,L^{11}\,b\,h^2\,{r_0}^2\,t\,\pi^2+4608\,C_2\,L^{11}\,b^2\,h\,r_0\,t^2\,\pi^2+4608\,C_2\,L^{11}\,b^2\,h\,{r_0}^2\,t\,\pi^2+2304\,C_2\,L^{11}\,b\,h\,{r_0}^2\,t^2\,\pi^3+$
$19200\,\pi\,C_1\,L^{11}\,b\,h^3\,r_0\,t+19200\,\pi\,C_1\,L^{11}\,b^3\,h\,r_0\,t+6144\,\pi\,C_2\,L^{11}\,b\,h^3\,r_0\,t+6144\,\pi\,C_2\,L^{11}\,b^3\,h\,r_0\,t$

$\mathrm{a}_{t,2} = 1440\,C_1\,\pi\,L^{12}\,b^3\,r_0\,t+720\,C_1\,\pi\,L^{12}\,b^3\,t^2+4320\,C_1\,\pi\,L^{12}\,b^2\,h\,r_0\,t+2160\,C_1\,\pi\,L^{12}\,b^2\,h\,t^2+$
$696\,C_1\,\pi^2\,L^{12}\,b^2\,{r_0}^2\,t+696\,C_1\,\pi^2\,L^{12}\,b^2\,r_0\,t^2+232\,C_1\,\pi^2\,L^{12}\,b^2\,t^3+4320\,C_1\,\pi\,L^{12}\,b\,h^2\,r_0\,t+$
$2160\,C_1\,\pi\,L^{12}\,b\,h^2\,t^2+1392\,C_1\,\pi^2\,L^{12}\,b\,h\,{r_0}^2\,t+1392\,C_1\,\pi^2\,L^{12}\,b\,h\,r_0\,t^2+464\,C_1\,\pi^2\,L^{12}\,b\,h\,t^3+$
$104\,C_1\,\pi^3\,L^{12}\,b\,{r_0}^3\,t+156\,C_1\,\pi^3\,L^{12}\,b\,{r_0}^2\,t^2+104\,C_1\,\pi^3\,L^{12}\,b\,r_0\,t^3+26\,C_1\,\pi^3\,L^{12}\,b\,t^4+$
$1440\,C_1\,\pi\,L^{12}\,h^3\,r_0\,t+720\,C_1\,\pi\,L^{12}\,h^3\,t^2+696\,C_1\,\pi^2\,L^{12}\,h^2\,{r_0}^2\,t+696\,C_1\,\pi^2\,L^{12}\,h^2\,r_0\,t^2+$
$232\,C_1\,\pi^2\,L^{12}\,h^2\,t^3+104\,C_1\,\pi^3\,L^{12}\,h\,{r_0}^3\,t+156\,C_1\,\pi^3\,L^{12}\,h\,{r_0}^2\,t^2+104\,C_1\,\pi^3\,L^{12}\,h\,r_0\,t^3+$
$26\,C_1\,\pi^3\,L^{12}\,h\,t^4$

$\mathrm{a}_{t,1} = 192\,C_1\,\pi\,L^{13}\,b^2\,r_0\,t+96\,C_1\,\pi\,L^{13}\,b^2\,t^2+384\,C_1\,\pi\,L^{13}\,b\,h\,r_0\,t+192\,C_1\,\pi\,L^{13}\,b\,h\,t^2+$
$96\,C_1\,\pi^2\,L^{13}\,b\,{r_0}^2\,t+96\,C_1\,\pi^2\,L^{13}\,b\,r_0\,t^2+32\,C_1\,\pi^2\,L^{13}\,b\,t^3+192\,C_1\,\pi\,L^{13}\,h^2\,r_0\,t+$
$96\,C_1\,\pi\,L^{13}\,h^2\,t^2+96\,C_1\,\pi^2\,L^{13}\,h\,{r_0}^2\,t+96\,C_1\,\pi^2\,L^{13}\,h\,r_0\,t^2+32\,C_1\,\pi^2\,L^{13}\,h\,t^3+$
$16\,C_1\,\pi^3\,L^{13}\,{r_0}^3\,t+24\,C_1\,\pi^3\,L^{13}\,{r_0}^2\,t^2+16\,C_1\,\pi^3\,L^{13}\,r_0\,t^3+4\,C_1\,\pi^3\,L^{13}\,t^4$

$\mathrm{b}_{t,8} = 24\,L^6\,b^8+192\,L^6\,b^7\,h+672\,L^6\,b^6\,h^2+1344\,L^6\,b^5\,h^3+1680\,L^6\,b^4\,h^4+$
$1344\,L^6\,b^3\,h^5+672\,L^6\,b^2\,h^6+192\,L^6\,b\,h^7+24\,L^6\,h^8$

$\mathrm{b}_{t,7} = 192\,L^7\,b^7+1344\,L^7\,b^6\,h+4032\,L^7\,b^5\,h^2+6720\,L^7\,b^4\,h^3+6720\,L^7\,b^3\,h^4+$
$4032\,L^7\,b^2\,h^5+1344\,L^7\,b\,h^6+192\,L^7\,h^7$

$\mathrm{b}_{t,6} = 672\,L^8\,b^6+4032\,L^8\,b^5\,h+10080\,L^8\,b^4\,h^2+13440\,L^8\,b^3\,h^3+10080\,L^8\,b^2\,h^4+$
$4032\,L^8\,b\,h^5+672\,L^8\,h^6$

$\mathrm{b}_{t,5} = 1344\,L^9\,b^5+6720\,L^9\,b^4\,h+13440\,L^9\,b^3\,h^2+13440\,L^9\,b^2\,h^3+6720\,L^9\,b\,h^4+$
$1344\,L^9\,h^5$

$\mathrm{b}_{t,4} = 1680\,L^{10}\,b^4+6720\,L^{10}\,b^3\,h+10080\,L^{10}\,b^2\,h^2+6720\,L^{10}\,b\,h^3+1680\,L^{10}\,h^4$

$\mathrm{b}_{t,3} = 1344\,L^{11}\,b^3+4032\,L^{11}\,b^2\,h+4032\,L^{11}\,b\,h^2+1344\,L^{11}\,h^3$

$\mathrm{b}_{t,2} = 672\,L^{12}\,b^2+1344\,L^{12}\,b\,h+672\,L^{12}\,h^2$

$\mathrm{b}_{t,1} = 192\,L^{13}\,b+192\,L^{13}\,h$

$\mathrm{b}_{t,0} = 24\,L^{14}$
